# The developmental transcriptome atlas of the spoon worm *Urechis unicinctus* (Echiurida: Annelida)

**DOI:** 10.1093/gigascience/giy007

**Published:** 2018-02-15

**Authors:** Chungoo Park, Yong-Hee Han, Sung-Gwon Lee, Kyoung-Bin Ry, Jooseong Oh, Elizabeth M A Kern, Joong-Ki Park, Sung-Jin Cho

**Affiliations:** 1 School of Biological Sciences, College of Natural Sciences, Chungbuk National University, Cheongju, Chungbuk 28644, Republic of Korea; 2 School of Biological Sciences and Technology, Chonnam National University, Gwangju 61186, Republic of Korea; 3 Division of EcoScience, Ewha Womans University, Seoul 03760, Republic of Korea

**Keywords:** *Urechis unicinctus*, *Echiurida*, developmental transcriptome, RNA-Seq, *de novo* assembly

## Abstract

**Background:**

Echiurida is one of the most intriguing major subgroups of annelida because, unlike most other annelids, echiurids lack metameric body segmentation as adults. For this reason, transcriptome analyses from various developmental stages of echiurid species can be of substantial value for understanding precise expression levels and the complex regulatory networks during early and larval development.

**Results:**

A total of 914 million raw RNA-Seq reads were produced from 14 developmental stages of *Urechis unicinctus* and were *de novo* assembled into contigs spanning 63,928,225 bp with an N50 length of 2700 bp. The resulting comprehensive transcriptome database of the early developmental stages of *U. unicinctus* consists of 20,305 representative functional protein-coding transcripts. Approximately 66% of unigenes were assigned to superphylum-level taxa, including Lophotrochozoa (40%). The completeness of the transcriptome assembly was assessed using benchmarking universal single-copy orthologs; 75.7% of the single-copy orthologs were presented in our transcriptome database. We observed 3 distinct patterns of global transcriptome profiles from 14 developmental stages and identified 12,705 genes that showed dynamic regulation patterns during the differentiation and maturation of *U. unicinctus* cells.

**Conclusions:**

We present the first large-scale developmental transcriptome dataset of *U. unicinctus* and provide a general overview of the dynamics of global gene expression changes during its early developmental stages. The analysis of time-course gene expression data is a first step toward understanding the complex developmental gene regulatory networks in *U. unicinctus* and will furnish a valuable resource for analyzing the functions of gene repertoires in various developmental phases.

## Data Description

### Background

Within the major annelid groups, Echiurida (also called “marine spoon worms”) is represented by a morphologically and ontogenetically unique assemblage that includes approximately 165 species, most of which lack segmentation as adults. However, they possess annelid-like morphological and developmental features, including the organization of the larval nervous system [1, 2]. They were once considered a separate metazoan phylum. However, reevaluation of morphological and molecular data indicated that Echiurida is nested within Annelida, which represents one of the three major animal phyla with body segmentation [3–7]. In this respect, transcriptome analyses from various developmental stages of echiurid species are of substantial value for understanding precise expression levels and the complex regulatory networks involved in early and larval development. Indeed, data from recently published developmental transcriptomes of other Lophotrochozoans (e.g., *Aplysia californica* and *Platynereis dumerilii*) have highlighted insights into molecular mechanisms underlying early development and metamorphosis [8, 9].

**Figure 1:**
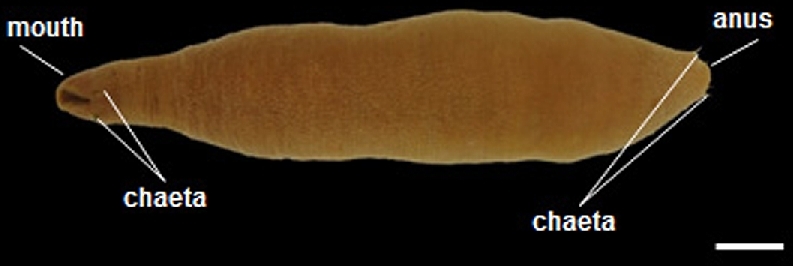
Adult *Urechis unicinctus* used in this study (proboscis retracted). Scale bar; 1 cm.

*Urechis unicinctus* is an echiuran species that inhabits burrows in soft sediments in intertidal areas (Fig. 1). The *Urechis* genus may hold important clues to the genetic basis of the evolutionary gain and loss of segmentation due to its nested position within Annelida (i.e., sister to capitellid polychaetes), a lophotrochozoan phylum that is represented by a diverse group of segmented worms [4, 7]. However, current knowledge is limited on the molecular mechanisms that underlie the ontogeny of *U. unicinctus*. The goal of this study is to enhance our understanding of gene expression during embryonic development. Here, we report the transcriptome profiles (generated with the Illumina HiSeq platform) of developing embryos of *U. unicinctus*. Transcriptome sequencing data assist in the discovery of the roles of genes involved in various embryological and larval development processes. As the first large-scale transcriptomic dataset for *U. unicinctus*, this resource will help in the validation of development-specific gene features predicted by the genome.

### Sample collection, embryo culture, and RNA isolations

Adults of *U. unicinctus* were collected from intertidal mud flats on the southern coast of South Korea. We extracted eggs and sperms from 1 adult female and 1 male. To obtain *U. unicinctus* embryos, artificial fertilization was performed by mixing the appropriate ratio of sperms and eggs.

Embryos were reared in artificial seawater (reef crystals from Aquarium Systems, France) in a plastic case at room temperature (18°C–20°C). The late trochophore, a typical larval stage in which the intestinal tract is formed, was fed with a microalgae called *Isochrysis galbana*. Reared embryo samples were collected at each of the following stages: 0 hour (unfertilized egg), 0.5 hours post-fertilization (fertilized egg), polar body cell, 2 cell, 4 cell, 8 cell, 16 cell, 32 cell, blastula, emerged cilia, early trochophore (day 1), middle trochophore (day 2), late trochophore (day 5), and segmentation stage (day 30–45). Diagnostic features for each of the 3 trochophore stages are as follows. The early trochophore is a nonfeeding stage. In the middle trochophore, the gastrointestinal valve opens and the anus appears. In the late trochophore, the longer cilia of the apical tufts are replaced by shorter cilia that cover a greater area, and the prototroch cilia are longer. These developmental stages follow Newby's classification [10].

Total RNA was isolated from the embryos of the above samples using TRIZOL reagent (Invitrogen, Carlsbad, California) following the manufacturer's instructions. The purity and integrity of the total RNA isolated from each embryo sample were examined using a Nanodrop 2000C spectrophotometer (Thermo Scientific, Waltham, Massachusetts) and Bioanalyzer 2100 (Agilent Technologies, Palo Alto, California). Adult images were taken on a Canon EOS 550D, and embryo bright-field images were taken on a Leica DM6 B microscope using differential interference contrast (DIC) optics.

### TruSeq Stranded Ribo-Zero library preparation and sequencing

**Figure 2:**
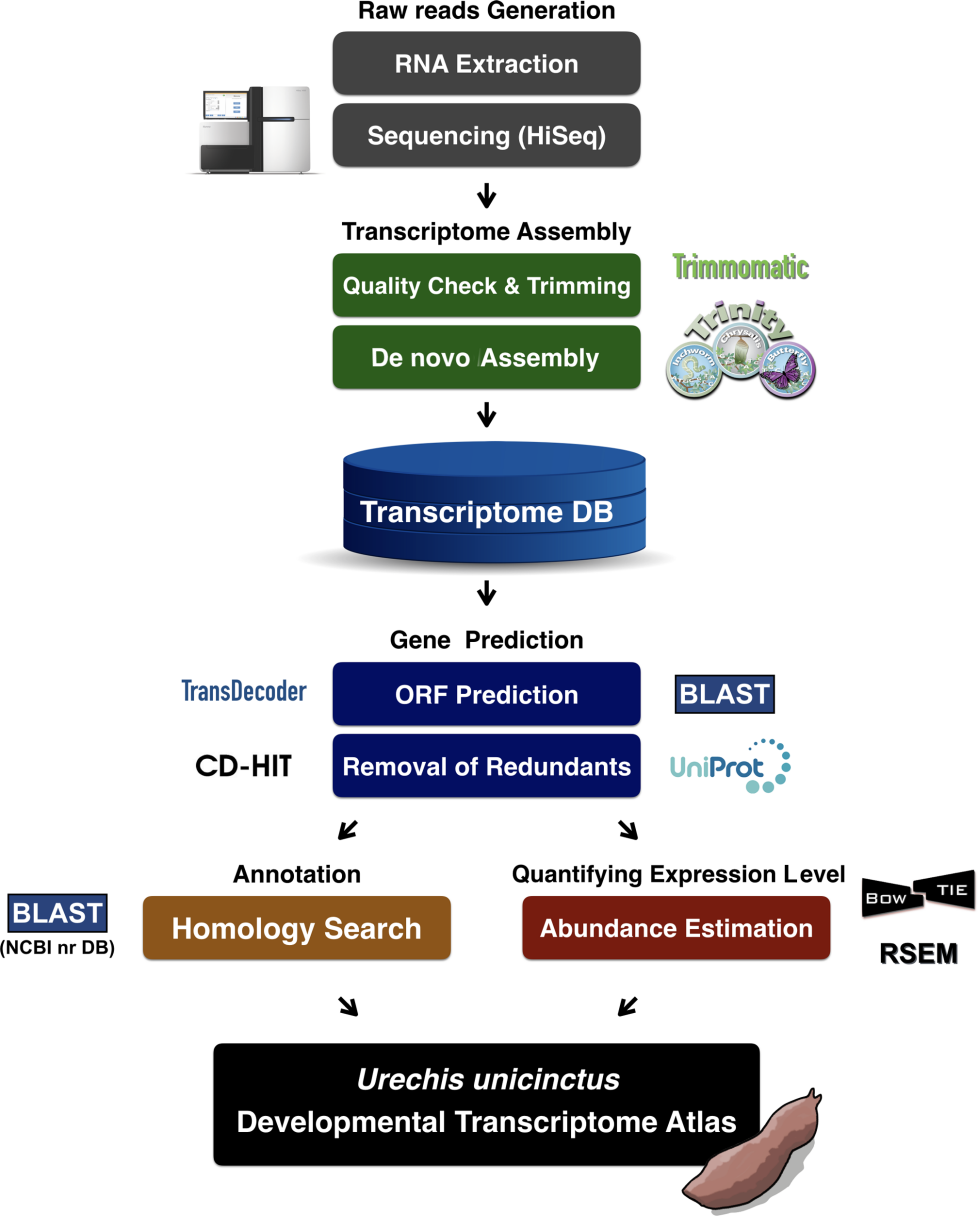
Schematic diagram of *Urechis unicinctus* transcriptome analysis in this study.

Total RNA concentration was calculated using Quant-IT RiboGreen (Invitrogen, R11490). To assess the integrity of the total RNA, samples were run on TapeStation RNA screentape (Agilent, 5067–5576). Only high-quality RNA preparations, with a RNA Integrity Number greater than 7.0, were used for RNA library construction. A library was independently prepared with 1 μg of total RNA for each sample using an Illumina TruSeq Stranded Total RNA Sample Prep Kit (Illumina, Inc., San Diego, California). The rRNA in total RNA was depleted using a Ribo-Zero kit. After the rRNA was depleted, the remaining RNA was purified, fragmented, and primed for cDNA synthesis. The cleaved RNA fragments were copied into first-strand cDNA using reverse transcriptase and random hexamers. This was followed by second-strand cDNA synthesis using DNA Polymerase I, RNase H, and dUTP. These cDNA fragments then underwent an end repair process, the addition of a single “A” base, and ligation of the adapters. The products were then purified and enriched with polymerase chain reaction (PCR) to create the final cDNA library. The libraries were quantified using quantitative PCR according to the qPCR Quantification Protocol Guide (KAPA Library Quantification kits for Illumina Sequencing platforms) and qualified using the TapeStation D1000 ScreenTape (Agilent Technologies, Waldbronn, Germany). The resulting samples were sequenced on the Illumina HiSeq 2000 system with a paired-end read with 101 cycles or the Illumina HiSeq 4000 system with a paired-end read with 151 cycles (Table 1). The experimental procedures and complete assembly pipeline are summarized in Fig. 2.

**Table 1:** Reads statistics

Samples	Total produced bases (bp)	Number of reads	Read length (bp)	guanine plus cytosine (GC) %	Q30%	Number of clean reads (%)
Oocyte	8749,299,078	57,942,378	151	43.87	90.53	54,583,372 (94.20)
Fertilized embryo	7204,375,496	47,711,096	151	43.86	92.32	45,817,358 (96.04)
Polar body	7553,516,790	50,023,290	151	41.40	91.12	47,401,970 (94.76)
2 cell	8663,957,200	57,377,200	151	40.21	92.63	55,263,572 (96.32)
4 cell	6693,881,642	44,330,342	151	40.88	90.81	43,001,172 (97.00)
8 cell	7417,271,000	49,121,000	151	42.14	92.31	46,360,492 (94.38)
16 cell	7993,095,608	52,934,408	151	41.52	91.75	50,571,562 (95.54)
32 cell	22,163,185,664	146,776,064	151	42.11	91.44	139,587,140 (95.10)
Blastula	8885,042,038	58,841,338	151	45.23	92.04	56,298,300 (95.68)
Emerged cilia	8077,246,398	53,491,698	151	44.18	89.83	50,401,516 (94.22)
Early trochophore	7354,720,616	72,819,016	101	45.90	96.02	72,513,798 (99.58)
Middle trochophore	7581,052,122	75,059,922	101	46.58	96.31	74,755,084 (99.59)
Late trochophore	7807,192,940	77,298,940	101	46.69	96.66	77,100,204 (99.74)
Segmentation	10,556,984,102	69,913,802	151	48.19	92.37	67,990,654 (97.25)

### Transcriptome preprocessing and *de novo* assembly

After completion of the sequencing run, to obtain high-quality clean reads from the raw data (i.e., removing those containing adapter sequences, poly-N sequences, or low-quality bases), we performed quality-based trimming and filtering using Trimmomatic, version 0.33 (Trimmomatic, RRID:SCR_011848) [11] with the parameters ILLUMINACLIP: TruSeq3-PE-2.fa:2:30:10 LEADING:3 TRAILING:3 SLIDINGWINDOW:4:15 MINLEN:36 for the 101 bp library (or MINLEN:50 for the 151 bp library). An average of 63 million clean reads per sample was obtained (Table 1).

Before *de novo* assembly, all clean reads were pooled without normalization of read abundance, even though the use of all merged reads may require progressively increasing assembly time and memory usage in order to obtain a comprehensive reference transcriptome database. The merged reads were used for *de novo* transcriptome assembly using Trinity, version 2.1.1 (Trinity, RRID:SCR_013048) [12] with default parameters. The resulting assembled transcriptome consisted of 620,490 transcripts with an N50 value of 846 bp (Table 2). After assembly, open reading frames (ORFs) were predicted using TransDecoder (version 3.0.0) (http://transdecoder.sourceforge.net). To maximize sensitivity for capturing ORFs, all transcripts were aligned against the Uniprot/Swiss-Prot database (http://www.uniprot.org) via BLASTP search with an *E*-value cutoff of 10^−5^. Next, ORF lengths <100 amino acids were discarded to avoid maintaining transcripts with poor evidence for protein-coding regions. Finally, redundant transcripts with more than 99% sequence identity were removed using CD-HIT (version 4.6.5) [13], producing 60,472 nonredundant ORFs. These sequences span 63,928,225 bp with an N50 length of 2,700 bp.

**Table 2:** Statistics for *Urechis unicinctus* transcriptome assembly

Samples	Total assembled bases (bp)	Number of assembled transcripts	N50 transcript length (bp) (min–max: median)	Number of non-redundant ORFs	Number of ORFs with NR blast hit (longest ORF per unigene)
Oocyte	45,868,755	26,569	2801 (201–26,298: 1105)	9684	7791
Fertilized embryo	43,996,849	28,361	2689 (201–26,298: 917)	9469	7561
Polar body	43,132,738	26,716	2626 (201–26,298: 1020)	9246	7380
2 cell	44,839,836	31,326	2412 (201–26,298: 917)	9139	7254
4 cell	47,675,420	23,122	3204 (201–26,298: 841)	9414	7567
8 cell	45,215,462	27,532	2564 (201–31,183: 1442)	9030	7220
16 cell	49,536,401	33,776	2470 (201–26,298: 871)	9470	7463
32 cell	58,598,783	38,718	2461 (201–26,298: 927)	11,193	8597
Blastula	50,083,677	30,553	3004 (201–31,183: 901)	10,994	8535
Emerged cilia	58,462,746	27,855	3320 (201–31,183: 1513)	12,153	9625
Early trochophore	64,464,321	38,443	3291 (201–36,191: 858)	12,980	10,034
Middle trochophore	72,767,170	42,797	3234 (201–36,191: 930)	14,482	11,001
Late trochophore	77,723,477	48,553	3081 (201–36,191: 837)	15,208	11,300
Segmentation	49,350,938	26,509	2740 (201–32,619: 1318)	11,883	9030
Total	368,166,154	620,490	846 (201–36,191: 322)	32,880	20,305

Abbreviation: ORF: open reading frame.

To quantify expression levels, the reads for each library were mapped independently to the reference *U. unicinctus* transcriptome sequences using Bowtie, version 2.2.6 (Bowtie, RRID:SCR_005476) [14]; expression levels of these transcripts were estimated with RSEM, version 1.2.26 (RSEM, RRID:SCR_013027) [15]. The unit of expression level is referred to as fragment per kilobase of transcript per million fragments mapped in our analyses.

### Annotation

**Figure 3:**
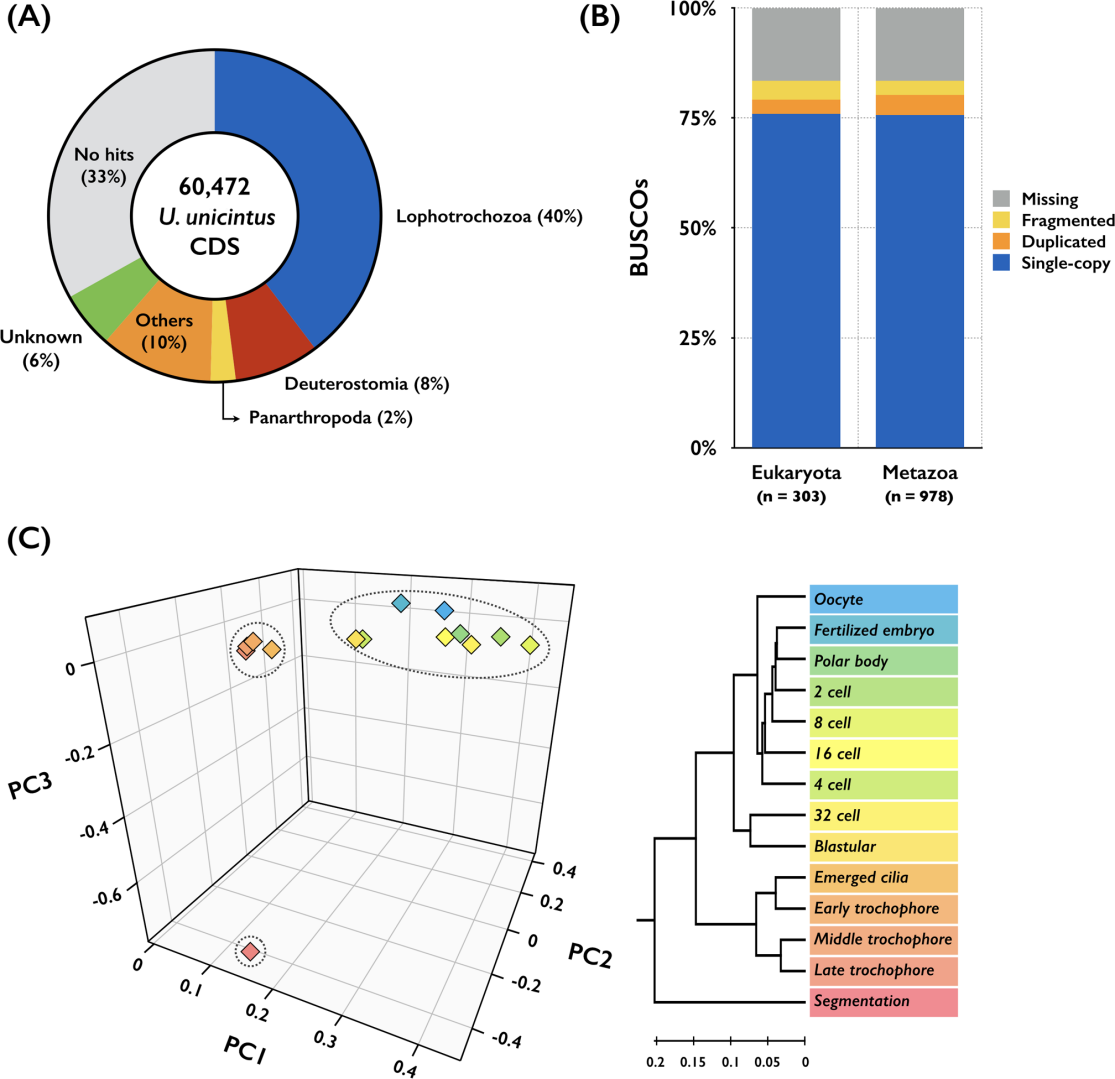
Analysis of *de novo* transcriptome and global gene expression patterns. A) Superphylum distribution for homology search of *Urechis unicinctus* coding sequences against the NR database using the best BLAST hit. B) Results of BUSCO analysis. C) Result of principal component analysis and a dendrogram of transcriptomes of 14 *U. unicinctus* developmental stages based on pairwise distance matrices (1 − ρ, Spearman correlation coefficient). The first, second, and third principal components account for 86.8, 6.8, and 5.9% of variance, respectively.

To annotate coding sequences (CDSs), the resulting 60,472 CDSs were compared against the NCBI nonredundant protein (NR) database (downloaded on 11 April 2017) using BLASTP with an *E*-value cutoff of 10^−10^ and the best BLAST hit. About 66% (40,111/60,472) of the CDS were assigned to superphylum-level taxa, including Lophotrochozoa (40%), Deuterostomia (8%), and Panarthropoda (2%) (Fig. 3A), which was to be generally expected. For further analysis, we excluded a number of CDSs (18%; 7,231/40,111) by using sequences derived from nonmetazoan taxa. When there were multiple coding sequences that mapped to the same gene in the NR database, the sequences with the longest CDS were first assigned to that gene. Based on this criterion, we established a comprehensive transcriptome database of 14 early developmental stages of *U. unicinctus* that comprises 20,305 representative functional protein-coding transcripts. We further assessed the completeness of the *U. unicinctus* development transcriptome using the program benchmarking universal single-copy orthologs, version 2.0 (BUSCO, RRID:SCR_015008) [16]. A total of 75.9% (230/303 genes) and 75.7% (740/978 genes) of the eukaryote and metazoan single-copy orthologs were identified, respectively (Fig. 3B).

### Transcriptome comparisons

To show that gene expression reflects development-specific differentiation and maturation processes, we built expression distance matrices for each developmental stage and constructed a gene expression tree (Fig. 3C). Two major transitions in expression patterns were observed: blastula to emerged cilia and late trochophore to segmentation. These transitions divided the 14 *U. unicinctus* developmental stages into 3 phases. The oocyte; polar body; fertilized; 2-, 4-, 8-, 16-, 32-cell embryo; and blastula stages make up phase I. The emerged cilia and early-, middle-, and late-trochophore stages make up phase II. The segmentation stage makes up phase III. These 3 distinct phases of global transcriptome profiles covering 14 developmental stages were supported by principal component analysis, which was performed using the “prcomp” function in the “stats” package in R (version 3.2.4) (Fig. 3C). These results suggest that developmental stages are well characterized by our transcription profiles and that the differential gene expression profiles presented in this study will be useful for further study of ontogenic processes at the gene expression level.

**Figure 4:**
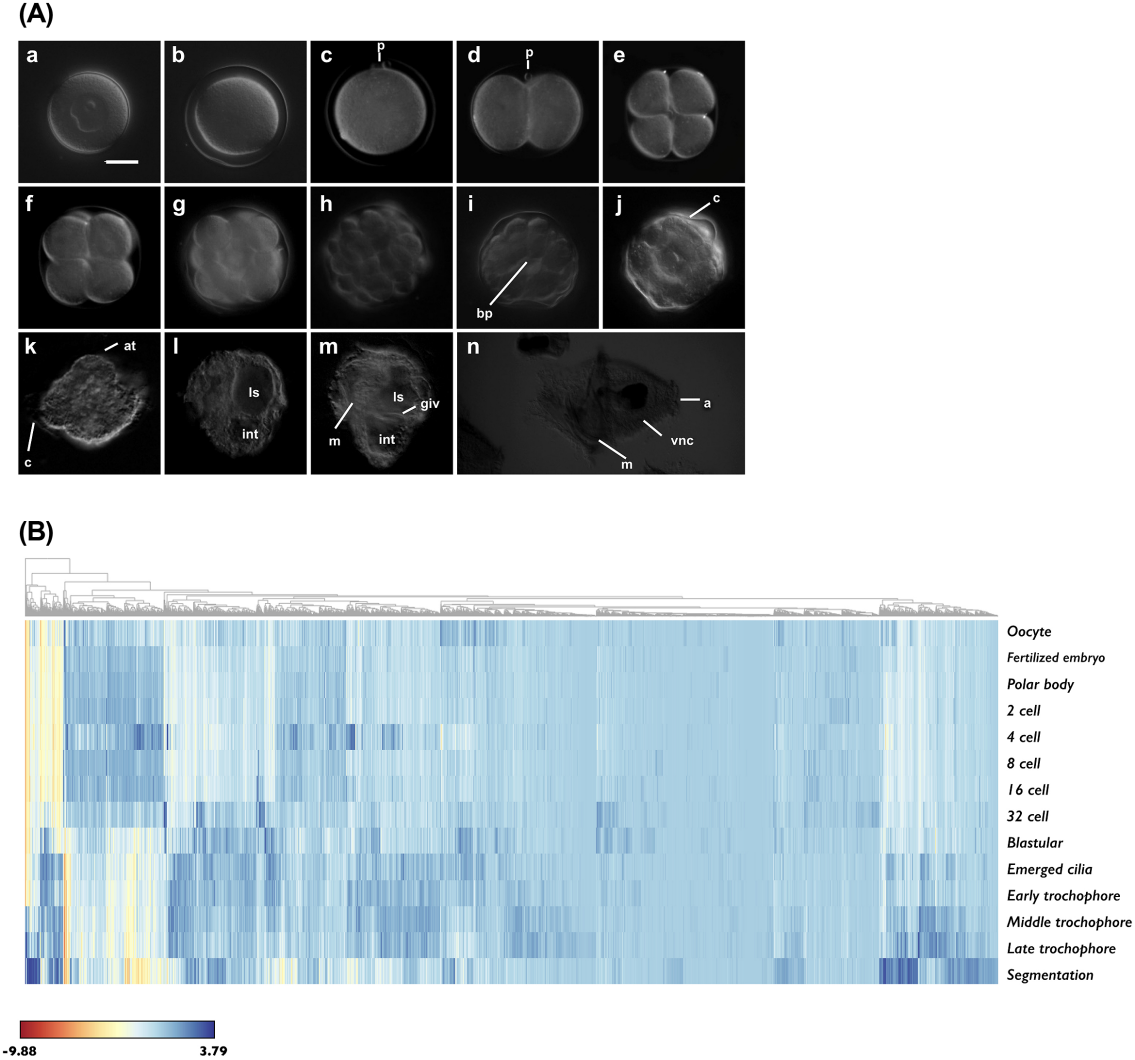
Representative images of *Urechis unicinctus* developmental stages and their gene expression profiles. A) Overview of *U. unicinctus* developmental stages. (a) oocyte, (b) fertilized embryo, (c) polar body, (d) 2 cell, (e) 4 cell, (f) 8 cell, (g) 16 cell, (h) 32 cell, (i) blastula, (j) emerged cilia, (k) early trochophore, (l) middle trochophore, (m) late trochophore, and (n) segmentation. p, polar body; bp, blastopore; c, cilia; ls, larval stomach; int, intestine; glv, gastrointestinal valve; m, mouth; vnc, ventral nerve cord; a, anus. Scale bar; 50μm. B) A heat map showing dynamic gene expression patterns with the relative expression levels (column) in each stage (row). Expression values (trimmed mean of M values) were log_2_-transformed and mean-centered by transcript. The hierarchical clustering was performed with Euclidean distances of gene expression values.

In an additional analysis, a gene whose expression level was significantly changed (≥10-fold and false discovery rate adjusted *P* value ≤ 0.1%) in at least one comparison was defined as a developmentally regulated gene. We identified 12,705 genes that showed dynamic regulation patterns during the differentiation and maturation of *U. unicinctus* cells (Fig. 4). Note that we used the trimmed mean of M values normalization [17] provided by the edgeR bioconductor package for R for this test.

Although this study presents the first large-scale developmental transcriptome dataset for a developmentally interesting animal group, *U. unicinctus* (Echiurida), the global landscape of its developmental transcriptome is not yet complete due to the lack of biological replicates and reference genome sequences.

In summary, we present the first large-scale, developmental, stage-specific transcriptome dataset for *U. unicinctus* and provide a general overview of the dynamics of global gene expression changes at different developmental stages. These data will fill an important gap in annelid-wide comparisons of gene expression patterns and will lead to a better understanding of gene repertoires involved in different developmental stages and of complex developmental gene regulatory networks.

## Availability of supporting data

All raw sequencing data used for assembly have been deposited in the NCBI database under the accession numbers SRX2999418–SRX2999431, associated with BioProject PRJNA394029. Additional data further supporting the results of this article, including the transcriptome assembly, annotations, and BUSCO results, can be found in the *GigaScienc*e repository, GigaDB [18].

## Abbreviations

BUSCO: benchmarking universal single-copy orthologs; CDS: coding sequence; ORF: open reading frame.

## Competing interests

All authors report no competing interests.

## Author contributions

C.P. and S.J.C. designed the study; J.K.P. contributed to the project coordination; Y.H.H., K.B.R., and S.J.C. performed the experiments; S.G.L., J.O., and C.P. analyzed the data and evaluated the conclusions; C.P., S.J.C., J.K.P., S.G.L., and E.M.A.K. wrote the paper; all authors read and approved the final manuscript.

## Supplementary Material

GIGA-D-17-00202_Original_Submission.pdf

GIGA-D-17-00202_Revision_1.pdf

GIGA-D-17-00202_Revision_2.pdf

Response_to_Reviewer_Comments_Original_Submission.pdf

Response_to_Reviewer_Comments_Revision_1.pdf

Reviewer_1_Report_(Original_Submission) -- Gaspar Jekely 06 Sep 2017 Reviewed

Reviewer_2_Report_(Original_Submission) -- Nathan Kenny 11 Sep 2017 Reviewed

Reviewer_2_Report_(Revision_1) -- Nathan Kenny 27 Nov 2017 Reviewed

Reviewer_3_Report_(Original_Submission) -- Torsten Struck 20 Sep 2017 Reviewed

Reviewer_3_Report_(Revision_1) -- Torsten Struck 04 Dec 2017 Reviewed
