# Supplementary material for: The developmental transcriptome atlas of the spoon worm Urechis unicinctus (Echiurida: Annelida)
Source: Gigascience. 2018 Feb 15;7(3):giy007. doi: 10.1093/gigascience/giy007 (PMC5863216; doi:10.1093/gigascience/giy007)
Supplement: GIGA-D-17-00202_Original_Submission.pdf [file giy007_giga-d-17-00202_original_submission.pdf]

GigaScience

The developmental transcriptome atlas of the spoon worm Urechis unicinctus (Echiurida: Annelida)

--Manuscript Draft--

**Manuscript Number:**	GIGA-D-17-00202
**Full Title:**	The developmental transcriptome atlas of the spoon worm Urechis unicinctus (Echiurida: Annelida)
**Article Type:**	Data Note
**Funding Information:**	Ministry of Oceans and Fisheries (20140428) — Dr Joong-Ki Park
**Abstract:**	Background: Echiurida is one of the most intriguing major subgroups of the phylum Annelida, because unlike other annelid members, most echiurids lack metameric body segmentation as adults. For this reason, transcriptome analyses from various developmental stages of Echiurida species can be of substantial value for understanding how gene repertoires are involved in early stages of development,ontogenic morphogenesis and the formation of an unsegmented body plan.<br>Finding: A total of 914 million raw RNA-Seq reads were produced from 14 developmental stages of Urechis unicinctus, and were de novo assembled into contigs spanning 63,928,225 bp with an average length of 1,481 bp. The resulting comprehensive transcriptome database of the early developmental stages of U. unicinctus consists of 25,986 representative protein-coding functional transcripts. Approximately 66 % of unigenes were assigned to superphylum-level taxa, including Lophotrochozoa (40%). The completeness of the transcriptome assembly was assessed using BUSCO, and 71.3 % of the metazoan single-copy orthologs were presented in our transcriptome database. We observed three distinct patterns of global transcriptome profiles from 14 developmental stages, and identified a total of 12,910 genes that showed dynamic regulation patterns during the differentiation and maturation of U. unicinctus cells.<br>Conclusions: We present the first large-scale developmental transcriptome dataset of U. unicinctus and provide a general overview of the dynamics of global gene expression changes during its early developmental stages These data are a first step toward understanding the complex developmental gene regulatory networks in U. unicinctus, and will furnish a valuable resource for analyzing the functions of gene repertoires in various developmental phases.
**Corresponding Author:**	Sung-Jin Cho, Ph.D.<br>Chungbuk National University<br>Cheongju, Chungbuk KOREA, REPUBLIC OF
**Corresponding Author Secondary Information:**	
**Corresponding Author's Institution:**	Chungbuk National University
**Corresponding Author's Secondary Institution:**	
**First Author:**	Sung-Jin Cho, Ph.D.
**First Author Secondary Information:**	
**Order of Authors:**	Sung-Jin Cho, Ph.D.
	Yong-Hee Han
	Sung-Gwon Lee
	Kyoung-Bin Ryu
	Jooseong Oh
	Elizabeth Kern, Ph.D.

Powered by Editorial Manager® and ProduXion Manager® from Aries Systems Corporation

	Joong-Ki Park, Ph.D.
	Chungoo Park, Ph.D.
**Order of Authors Secondary Information:**	
**Opposed Reviewers:**	
**Additional Information:**	
**Question**	**Response**
Are you submitting this manuscript to a special series or article collection?	No
**Experimental design and statistics**<br><br>Full details of the experimental design and statistical methods used should be given in the Methods section, as detailed in our Minimum Standards Reporting Checklist. Information essential to interpreting the data presented should be made available in the figure legends.<br><br>Have you included all the information requested in your manuscript?	Yes
**Resources**<br><br>A description of all resources used, including antibodies, cell lines, animals and software tools, with enough information to allow them to be uniquely identified, should be included in the Methods section. Authors are strongly encouraged to cite Research Resource Identifiers (RRIDs) for antibodies, model organisms and tools, where possible.<br><br>Have you included the information requested as detailed in our Minimum Standards Reporting Checklist?	Yes
**Availability of data and materials**<br><br>All datasets and code on which the conclusions of the paper rely must be either included in your submission or deposited in publicly available repositories (where available and ethically appropriate), referencing such data using a unique identifier in the references and in the "Availability of Data and Materials" section of your manuscript.<br><br>Have you have met the above requirement as detailed in our Minimum	Yes

Standards Reporting Checklist?	

Powered by Editorial Manager® and ProduXion Manager® from Aries Systems Corporation

Manuscript Click here to download Manuscript Han et al-MS.docx

Data Note

The developmental transcriptome atlas of the spoon worm *Urechis unicinctus* (Echiurida: Annelida)

Yong-Hee Han[1#], Sung-Gwon Lee[2#], Kyoung-Bin Ryu[1#], Jooseong Oh[2], Elizabeth M. A. Kern[3], Joong-Ki Park[3*], Chungoo Park[2*], Sung-Jin Cho[1*]

[1]School of Biological Sciences, College of Natural Sciences, Chungbuk National University, Cheongju, Chungbuk 28644, Republic of Korea

[2]School of Biological Sciences and Technology, Chonnam National University, Gwangju 61186, Republic of Korea

[3]Division of EcoScience, Ewha Womans University, Seoul 03760, Republic of Korea

[#] These authors contributed equally to this work.

*Corresponding Authors.

E-mail addresses:

Sung-Jin Cho, sjchobio@chungbuk.ac.kr. Tel: +82-43-261-2294. Fax: +82-43-260-2298.

Chungoo Park, chungoo@jnu.ac.kr. Tel: +82-62-530-1913. Fax: +82-62-530-2199

Joong-Ki Park, jkpark@ewha.ac.kr. Tel: +82-2-3277-5948. Fax: +82-2-3277-2385.

Abstract

Background: Echiurida is one of the most intriguing major subgroups of the phylum Annelida, because unlike other annelid members, most echiurids lack metameric body segmentation as adults. For this reason, transcriptome analyses from various developmental stages of Echiurida species can be of substantial value for understanding how gene repertoires are involved in early stages of development,ontogenic morphogenesis and the formation of an unsegmented body plan.

Finding: A total of 914 million raw RNA-Seq reads were produced from 14 developmental stages of *Urechis unicinctus*, and were *de novo* assembled into contigs spanning 63,928,225 bp with an average length of 1,481 bp. The resulting comprehensive transcriptome database of the early developmental stages of *U. unicinctus* consists of 25,986 representative protein-coding functional transcripts. Approximately 66 % of unigenes were assigned to superphylum-level taxa, including Lophotrochozoa (40%). The completeness of the transcriptome assembly was assessed using BUSCO, and 71.3 % of the metazoan single-copy orthologs were presented in our transcriptome database. We observed three distinct patterns of global transcriptome profiles from 14 developmental stages, and identified a total of 12,910 genes that showed dynamic regulation patterns during the differentiation and maturation of *U. unicinctus* cells.

Conclusions: We present the first large-scale developmental transcriptome dataset of *U. unicinctus* and provide a general overview of the dynamics of global gene expression changes during its early developmental stages These data are a first step toward understanding the complex developmental gene regulatory networks in *U. unicinctus*,

and will furnish a valuable resource for analyzing the functions of gene repertoires in various developmental phases.

Keywords: *Urechis unicinctus*, Echiurida, Developmental transcriptome, RNA-Seq, *de novo* assembly

Data Description

Background

Within the major annelid groups, Echiurida (also called the ‘marine spoon worms’) is represented by a morphologically and ontogenetically unique assemblage that includes approximately 165 species, most of which lacks segmentation in adults, although they possess annelid-like morphological and developmental features including a larval nerve system [1]. They were once considered a separate metazoan phylum, but reevaluation of morphological and molecular data indicated that Echiurida is a subgroup of Annelida (nested within polychaetes) [1, 2]. In this respect, transcriptome analyses from various developmental stages of Echiurida species are of substantial value for understanding how gene repertoires are involved in early stages of development, in particular in ontogenic morphogenesis and the formation of an unsegmented body.

Urechis unicinctus is an echiuran species that inhabit burrows in intertidal soft sediments (Fig. 1).. This species has attracted special attention in evolutionary developmental biology (‘evo-devo’) due to its position within the Lophotrochzoa, a clade that includes a diverse group of animal phyla with different body architectures and larval development. However, knowledge is limited on the molecular mechanisms that underlie the ontogeny of *U. unicinctus*. The goal of this study is to enhance our understanding of gene expression during embryonic development. Here we report the transcriptome profiling of developing embryos of *U. unicinctus*, using the Illumina HiSeq^{TM} platform. Transcriptome sequencing data provides an invaluable resource for the discovery of the roles of genes involved in various embryological and larval development processes in *U. unicinctus*. Finally, this first large-scale transcriptomic

dataset will help in the validation of development-specific gene features predicted by the genome.

Embryo culture, sample collection, and RNA isolation

Adults of *Urechis unicinctus* were collected from sandy bottom at the southern coast of South Korea, and were subjected to artificial insemination. Adult worms were dissected and the eggs and sperm were taken from the gonads of each female and male and transferred to distilled seawater. Artificial fertilization was performed by mixing the appropriate ratio of sperms and eggs. *Urechis unicinctus* embryos were reared in artificial seawater in a plastic case at room temperature (22-24 °C). The late trochophore, a typical larval stage in which the intestinal tract is formed, was fed with a microalgae called *Isochrysis galbana*. Reared embryo samples were collected at the following stages: 0 h (unfertilized egg), 0.5 h post fertilization (fertilized egg), polarbody cell, 2 cell, 4 cell, 8 cell, 16 cell, 32 cell, blastula, emerged cilia, early trochophore (day 1), middle trochophore (day 2), late trochophore (day 5), and segmentation stage (day 30~45).

Total RNA was isolated from the embryos of the above samples using TRIZOL reagent (Invitrogen, Carlsbad, CA, USA) following the manufacturer's instructions. The purity and integrity of the total RNA isolated from each embryo sample were examined using a Nanodrop 2000C spectrophotometer (Thermo Scientific, Waltham, MA, USA) and Bioanalyzer 2100 (Agilent Technologies, Palo Alto CA, USA). Adult images were taken on a Canon EOS 550D, and embryo bright-field images were taken on a Leica DM6 B microscope using DIC optics.

Library preparation and sequencing

One microgram of purified total RNA was used with the TruSeq RNA library preparation kit (Illumina, San Diego, USA) following the manufacturer's instructions. Briefly, the mRNAs were purified from the total RNA using poly-T oligonucleotide-attached magnetic beads and were chemically fragmented and reverse transcribed into single-stranded cDNAs using random hexamer priming. The second-strand cDNAs were synthesized and end-repaired. The resulting double-stranded cDNA fragments were ligated to adapter sequences. After gel purification, PCR-amplified cDNA libraries were assessed for quality and quantity on the Illumina qPCR Quantification Protocol Guide and Agilent Technologies 2100 Bioanalyzer (Agilent Technologies, Palo Alto, CA, USA). The resulting samples were sequenced on the Illumina HiSeq 2000 system with a paired-end read with 101 cycles or the Illumina HiSeq 4000 system with a paired-end read with 151 cycles (Table 1). The experimental procedures and complete assembly pipeline are summarized in Fig. 2.

Transcriptome preprocessing and *de novo* assembly

After completion of the sequencing run, to obtain high-quality clean reads (i.e., removing those containing adapter sequences, poly-N sequences, or low quality bases) from the raw data, we performed quality-based trimming and filtering using Trimmomatic (version 0.33) [3] with the parameters ILLUMINACLIP:TruSeq3-PE-2.fa:2:30:10 LEADING:3 TRAILING:3 SLIDINGWINDOW:4:15 MINLEN:36 for the 101 bp library (or MINLEN:50 for the 151 bp library). An average of 63 million clean reads per sample was obtained (Table 1). Based on the estimated *U. unicinctus* genome

size of   1.4 Gb using k-mer analysis (Fig. 3A) in this study, we produced an average of 6.8X coverage per sample. This coverage is ~ 3.8-fold higher than the minimum recommended coverage (1.8X) to sufficiently detect all expressed genes in the genome [4], indicating that our high sequencing depth had a significant impact on measuring expression levels of most *U. unicinctus* development-associated genes, even extremely low-abundance genes.

Before *de novo* assembly, all clean reads were concatenated without normalization of read abundance, even though the use of all merged reads may require progressively increasing assembly time and memory usage, in order to obtain a comprehensive reference transcriptome database. The merged reads were used for *de novo* transcriptome assembly using Trinity (version 2.1.1) [5] with default parameters. The resulting assembled transcriptome consisted of 620,490 transcripts with an N50 value of 846 bp (Table 2). After assembly, open reading frames (ORFs) were predicted using TransDecoder (version 3.0.0) (http://transdecoder.sourceforge.net). To maximize sensitivity for capturing ORFs, all transcripts were aligned against the Uniprot/Swiss-Prot database (http://www.uniprot.org) via BLASTP search with an *E*-value cutoff of 10^{-5}. Next, ORF length < 100 amino acids were discarded to avoid maintaining transcripts with poor evidence for protein-coding regions. Finally, redundant transcripts with more than 99% sequence identity were removed using CD-HIT (version 4.6.5) [6], producing a total of 60,472 non-redundant ORFs. These sequences span 63,928,225 bp with an average length of 1,481 bp.

To quantify expression levels, the reads for each library were mapped independently to the reference *U. unicinctus* transcriptome sequences using bowtie

(version 2.2.6) [7], and expression levels of these transcripts were estimated with RSEM (version 1.2.26) [8]. The unit of expression level is referred to as *f*ragment *p*er *k*ilobase of transcript per *m*illion fragments mapped (FPKM) in our analyses.

Annotation

To annotate coding sequences (CDS), the resulting 60,472 CDSs were compared against the NCBI non-redundant protein (NR) database (downloaded on April 11, 2017) using BLASTP with an *E*-value cutoff of 10^{-10}. About 66 % (40,111/60,472) of the CDS were assigned to superphylum-level taxa including Lophotrochozoa (40%), Deuterostomia (8%), and Panarthropoda (2%) (Fig. 3B), which is to be generally expected. When there were multiple coding sequences that mapped to the same gene in the NR database, the sequences with the longest CDS were first assigned to that gene. Based on this criterion, we established a comprehensive transcriptome database of 14 early developmental stages of *U. unicinctus* that comprises 25,986 representative protein-coding functional transcripts. We further assessed the completeness of the *U. unicinctus* development transcriptome using the program BUSCO (bench-marking universal single-copy orthologs) (version 2.0) [9]. 69.3 (210 / 303 genes), and 71.3 % (697 / 978 genes) of the eukaryote and metazoan single-copy orthologs were identified respectively (Fig. 3C). Our overall BUSCO scores are higher than the corresponding value (~ 50 %) estimated by the percentage of development-specific expressed genes in several model species [10-13]. Although this result is not directly comparable because of the different sources and the different sets of core genes, we may be confident that our *U. unicinctus* development transcriptome is sufficiently complete to allow

conclusions concerning global developmental changes in gene expression.

Transcriptome comparisons

To show the gene expression reflects development-specific differentiation and maturation processes, we built expression distance matrices for each developmental stage and constructed a gene expression tree (Fig. 3D). Two major transitions in expression patterns were observed: (1) blastula to emerged cilia and (2) late trochophore to segmentation. These transitions divided the 14 *U. unicinctus* developmental stages into three phases: the oocyte, polar body, fertilized, 2-, 4-, 8-, 16-, 32-cell embryo, and blastula stages make up Phase I; the emerged cilia, early-, middle-, and late-trochophore stages compose Phase II; and the segmentation stage makes up to Phase III. These three distinct phases of global transcriptome profiles covering 14 developmental stages were supported by principal component analysis (PCA), which was performed using the "prcomp" function in the "stats" package in R (version 3.2.4) (Fig. 3D). These results suggest that developmental stages are well characterized by our transcription profiles, Differential gene expression profiles presented in this study will be useful for further study of ontogenic processes at the gene expression level.

In an additional analysis, genes whose expression levels were over 10-fold and significantly changed, with a false discovery rate (FDR) $\leq 0.1\%$, were defined as developmentally regulated genes. We identified a total of 12,910 such genes that showed dynamic regulation patterns during the differentiation and maturation of *U. unicinctus* cells (Fig. 4). Note that we used the TMM (trimmed mean of M values) normalization [14] provided by edgeR bioconductor package for R for this test.

In summary, we present the first large-scale, developmental stage-specific transcriptome dataset for *U. unicinctus*, and provide a general overview of the dynamics of global gene expression changes at different developmental stages. These data will fill an important gap in phylum-wide comparisons of gene expression patterns, and will lead to a better understanding of gene repertoires involved in different developmental stages and of complex developmental gene regulatory networks.

Availability of supporting data

All raw sequencing data used for assembly have been deposited in the NCBI database under the accession SRX2999418 to SRX2999431, associated with BioProject PRJNA394029. The additional dataset further supporting the results of this article can be found in the *GigaScienc*e repository, GigaDB.

Abbreviation

bp: base pairs; BUSCO: Bench-marking universal single-copy orthologs; CDS: Coding sequence; FDR: False discovery rate; FPKM: Fragments per kilobase of transcript per million mapped reads; Gb: Gigabases; ORFs: Open reading frames; PCA: Principal components analysis; RNA-Seq: High-throughput messenger RNA sequencing; TMM: Trimmed mean of M values.

Competing interests

The authors declare that they have no competing interests.

Authors' contribution

CP and SJC designed the study; JKP contributed to the project coordination; YHH, KBR, and SJC performed the experiments; SGL, JO, and CP analyzed the data and evaluated the conclusions; CP, SJC, JKP, SGL and EMAK wrote the paper; All authors read and approved the final manuscript.

Acknowledgements

This research was supported by a grant from the Collaborative Genome Program (20140428) funded by the Ministry of Oceans and Fisheries, Korea to CP, SJC, JKP.

Author details

[1]School of Biological Sciences, College of Natural Sciences, Chungbuk National University, Cheongju, Chungbuk 28644, Republic of Korea. [2]School of Biological Sciences and Technology, Chonnam National University, Gwangju 61186, Republic of Korea. [3]Division of EcoScience, Ewha Womans University, Seoul 03760, Republic of Korea

References

1. Struck TH, Schult N, Kusen T, Hickman E, Bleidorn C, McHugh D, et al. Annelid phylogeny and the status of Sipuncula and Echiura. BMC Evolutionary Biology. 2007;7 1:57. doi:10.1186/1471-2148-7-57.
2. Zrzavý J, Říha P, Piálek L and Janouškovec J. Phylogeny of Annelida (Lophotrochozoa): total-evidence analysis of morphology and six genes. BMC Evolutionary Biology. 2009;9 1:189. doi:10.1186/1471-2148-9-189.
3. Bolger AM, Lohse M and Usadel B. Trimmomatic: a flexible trimmer for Illumina sequence data. Bioinformatics. 2014;30 15:2114-20. doi:10.1093/bioinformatics/btu170.

4. Wang Y, Ghaffari N, Johnson CD, Braga-Neto UM, Wang H, Chen R, et al. Evaluation of the coverage and depth of transcriptome by RNA-Seq in chickens. BMC Bioinformatics. 2011;12 10:S5. doi:10.1186/1471-2105-12-S10-S5.
5. Grabherr MG, Haas BJ, Yassour M, Levin JZ, Thompson DA, Amit I, et al. Full-length transcriptome assembly from RNA-Seq data without a reference genome. Nat Biotech. 2011;29 7:644-52. doi:http://www.nature.com/nbt/journal/v29/n7/abs/nbt.1883.html - supplementary-information.
6. Fu L, Niu B, Zhu Z, Wu S and Li W. CD-HIT: accelerated for clustering the next-generation sequencing data. Bioinformatics. 2012;28 23:3150-2. doi:10.1093/bioinformatics/bts565.
7. Langmead B, Trapnell C, Pop M and Salzberg SL. Ultrafast and memory-efficient alignment of short DNA sequences to the human genome. Genome Biology. 2009;10 3:R25. doi:10.1186/gb-2009-10-3-r25.
8. Li B and Dewey CN. RSEM: accurate transcript quantification from RNA-Seq data with or without a reference genome. BMC Bioinformatics. 2011;12 1:323. doi:10.1186/1471-2105-12-323.
9. Simão FA, Waterhouse RM, Ioannidis P, Kriventseva EV and Zdobnov EM. BUSCO: assessing genome assembly and annotation completeness with single-copy orthologs. Bioinformatics. 2015;31 19:3210-2. doi:10.1093/bioinformatics/btv351.
10. Yi H, Xue L, Guo M-X, Ma J, Zeng Y, Wang W, et al. Gene expression atlas for human embryogenesis. The FASEB Journal. 2010;24 9:3341-50.
11. Vesterlund L, Jiao H, Unneberg P, Hovatta O and Kere J. The zebrafish transcriptome during early development. BMC Developmental Biology. 2011;11 1:30. doi:10.1186/1471-213X-11-30.
12. Hamatani T, Carter MG, Sharov AA and Ko MSH. Dynamics of Global Gene Expression Changes during Mouse Preimplantation Development. Developmental Cell. 2004;6 1:117-31. doi:http://dx.doi.org/10.1016/S1534-5807(03)00373-3.
13. Li JJ, Huang H, Bickel PJ and Brenner SE. Comparison of D. melanogaster and C. elegans developmental stages, tissues, and cells by modENCODE RNA-seq data. Genome Research. 2014;24 7:1086-101.
14. Robinson MD and Oshlack A. A scaling normalization method for differential expression analysis of RNA-seq data. Genome Biology. 2010;11 3:R25. doi:10.1186/gb-2010-11-3-r25.

Figure Legends

Figure 1. Adult worm of *Urechis unicinctus* used in this study (proboscis retracted). Scale bar; 1cm.

Figure 2. Schematic diagram of *U. unicinctus* transcriptome analysis in this study.

Figure 3. Analysis of *de novo* transcriptome and global gene expression patterns.

(A) A genomic k-mer distribution for genome size estimation of *U. unicinctus*. The lengths of K-mers were 17 (orange), 19 (yellow), 21 (green), 23 (blue) and 25 (purple). *U. unicinctus* genome size was estimated to be approximately 1.4 Gbp, independent of the k-mer size. **(B)** Superphylum distribution for homology search of *U. unicinctus* coding sequences against the NR database. (**C)** Results of BUSCO analysis. (**D)** Result of principal component analysis (PCA) and a phylogenetic tree based on pairwise distance matrices (1 - ρ, Spearman's correlation coefficient) of 14 *U. unicinctus* developmental stages. The first, second, and third principal components account for 84.9, 6.8, and 6.2 % of variance, respectively.

Figure 4. Representative images of *U. unicinctus* developmental stages and their gene expression profiles.

(A) Overview of *U. unicinctus* developmental stages. (a) oocyte, (b) fertilized embryo, (c) polar body, (d) 2 cell, (e) 4 cell, (f) 8 cell, (g) 16 cell, (h) 32 cell, (i) blastula, (j) emerged cilia, (k) early trochophore, (l) middle trochophore, (m) late trochophore, (n) segmentation. p, polar body; bp, blastopore; c, cilia; ls, larval stomach; int, intestine; glv, gastro-intestinal valve; m, mouse; vnc, ventral nerve cord; a, anus. Sclae bar; 50μm.(**B)**

A heat map showing dynamic gene expression patterns with the relative expression levels (column) in each stage (row). Expression values (TMM) were log2-transformed and median-centered by transcript. The hierarchical clustering was performed with Euclidean distances of gene expression values.

Table 1. Reads Statistics

Samples	Total produced bases (bp)	Number of reads	Read length (bp)	GC %	Q30 %	Number of clean reads (%)
Oocyte	8,749,299,078	57,942,378	151	43.87	90.53	54,583,372 (94.20)
Fertilized embryo	7,204,375,496	47,711,096	151	43.86	92.32	45,817,358 (96.04)
Polar body	7,553,516,790	50,023,290	151	41.40	91.12	47,401,970 (94.76)
2 cell	8,663,957,200	57,377,200	151	40.21	92.63	55,263,572 (96.32)
4 cell	6,693,881,642	44,330,342	151	40.88	90.81	43,001,172 (97.00)
8 cell	7,417,271,000	49,121,000	151	42.14	92.31	46,360,492 (94.38)
16 cell	7,993,095,608	52,934,408	151	41.52	91.75	50,571,562 (95.54)
32 cell	22,163,185,664	146,776,064	151	42.11	91.44	139,587,140 (95.10)
Blastula	8,885,042,038	58,841,338	151	45.23	92.04	56,298,300 (95.68)
Emerged cilia	8,077,246,398	53,491,698	151	44.18	89.83	50,401,516 (94.22)
Early trochophore	7,354,720,616	72,819,016	101	45.90	96.02	72,513,798 (99.58)
Middle trochophore	7,581,052,122	75,059,922	101	46.58	96.31	74,755,084 (99.59)
Late trochophore	7,807,192,940	77,298,940	101	46.69	96.66	77,100,204 (99.74)
Segmentation	10,556,984,102	69,913,802	151	48.19	92.37	67,990,654 (97.25)

 Click here to download Table Han et al-Table 2.docx

Table 2. Statistics for *Urechis unicinctus* transcriptome assembly

Samples	Total assembled bases (bp)	Number of assembled transcripts	N50 transcript length (bp) (min - max : median)	Number of non-redundant ORFs	Number of ORFs with NR blast hit (longest ORF per unigene)
Oocyte	45,868,755	26,569	2,801 (201 - 26,298 : 1,105)	9,062	6,511
Fertilized embryo	43,996,849	28,361	2,689 (201 - 26,298 : 917)	7,978	4,799
Polar body	43,132,738	26,716	2,626 (201 - 26,298 : 1,020)	7,369	5,611
2 cell	44,839,836	31,326	2,412 (201 - 26,298 : 917)	6,996	4,973
4 cell	47,675,420	23,122	3,204 (201 - 26,298 : 841)	8,519	5,111
8 cell	45,215,462	27,532	2,564 (201 - 31,183: 1,442)	7,397	5,491
16 cell	49,536,401	33,776	2,470 (201 - 26,298 : 871)	8,166	5,415
32 cell	58,598,783	38,718	2,461 (201 - 26,298 : 927)	10,672	6,018
Blastula	50,083,677	30,553	3,004 (201 - 31,183: 901)	11,175	7,038
Emerged cilia	58,462,746	27,855	3,320 (201 - 31,183: 1,513)	13,759	9,525
Early trochophore	64,464,321	38,443	3,291 (201 - 36,191: 858)	14,349	9,705
Middle trochophore	72,767,170	42,797	3,234 (201 - 36,191: 930)	16,874	10,650
Late trochophore	77,723,477	48,553	3,081 (201 - 36,191: 837)	18,845	11,656
Segmentation	49,350,938	26,509	2,740 (201 - 32,619: 1,318)	14,388	5,721
Total	368,166,154	620,490	846 (201 - 36,191 : 322)	60,472	25,986

Figure1

Click here to download Figure Figure1.tif

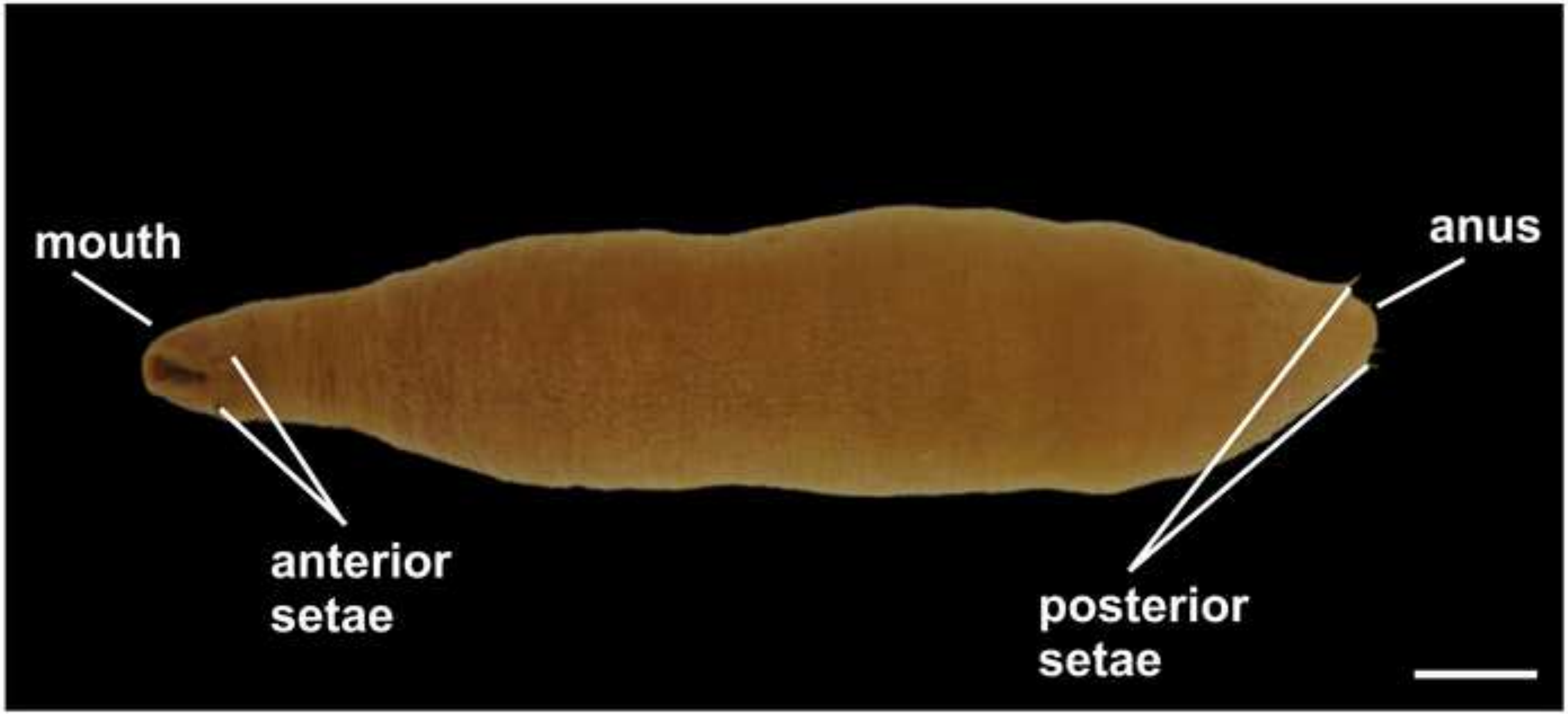

Figure2 Click here to download Figure Figure2.tif

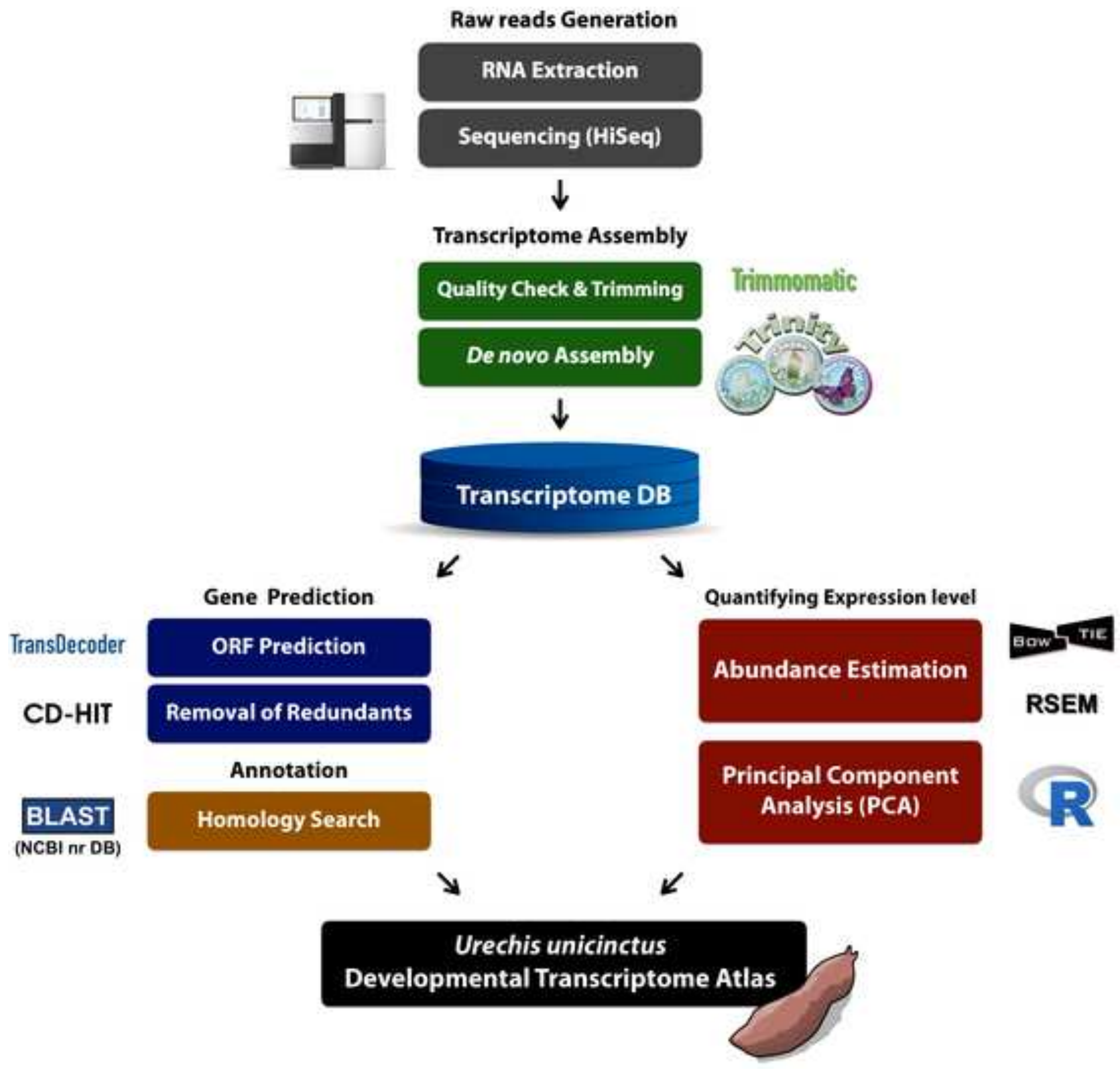

Figure3

Click here to download Figure Figure3.tif

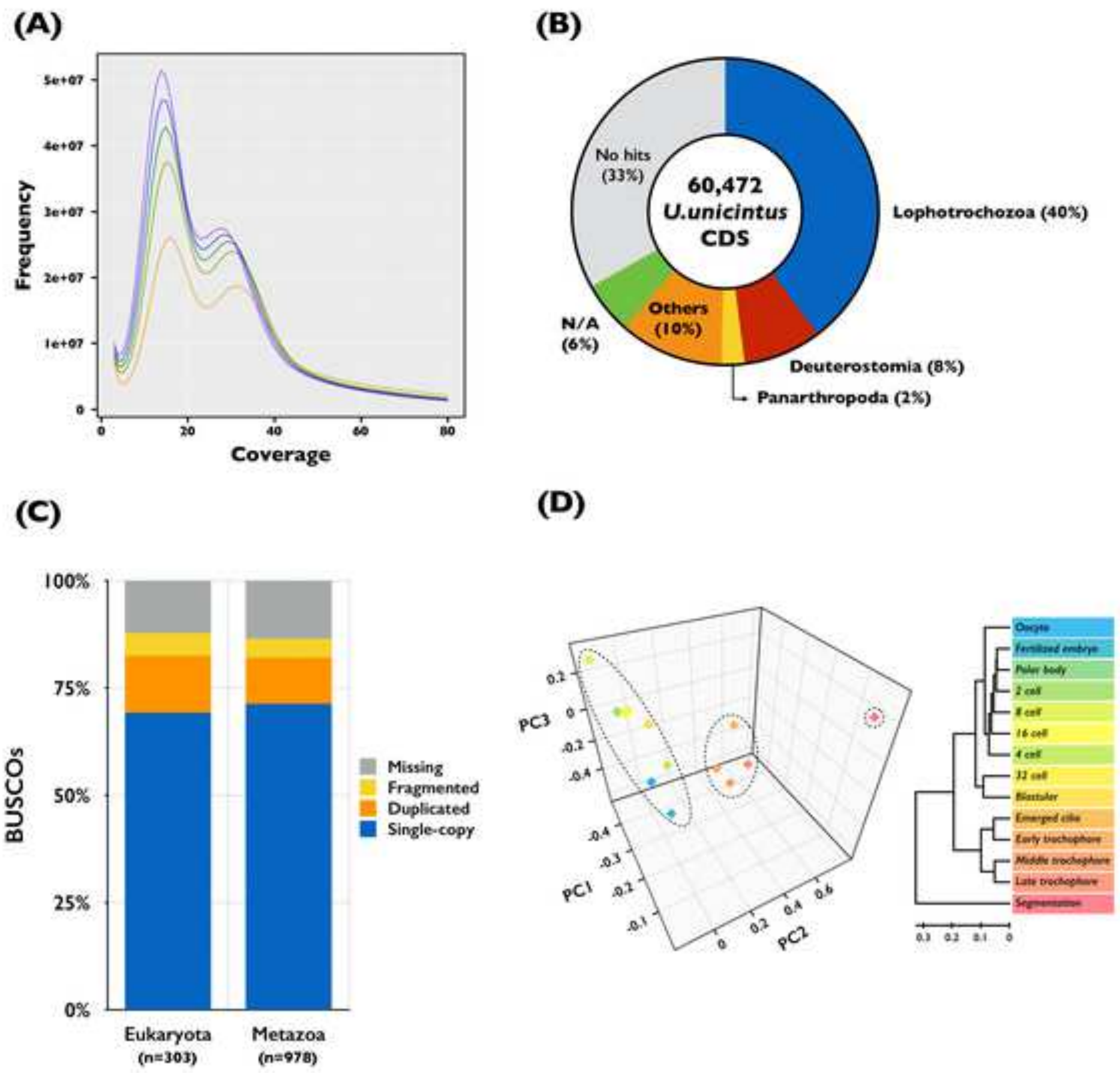

Figure4

Click here to download Figure figure4.tif

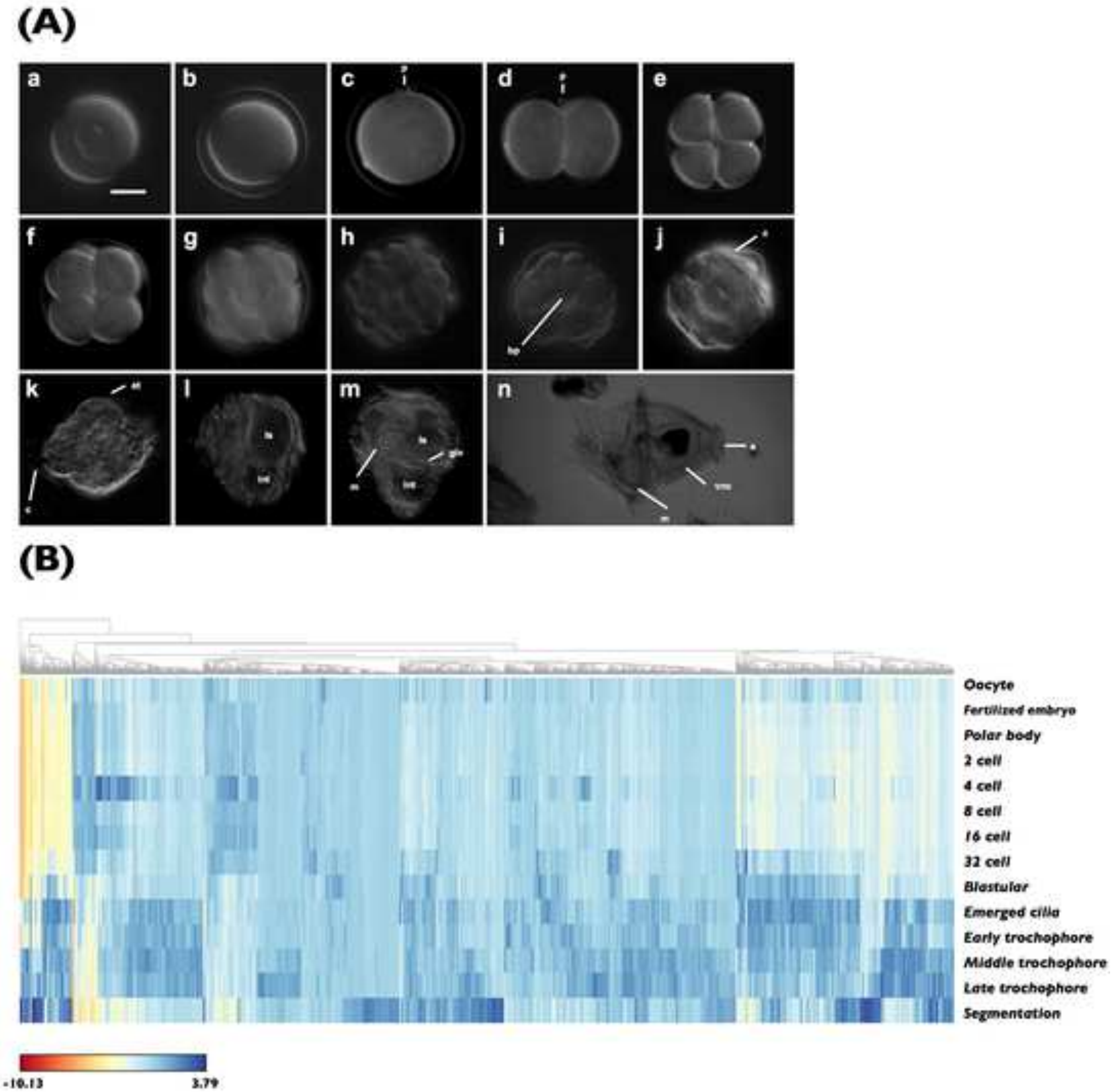

Personal Cover Click here to download Personal Cover Han et al-Cover letter.docx

Editor in Chief

GigaScience

Dear Editor,

Please consider our manuscript, “The developmental transcriptome atlas of the spoon worm *Urechis unicinctus* (Echiurida: Annelida)” for possible publication as a Data Note in GigaScience. Echiurida is one of the most intriguing major subgroups of the phylum Annelida in that unlike other annelid members, they lack metameric body segmentation at adult stage. This feature has attracted special attention in evolutionary developmental biology (‘evo-devo’), due to its phylogenetic position within the Lophotrochzoa, one of the metazoan superphyla that include a diverse group of animals with different body architectures (including metamerism) and early stages of larval development. In this study, we report the first transcriptome atlas of developing embryos of *U. unicinctus* sampled from 14 developmental stages. From this first large-scale developmental transcriptome dataset, we identified a total of 12,910 genes that show dynamic regulation patterns during the differentiation and maturation. We believe the transcriptome data presented here provide a key information on the gene repertoires and expression patterns that are essentially associated with the control of developmental fates in early embryonic stages.

We anticipate that this manuscript will help fill an important gap in phylum-wide comparisons of gene expression patterns and also will be of interest to scientists who work on evolutionary developmental biology and the genome biology of lophotrochozoan animals.

Thank you for your time and consideration.

Sincerely,

Sung-Jin Cho, Ph.D.

Associate Professor

School of Biological Sciences

College of Natural Sciences

Chungbuk National University

Republic of Korea

E-mail:sjchobio@chungbuk.ac.kr
