# Supplementary material for: The developmental transcriptome atlas of the spoon worm Urechis unicinctus (Echiurida: Annelida)
Source: Gigascience. 2018 Feb 15;7(3):giy007. doi: 10.1093/gigascience/giy007 (PMC5863216; doi:10.1093/gigascience/giy007)
Supplement: GIGA-D-17-00202_Revision_1.pdf [file giy007_giga-d-17-00202_revision_1.pdf]

GigaScience

The developmental transcriptome atlas of the spoon worm Urechis unicinctus (Echiurida: Annelida)

--Manuscript Draft--

**Manuscript Number:**	GIGA-D-17-00202R1
**Full Title:**	The developmental transcriptome atlas of the spoon worm Urechis unicinctus (Echiurida: Annelida)
**Article Type:**	Data Note
**Funding Information:**	Ministry of Oceans and Fisheries (20140428) — Dr Joong-Ki Park<br>Basic Science Research Program through the National Research Foundation of Korea (NRF-2016R1D1A1B03933412) — Dr Sung-Jin Cho
**Abstract:**	Background: Echiurida is one of the most intriguing major subgroups of the phylum Annelida, because unlike most other annelids, echiurids lack metameric body segmentation as adults. For this reason, transcriptome analyses from various developmental stages of Echiurid can be of substantial value for understanding precise expression levels and the complex regulatory networks during early and larval development.<br>Finding: A total of 914 million raw RNA-Seq reads were produced from 14 developmental stages of Urechis unicinctus, and were de novo assembled into contigs spanning 63,928,225 bp with an N50 length of 2,700 bp. The resulting comprehensive transcriptome database of the early developmental stages of U. unicinctus consists of 20,305 representative functional protein-coding transcripts. Approximately 66 % of unigenes were assigned to superphylum-level taxa, including Lophotrochozoa (40%). The completeness of the transcriptome assembly was assessed using BUSCO, and 75.7 % of the metazoan single-copy orthologs were presented in our transcriptome database. We observed three distinct patterns of global transcriptome profiles from 14 developmental stages, and identified a total of 12,705 genes that showed dynamic regulation patterns during the differentiation and maturation of U. unicinctus cells.<br>Conclusions: We present the first large-scale developmental transcriptome dataset of U. unicinctus and provide a general overview of the dynamics of global gene expression changes during its early developmental stages. The analysis of time-course gene expression data is a first step toward understanding the complex developmental gene regulatory networks in U. unicinctus, and will furnish a valuable resource for analyzing the functions of gene repertoires in various developmental phases.
**Corresponding Author:**	Sung-Jin Cho, Ph.D.<br>Chungbuk National University<br>Cheongju, Chungbuk KOREA, REPUBLIC OF
**Corresponding Author Secondary Information:**	
**Corresponding Author's Institution:**	Chungbuk National University
**Corresponding Author's Secondary Institution:**	
**First Author:**	Sung-Jin Cho, Ph.D.
**First Author Secondary Information:**	
**Order of Authors:**	Sung-Jin Cho, Ph.D.
	Yong-Hee Han
	Sung-Gwon Lee
	Kyoung-Bin Ryu
	Jooseong Oh

Powered by Editorial Manager® and ProduXion Manager® from Aries Systems Corporation

	Elizabeth Kern, Ph.D.
	Joong-Ki Park, Ph.D.
	Chungoo Park, Ph.D.
**Order of Authors Secondary Information:**	
**Response to Reviewers:**	Editor in Chief<br>GigaScience<br><br>RE: Ms. Ref. No.: GIGA-D-17-00202<br><br>Dear Editor:<br>Thank you very much for your letter on September 21, 2017.<br>Our manuscript (Ms. Ref. No.: GIGA-D-17-00202) entitled “The developmental transcriptome atlas of the spoon worm Urechis unicinctus (Echiurida: Annelida) ” has now been revised based on feedback from the reviewers and editor. With our resubmission we are including a detailed response to each of the reviewers' comments.<br>I hope all corrections and revisions in the revised manuscript will be satisfactory.<br><br>Sincerely yours,<br><br>Sung-Jin Cho, Ph.D.<br>Associate Professor<br>Department of Biology (S1-5, 204b)<br>College of Natural Sciences<br>Chungbuk National University<br>52 Naesudong-ro, Heungdeok-gu<br>Cheongju, Chungbuk 361-763<br>Republic of Korea<br>E-mail: sjchobio@chungbuk.ac.kr<br><br>Reviewer #1: In this study Han and colleagues report the early developmental transcriptome of the spoon worm Urechis unicinctus. This is a valuable resource that certainly will be used by the comparative genomics community and those working on annelid larval development.<br><br>I have a few comments to improve the manuscript:<br><br>Comment 1:<br>1) The authors seem to estimate the genome size of Urechis unicinctus based on k-mer analysis. However, for this they would need to use genomic reads, and not transcriptome reads. This estimate should be deleted, unless it was done on genomic sequences, in which case more detail should be given.<br><br>Authors’ response:<br>Although we utilized genomic reads to estimate the genome size of U. unicinctus, we deleted this section because reviewer #2 (comment 2; “It is also not directly relevant to a transcriptomic publication, as genome size is not necessarily reflective of transcriptomic complexity”) also pointed out same issue.<br><br>Comment 2:<br>2)The authors should compare their developmental transcriptome to other studies within (at least) the annelida or the Lophotrochozoa. Placing this study into context by listing other available resources would be valuable for the readers.<br><br>Authors’ response:<br>We added one sentence on page 4 (lines13-16) with related references:<br>“Indeed, data from recently published developmental transcriptomes of other Lophotrochazoans (e.g., Aplysia californica and Platynereis dumerilii) have highlighted insights into molecular mechanisms underlying early development and metamorphosis [6, 7].<br><br>Comment 3:

Powered by Editorial Manager® and ProduXion Manager® from Aries Systems Corporation

3)The study focuses on larval stages, up to the segmented larva. However, in the abstract the authors write that their resource will be of 'substantial value for understanding how gene repertoires are involved in early stages of development, ontogenic morphogenesis and the formation of an unsegmented body plan." Since no data are reported for later, unsegmented stages, the value of this resource to directly address that problem (i.e. how the unsegmented body forms from a segmented body) is limited. This sentence should be rewritten or data from unsegmented stages should also be included.
Authors' response:
As suggested by the reviewer #1, the sentence has been modified on page 2 (lines 4-7) as following:
"For this reason, transcriptome analyses from various developmental stages of Echiurid can be of substantial value for understanding the precise expression level and the complex regulatory networks during early and larval development."

Reviewer #2: This is an interesting article and a useful dataset, which will be the basis for future work both by the authors and related researchers. However, the text as it stands needs revision to correct some important points. When this is done, it will be a very handy addition to the published record for a variety of researchers.

Major points:
Comment 1:
-replicates have not been performed for timepoints. This should be clearly stated throughout as it is a major limitation of this study. Lines 44-46 of page 2 and lines 46 - 60 on page 9 have major issues, as without replicates these could be outliers, resulting from PCR, assembly or transcriptional artefacts or other problems. It should also be made clear to the reader that without replication, hypotheses of differential expression are tentative at best. Even just double checking these 12,910 "differentially expressed" contigs in particular to ensure that they are actual genes would be useful.

Authors' response:
We agree with the reviewer that biological replicates may contribute to minimizing the number of false positive genes and detecting these differentially expressed genes statistically. However, this limitation can't be fully overcome without the genome sequence. To point out this limitation, we added one sentence on page 10 (lines 15-18) as following:
"Although this study presents the first large-scale developmental transcriptome dataset for a developmentally interesting animal group, U. unicinctus (Echiurida), its global landscape of developmental transcriptome is not yet complete, due to the lack of biological replicates and reference genome sequences."

As already described in the manuscript on page 8 (lines 5-11), we applied several bioinformatics analysis methods to detect putative CDS as following:
"After assembly, open reading frames (ORFs) were predicted using TransDecoder (version 3.0.0) (http://transdecoder.sourceforge.net). To maximize sensitivity for capturing ORFs, all transcripts were aligned against the Uniprot/Swiss-Prot database (http://www.uniprot.org) via BLASTP search with an E-value cutoff of 10-5. Next, ORF length < 100 amino acids were discarded to avoid maintaining transcripts with poor evidence for protein-coding regions. Finally, redundant transcripts with more than 99% sequence identity were removed using CD-HIT (version 4.6.5)"

Comment 2:
-Genomic reads are not described or provided. These should be mentioned or cited, as at present Fig 3A is difficult to assess. It is also not directly relevant to a transcriptomic publication, as genome size is not necessarily reflective of transcriptomic complexity.

Authors' response:
Although we utilized genomic reads to estimate the genome size of U. unicinctus, we deleted these statements because this comment is worthy of consideration.

Comment 3:
-how many adult individuals were used to produce embryos? This will be important to assess allelic diversity.

Powered by Editorial Manager® and ProduXion Manager® from Aries Systems Corporation

Authors' response:
We modified the sentence on page 5 (lines 10-12) as following:
"We extracted eggs and sperm from one adult female and one male. To obtain U. unicinctus embryos, artificial fertilization was performed by mixing the appropriate ratio of sperms and eggs."

Comment 4:
- Particularly given the feeding of trochophore larvae, it is surprising that no assessment of potential contamination is performed or provided. Please address this, perhaps by performing a basic blastn against the non metazoan (or even just the algal and bacterial) section of the nr database for each timepoint and report the degree of any potential contamination (e.g. matching with high similarity) if it is found. If it is found, BUSCO results etc may need to be checked. It would be useful to also cross-reference the 12910 "differentially expressed" set against this.

Authors' response:
We excluded sequences derived from non-metazoan taxa and re-performed all further analyses including BUSCO, PCA, "differentially expressed genes".
Here is the updated version of text.
From Page 8 (line 21) to page 9 (lines 1-14): "To annotate coding sequences (CDS), the resulting 60,472 CDSs were compared against the NCBI non-redundant protein (NR) database (downloaded on April 11, 2017) using BLASTP with an E-value cutoff of 10-10 and the best blast hit. About 66 % (40,111/60,472) of the CDS were assigned to superphylum-level taxa including Lophotrochozoa (40%), Deuterostomia (8%), and Panarthropoda (2%) (Fig. 3A), which is to be generally expected. For further analysis, we excluded a number of CDSs (18%; 7,231/40,111) by using sequences derived from non-metazoan taxa. When there were multiple coding sequences that mapped to the same gene in the NR database, the sequences with the longest CDS were first assigned to that gene. Based on this criterion, we established a comprehensive transcriptome database of 14 early developmental stages of U. unicinctus that comprises 20,305 representative functional protein-coding transcripts. We further assessed the completeness of the U. unicinctus development transcriptome using the program BUSCO (bench-marking universal single-copy orthologs) (version 2.0) [14]. 75.9% (230 / 303 genes), and 75.7% (740 / 978 genes) of the eukaryote and metazoan single-copy orthologs were identified, respectively (Fig. 3B)"

and page 10 (lines 11-13): "We identified a total of 12,705 genes that showed dynamic regulation patterns during the differentiation and maturation of U. unicinctus cells (Fig. 4)."

Comment 5:
-Page 7, lines 1-15: estimates of coverage are misleading at best when cross-comparing genomic and transcriptomic data. Transcriptomes are almost certainly non-uniform in coverage. This section needs to be re-considered.

Authors' response:
Please see response to Comment 2.

Comment 6:
-Fig 2 seems to indicate that "quantifying expression level" (right column) was performed on the transcriptome build before removal of redundancy (left hand column). Is this correct? Or was the quantification performed on the CD-HIT - cured set of transcripts? (and quantifying expression level would then follow after CD-HIT, rather than being independent?)

Authors' response:
We performed the "abundance estimation" after removal of redundants. We redrew the Figure 2.

Minor points: (page numbers as per those 'burned in' at bottom of pages, not pdf page numbers, which have additional review pages)

Comment 7:
-Give an assembly download link in the text (and thanks for making these available for

Powered by Editorial Manager® and ProduXion Manager® from Aries Systems Corporation

	review – this was important)<br><br>Authors' response:<br>We added the BioProject link on page 11 (line 8) in the manuscript as following:<br>"(https://www.ncbi.nlm.nih.gov/bioproject/?term=PRJNA394029)"<br><br>All further data (will be updated because of "comment 4") have already deposited in the GigaDB, which probably can be provided from Gigascience journal. Here is the list of files we deposited.<br><br>1. Urechis_Dev_Draft_Trinity.fasta<br>(Draft de novo transcriptome assembly using Trinity)<br>2. A. Urechis_Dev_Removal_Redundant_mRNA.fasta<br>(mRNA sequences after gene prediction and removing redundant)<br>B. Urechis_Dev_Removal_Redundant_cds.fasta<br>(Coding sequences (nucleotide) after gene prediction and removing redundant)<br>C. Urechis_Dev_Removal_Redundant_prot.fasta<br>(Coding sequences (amino acid) sequences after gene prediction and removing redundant)<br>3. A. Urechis_Dev_CDS_M_mRNA.fasta<br>(mRNA sequences of representative protein coding sequence)<br>B. Urechis_Dev_CDS_M_cds.fasta<br>(Coding sequences (nucleotide) of representative protein coding sequence)<br><br>C. Urechis_Dev_CDS_M_prot.fasta<br>(Coding sequences (amino acid) of representative protein coding sequence)<br>D. Urechis_Dev_CDS_M.gff3<br>(Annotation of representative protein coding sequence)<br>4. Urechis_Dev_CDS_M_UPGMA_tree.nwk<br>(Dendrogram based on pairwise distance matrices (1-rho, Spearman's correlation coefficient)<br>5. Urechis_Dev_CDS_M_EXP.tsv<br>(Expression level (FPKM) of 14 stages)<br>6. A. Urechis_Dev_CDS_M_BUSCO_eukaryota.zip<br>(Results of BUSCO analysis using eukaryote single-copy orthologs)<br>B. Urechis_Dev_CDS_M_BUSCO_metazoa.zip<br>(Results of BUSCO analysis using metazoan single-copy orthologs)<br><br>Comment 8:<br>pg 2 line 13 - should this be "of echiurid species" (echiurid as an adjective, not a noun?)<br><br>Authors' response:<br>Corrected.<br><br>Comment 9:<br>pg 2 line 26: average - is this the mean, median? N50 useful here?<br><br>Authors' response:<br>The meaning of "average" in our manuscript indicates "mean", but the use of N50 is better than "average length". We updated this section on page 2 (lines 8-10) as following:<br>"A total of 914 million raw RNA-Seq reads were produced from 14 developmental stages of Urechis unicinctus, and were de novo assembled into contigs spanning 63,928,225 bp with an N50 length of 2,700 bp."<br><br>Comment 10:<br>pg 5 line 13 - "sandy bottom" is an odd phrasing. "Sandy intertidal areas?"<br><br>Authors' response:<br>It was changed into "intertidal mud flat" on page 5 (line 9).

Powered by Editorial Manager® and ProduXion Manager® from Aries Systems Corporation

Comment 11:
-Can any developmental landmarks be given for the early/mid/late trochophore stage, or refer tophotographs?

Authors’ response:
The following sentences and reference on page 5 (lines 20-23) and page 6 (lines 1-2) added into the revised manuscript.
“Diagnostic features for each of the three trochophore stages are as follows. The early trochophore is a non-feeding stage. In the middle trochophore, the gastro-intestinal valve opens and the anus appears. Late trochophore has longer cilia of the apical tufts that are replaced by shorter cilia which cover a greater area and the prototroch cilia are longer. The developmental stages of the U. unicinctus embryos followed Newby 's classification [8].”

Comment 12:
-What artificial seawater was used?

Authors’ response:
We added the sentence on page 5 (lines 13-14) as following:
“The embryos were reared in artificial seawater (Reef crystals (Aquarium Systems, France)) in a plastic case at room temperature (18-20 °C).”

Comment 13:
pg 6 line 10 mRNA (not the mRNAs)

Authors’ response:
Corrected.

Comment 14:
pg 6 line 20 how many rounds of PCR in library construction? What adapters used?

Authors’ response:
We added and modified a couple of sentences on page 6 (lines 12-22) and page 7 (lines 1-7) as following:
“Total RNA concentration was calculated by Quant-IT RiboGreen (Invitrogen, #R11490). To assess the integrity of the total RNA, samples are run on the TapeStation RNA screentape (Agilent, #5067-5576). Only high-quality RNA preparations, with RIN greater than 7.0, were used for RNA library construction. A library was independently prepared with 1ugg of total RNA for each sample by Illumina TruSeq Stranded Total RNA Sample Prep Kit (Illumina, Inc., San Diego, CA, USA). The rRNA in total RNA is depleted by Ribo-Zero kit. After the rRNA is depleted, the remaining RNA is purified, fragmented and primed for cDNA synthesis. The cleaved RNA fragments are copied into first strand cDNA using reverse transcriptase and random hexamers. This is followed by second strand cDNA synthesis using DNA Polymerase I, RNase H and dUTP. These cDNA fragments then go through an end repair process, the addition of a single ‘A’ base, and then ligation of the adapters. The products are then purified and enriched with PCR to create the final cDNA library. The libraries were quantified using qPCR according to the qPCR Quantification Protocol Guide (KAPA Library Quantification kits for Illumina Sequencing platforms) and qualified using the TapeStation D1000 ScreenTape (Agilent Technologies, Waldbronn, Germany). Indexed libraries were then submitted to a Illumina Hiseq 4000 (Illumina, Inc., San Diego, CA, USA), and the paired-end (2×100 bp) sequencing was performed.”

Comment 15:
pg 8 line 22, and Fig 3B. Is this best blast hits, please report this in the figure legend and text
if so.

Authors’ response:
Yes, it is the best blast hit. We updated this section and figure legend on (1) page 8 (lines 21-22), page 9 (line 1), and (2) page 15 (lines 6-7) as following:
(1) “To annotate coding sequences (CDS), the resulting 60,472 CDSs were compared against the NCBI non-redundant protein (NR) database (downloaded on April 11,

2017) using BLASTP with an E-value cutoff of 10-10 and the best blast hit."
(2) "Superphylum distribution for homology search of U. unicinctus coding sequences against the NR database using the best blast hit."

Comment 16:
Page 8 lines 48 - 53: This seems a deeply flawed comparison, and as you acknowledge, compares very non-comparable things. Maybe find recent transcriptomes that have used BUSCO as an assessment tool and use these as exemplars? All of these were published before BUSCO.

Authors' response:
Without a reference genome, highly accurate whole transcriptome assembly from short reads will be a tough challenge. So far, there have been no reports of the completeness assessment of the de novo transcriptome assemblies using the BUSCO scores. Therefore, we deleted these sentences in our revised manuscript.

Comment 17:
-page 9 line 47: 10 fold (total)? or is this a log2(a/b) measure? The FDR also seems to be
communicated oddly. Do you mean a p-value cutoff for FDR of 0.001?

Authors' response:
The fold value is 10-fold, and the score 0.001 is for FDR cut-off but not for p-value. To clarify and more fully explain its meaning, we modified the sentence on page 10 (lines 9-11) as following:
"In an additional analysis, a gene whose expression level was significantly changed (10-fold and FDR adjusted P value 0.1%) in at least one comparison, was defined as a developmentally regulated gene."

Comment 18:
Pg 13 line 5: I think it better to say "Adult Urechis unicinctus" (no "worm of)

Authors' response:
Corrected.

Comment 19:
Line 44: scale spelled "sclae"

Authors' response:
Corrected.

Comment 20:
In several locations and in data - I understand the use of FKPM, but TPM may be a more useful measure - it allows the cross - comparison of these results between samples, while FKPM does not.Consider providing this information if you have it.

Authors' response:
We agree, and investigated whether there is difference in expression profiles based on between TPM and FPKM values. In all 14 samples, the expression level values between TPM and FPKM are highly coupled (see the below table), indicating that our results could not be influenced by gene expression measurements.

P<2.2e-16 with all comparisons

	TPM vs FPKM
stage	Spearman'ss (p)
oocytes	0.9998206
fertilized egg	0.9998886
polar body	0.9999137
2 cell	0.9998949
4 cell	0.9998529
8 cell	0.9999356
16 cell	0.9998927
32 cell	0.999884
blastular	0.9999695

Powered by Editorial Manager® and ProduXion Manager® from Aries Systems Corporation

emerged cilia 0.9999853
early trochophore 0.999974
middle trochophore 0.9999774
late trochophore 0.9999885
segmentation 0.9999447

Comment 21:
- the references are in an odd font, which may require changing depending on journal preferences.

Authors' response:
Corrected.

Reviewer #3:
Comment 1:
The paper presents transcriptomic libraries for 14 developmental stages of an echiurid species. This is within the scope of the journal. Hence, I suggest accepting the paper after a major revision. My major concern is the introduction. The introduction does not adequately reflect the literature on the position of Echiura. With respect to morphological data the studies of Hessling concerning the nervous system are missing. This among others also reflected in the erroneous statement that the presence of a larval nervous system includes placement in Annelida. However, this is in this way not correct. Larval nervous systems occur throughout the animal kingdom, it is the specific features of this system, which support this placement and require citing the works by Hessling. Moreover, recent studies using phylogenomic data concerning the phylogeny of Annelida are also not mentioned (see below). Finally, for understanding the early evolution of Lophotrochozoa Echiura are irrelevant as they are highly derived annelid taxa. Echiura are more interesting in understanding the plasticity of body plan evolution in general, but this independent of their position within Lophotrochozoa. Moreover, citations supporting the claim that Urechis unicinctus attracted special attention for evo-devo are lacking and should be provided.

Authors' response:
We appreciate the reviewer's feedback and agree with the suggested changes. In the introduction, we have now corrected the incorrect statement about the larval nervous system and have added references to Hessling's work. We have also added references to the recent phylogenomic research on the placement of Echiurids within Annelida. We also have changed the sentence about evo-devo to emphasize instead the potential use of Urechis to understand the evolutionary loss of adult segmentation. Finally, we made all corrections according to your minor comments (see all changes below).
Here is the updated text on page 4 (lines 3-21):
"Within the major annelid groups, Echiurida (also called the 'marine spoon worms') is represented by a morphologically and ontogenetically unique assemblage that includes approximately 165 species, most of which lacks segmentation in adults, although they possess annelid-like morphological and developmental features including the organization of the larval nervous system [1]. They were once considered a separate metazoan phylum, but reevaluation of morphological and molecular data indicated that Echiurida is nested within the Annelida representing one of three animal phyla with body segmentation [1-5]. In this respect, transcriptome analyses from various developmental stages of Echiurida are of substantial value for understanding precise expression levels and the complex regulatory networks involved in early and larval development. Indeed, data from recently published developmental transcriptomes of other Lophotrochazoans (e.g., Aplysia californica and Platynereis dumerilii) have highlighted insights into molecular mechanisms underlying early development and metamorphosis [6, 7].
Urechis unicinctus is an Echiuran species that inhabits burrows in intertidal soft sediments (Fig. 1). The Urechis genus may hold important clues to the genetic basis of the evolutionary gain and loss of segmentation, due to its nested position within Annelida (i.e., sister to capitellid polychaetes), a Lophotrochozoan phylum that is represented by a diverse group of segmented worms [2, 5]."

Reviewer #3's minor comments:

Comment 2:
Page 2, Line 6: delete "the phylum"

Authors' response:
Authors want to keep "the phylum" in its original place.

Comment 3:
Page 2, Line 8: change to "unlike most other annelid"

Authors' response:
changed

Comment 4:
Page 2, Line 16: insert space between "development, ontogenetic"

Authors' response:
corrected

Comment 5:
Page 2, Line 57: insert dot between "stages. These"

Authors' response:
corrected

Comment 6:
Page 4, Line 36: delete dot between "(Fig. 1). This"

Authors' response:
modified

Comment 7:
Page 4, Line 48: delete "the" at the end of the line

Authors' response:
deleted

Comment 8:
Page 4, Line 51: "profiles" instead of "profiling"

Authors' response:
changed

Comment 9:
Page 7, Line 2: describe how the k-mer analysis was conducted in detail

Authors' response:
We deleted this section because review #1 (comment 1; "The authors seem to estimate the genome size of Urechis unicinctus based on k-mer analysis. However, for this they would need to use genomic reads, and not transcriptome reads. This estimate should be deleted, unless it was done on genomic sequences, in which case more detail should be given") and reviewer #2 (comment 2; "It is also not directly relevant to a transcriptomic publication, as genome size is not necessarily reflective of transcriptomic complexity") also pointed out same issue.

Comment 10:
Page 7, Lines 18-25: I do not understand what has been done here. Concatenation means that individual sequences are attached to each other one after the other, but I think this is not meant here. Rather assume that the reads from all 14 libraries were pooled together for assembly of the data. Please clarify this point.

Authors' response:
We modified "concatenated" into "pooled".

Comment 11:

	Page 8, Line 7: How did you correct the expression profiles for the uneven sequencing depth among the different libraries? The range is from 43 million reads to 140 million and hence the latter is more than three times higher.<br><br>Authors' response:<br>The FPKM value we used in our analysis is quantifying RNA-seq data by normalizing (1) read length and (2) the number of reads sequenced. According to the latter case, the expression profiles among libraries with uneven sequencing depth have been already normalized in order to compare expression levels of genes among these libraries.<br><br>Comment 12:<br>Page 8, Line 46: include a comma in "were identified,"<br><br>Authors' response:<br>corrected<br><br>Comment 13:<br>Page 13, Line 24: This is not a phylogenetic tree, which by definition can only be reconstructed between species and not developmental stages between individuals. Change wording.<br><br>Authors' response:<br>This sentence on page 15 (lines 8-10) was changed as following:<br>"Result of principal component analysis (PCA) and a dendrogram of transcriptomes of 14 U. unicinctus developmental stages based on pairwise distance matrices (1 - , Spearman's correlation coefficient)."<br><br>Comment 14:<br>Page 13, Line 44: "mouth" instead of "mouse"<br><br>Authors' response:<br>corrected<br><br>Comment 15:<br>Page 13, Line 44: insert a space between "50μm. (B)"<br><br>Authors' response:<br>a space is inserted<br><br>Comment 16:<br>Citations missing:<br>Hessling, R. & Westheide, W. (2002) Are Echiura derived from a segmented ancestor? Immunohistochemical analysis of the nervous system in developmental stages of Bonellia viridis.<br>Journal of Morphology, 252, 100-113.<br>Hessling, R. (2002) Metameric organisation of the nervous system in developmental stages of Urechis<br>caupo (Echiura) and its phylogenetic implications. Zoomorphology, 121, 221-234.<br>Hessling, R. (2003) Novel aspects of the nervous system of Bonellia viridis (Echiura) revealed by<br>the combination of immunohistochemistry, confocal laser-scanning microscopy and three-dimensional<br>reconstruction. Hydrobiologia, 496, 225-239.<br>Struck, T.H., Paul, C., Hill, N., Hartmann, S., Hösel, C., Kube, M., Lieb, B., Meyer, A., Tiedemann, R., Purschke, G. & Bleidorn, C. (2011) Phylogenomic analyses unravel annelid evolution.<br>Nature, 471, 95-98.<br>Weigert, A., Helm, C., Meyer, M., Nickel, B., Arendt, D., Hausdorf, B., Santos, S.R., Halanych,<br>K.M., Purschke, G., Bleidorn, C. & Struck, T.H. (2014) Illuminating the base of the annelid tree<br>using transcriptomics. Molecular Biology and Evolution, 31, 1391-1401.<br>Struck, T.H., Golombek, A., Weigert, A., Franke, Franziska A., Westheide, W.,

Powered by Editorial Manager® and ProduXion Manager® from Aries Systems Corporation

	Purschke, G.,<br>Bleidorn, C. & Halanych, K.M. (2015) The Evolution of Annelids Reveals Two Adaptive Routes to the<br>Interstitial Realm. Current Biology, 25, 1993-1999.<br>Andrade, S.C.S., Novo, M., Kawauchi, G.Y., Worsaae, K., Pleijel, F., Giribet, G. & Rouse, G.W.<br>(2015) Articulating "archiannelids": Phylogenomics and annelid relationships, with emphasis on<br>meiofaunal taxa. Molecular Biology and Evolution.<br><br>Authors' response:<br>Added.
**Additional Information:**	
**Question**	**Response**
Are you submitting this manuscript to a special series or article collection?	No
**Experimental design and statistics**<br><br>Full details of the experimental design and statistical methods used should be given in the Methods section, as detailed in our Minimum Standards Reporting Checklist. Information essential to interpreting the data presented should be made available in the figure legends.<br><br>Have you included all the information requested in your manuscript?	Yes
**Resources**<br><br>A description of all resources used, including antibodies, cell lines, animals and software tools, with enough information to allow them to be uniquely identified, should be included in the Methods section. Authors are strongly encouraged to cite Research Resource Identifiers (RRIDs) for antibodies, model organisms and tools, where possible.<br><br>Have you included the information requested as detailed in our Minimum Standards Reporting Checklist?	Yes
**Availability of data and materials**<br><br>All datasets and code on which the conclusions of the paper rely must be either included in your submission or deposited in publicly available repositories (where available and ethically	Yes

Powered by Editorial Manager® and ProduXion Manager® from Aries Systems Corporation

appropriate), referencing such data using a unique identifier in the references and in the "Availability of Data and Materials" section of your manuscript.<br><br>Have you have met the above requirement as detailed in our Minimum Standards Reporting Checklist?	

Manuscript Click here to download Manuscript Han et al-Revised MS.docx

Data Note

The developmental transcriptome atlas of the spoon worm *Urechis unicinctus* (Echiurida: Annelida)

Yong-Hee Han[1#], Sung-Gwon Lee[2#], Kyoung-Bin Ryu[1#], Jooseong Oh[2], Elizabeth M. A. Kern[3], Joong-Ki Park[3*], Chungoo Park[2*], Sung-Jin Cho[1*]

[1]School of Biological Sciences, College of Natural Sciences, Chungbuk National University, Cheongju, Chungbuk 28644, Republic of Korea

[2]School of Biological Sciences and Technology, Chonnam National University, Gwangju 61186, Republic of Korea

[3]Division of EcoScience, Ewha Womans University, Seoul 03760, Republic of Korea

[#] These authors contributed equally to this work.

*Corresponding Authors.

E-mail addresses:

Sung-Jin Cho, sjchobio@chungbuk.ac.kr. Tel: +82-43-261-2294. Fax: +82-43-260-2298.

Chungoo Park, chungoo@jnu.ac.kr. Tel: +82-62-530-1913. Fax: +82-62-530-2199

Joong-Ki Park, jkpark@ewha.ac.kr. Tel: +82-2-3277-5948. Fax: +82-2-3277-2385.

Abstract

Background: Echiurida is one of the most intriguing major subgroups of the phylum Annelida, because unlike most other annelids, echiurids lack metameric body segmentation as adults. For this reason, transcriptome analyses from various developmental stages of Echiurid can be of substantial value for understanding precise expression levels and the complex regulatory networks during early and larval development.

Finding: A total of 914 million raw RNA-Seq reads were produced from 14 developmental stages of *Urechis unicinctus*, and were *de novo* assembled into contigs spanning 63,928,225 bp with an N50 length of 2,700 bp. The resulting comprehensive transcriptome database of the early developmental stages of *U. unicinctus* consists of 20,305 representative functional protein-coding transcripts. Approximately 66 % of unigenes were assigned to superphylum-level taxa, including Lophotrochozoa (40%). The completeness of the transcriptome assembly was assessed using BUSCO, and 75.7 % of the metazoan single-copy orthologs were presented in our transcriptome database. We observed three distinct patterns of global transcriptome profiles from 14 developmental stages, and identified a total of 12,705 genes that showed dynamic regulation patterns during the differentiation and maturation of *U. unicinctus* cells.

Conclusions: We present the first large-scale developmental transcriptome dataset of *U. unicinctus* and provide a general overview of the dynamics of global gene expression changes during its early developmental stages. The analysis of time-course gene expression data is a first step toward understanding the complex developmental gene

regulatory networks in *U. unicinctus*, and will furnish a valuable resource for analyzing the functions of gene repertoires in various developmental phases.

Keywords: *Urechis unicinctus*, Echiurida, Developmental transcriptome, RNA-Seq, *de novo* assembly

Data Description

Background

Within the major annelid groups, Echiurida (also called the ‘marine spoon worms’) is represented by a morphologically and ontogenetically unique assemblage that includes approximately 165 species, most of which lack segmentation as adults, although they possess annelid-like morphological and developmental features including the organization of the larval nervous system [1]. They were once considered a separate metazoan phylum, but reevaluation of morphological and molecular data indicated that Echiurida is nested within the Annelida, which represents one of the three animal phyla with body segmentation [1-5]. In this respect, transcriptome analyses from various developmental stages of Echiurida are of substantial value for understanding precise expression levels and the complex regulatory networks involved in early and larval development. Indeed, data from recently published developmental transcriptomes of other Lophotrochazoans (e.g., *Aplysia californica* and *Platynereis dumerilii*) have highlighted insights into molecular mechanisms underlying early development and metamorphosis [6, 7].

Urechis unicinctus is an echiuran species that inhabits burrows in intertidal soft sediments (Fig. 1). The *Urechis* genus may hold important clues to the genetic basis of the evolutionary gain and loss of segmentation, due to its nested position within Annelida (i.e., sister to capitellid polychaetes), a Lophotrochozoan phylum that is represented by a diverse group of segmented worms [2, 5]. However, current knowledge is limited on the molecular mechanisms that underlie the ontogeny of *U. unicinctus*. The goal of this study is to enhance our understanding of gene expression during embryonic

development. Here we report the transcriptome profiles (generated with the Illumina HiSeq platform) of developing embryos of *U. unicinctus*. Transcriptome sequencing data assist in the discovery of the roles of genes involved in various embryological and larval development processes. As the first large-scale transcriptomic dataset for *U. unicinctus,* this resource will help in the validation of development-specific gene features predicted by the genome.

Sample collection, embryo culture, and RNA isolation

Adults of *U. unicinctus* were collected from intertidal mud flats on the southern coast of South Korea. We extracted eggs and sperm from one adult female and one male. To obtain *U. unicinctus* embryos, artificial fertilization was performed by mixing the appropriate ratio of sperms and eggs.

Embryos were reared in artificial seawater (Reef crystals (Aquarium Systems, France)) in a plastic case at room temperature (18-20 °C). The late trochophore, a typical larval stage in which the intestinal tract is formed, was fed with a microalgae called *Isochrysis galbana*. Reared embryo samples were collected at each of the following stages: 0 h (unfertilized egg), 0.5 h post fertilization (fertilized egg), polarbody cell, 2 cell, 4 cell, 8 cell, 16 cell, 32 cell, blastula, emerged cilia, early trochophore (day 1), middle trochophore (day 2), late trochophore (day 5), and segmentation stage (day 30~45). Diagnostic features for each of the three trochophore stages are as follows. The early trochophore is a non-feeding stage. In the middle trochophore, the gastro-intestinal valve opens and the anus appears. In the late trochophore, the longer cilia of the apical tufts are replaced by shorter cilia which cover

a greater area, and the prototroch cilia are longer. These developmental stages follow Newby's classification [8].

Total RNA was isolated from the embryos of the above samples using TRIZOL reagent (Invitrogen, Carlsbad, CA, USA) following the manufacturer's instructions. The purity and integrity of the total RNA isolated from each embryo sample were examined using a Nanodrop 2000C spectrophotometer (Thermo Scientific, Waltham, MA, USA) and Bioanalyzer 2100 (Agilent Technologies, Palo Alto CA, USA). Adult images were taken on a Canon EOS 550D, and embryo bright-field images were taken on a Leica DM6 B microscope using DIC optics.

TruSeq Stranded Ribo-Zero library preparation and sequencing

Total RNA concentration was calculated by Quant-IT RiboGreen (Invitrogen, #R11490). To assess the integrity of the total RNA, samples were run on TapeStation RNA screentape (Agilent, #5067-5576). Only high-quality RNA preparations, with RIN greater than 7.0, were used for RNA library construction. A library was independently prepared with 1μg of total RNA for each sample by Illumina TruSeq Stranded Total RNA Sample Prep Kit (Illumina, Inc., San Diego, CA, USA). The rRNA in total RNA is depleted by the Ribo-Zero kit. After the rRNA was depleted, the remaining RNA was purified, fragmented and primed for cDNA synthesis. The cleaved RNA fragments were copied into first strand cDNA using reverse transcriptase and random hexamers. This was followed by second strand cDNA synthesis using DNA Polymerase I, RNase H and dUTP. These cDNA fragments then underwent an end repair process, the addition of a

single ‘A’ base, and ligation of the adapters. The products were then purified and enriched with PCR to create the final cDNA library. The libraries were quantified using qPCR according to the qPCR Quantification Protocol Guide (KAPA Library Quantification kits for Illumina Sequencing platforms) and qualified using the TapeStation D1000 ScreenTape (Agilent Technologies, Waldbronn, Germany). Indexed libraries were then submitted to a Illumina Hiseq 4000 (Illumina, Inc., San Diego, CA, USA), and paired-end (2×100 bp) sequencing was performed. The resulting samples were sequenced on the Illumina HiSeq 2000 system with a paired-end read with 101 cycles or the Illumina HiSeq 4000 system with a paired-end read with 151 cycles (Table 1). The experimental procedures and complete assembly pipeline are summarized in Fig. 2.

Transcriptome preprocessing and *de novo* assembly

After completion of the sequencing run, to obtain high-quality clean reads from the raw data (i.e., removing those containing adapter sequences, poly-N sequences, or low quality bases), we performed quality-based trimming and filtering using Trimmomatic (version 0.33) [9] with the parameters ILLUMINACLIP:TruSeq3-PE-2.fa:2:30:10 LEADING:3 TRAILING:3 SLIDINGWINDOW:4:15 MINLEN:36 for the 101 bp library (or MINLEN:50 for the 151 bp library). An average of 63 million clean reads per sample was obtained (Table 1).

Before *de novo* assembly, all clean reads were pooled without normalization of read abundance, even though the use of all merged reads may require progressively

increasing assembly time and memory usage, in order to obtain a comprehensive reference transcriptome database. The merged reads were used for *de novo* transcriptome assembly using Trinity (version 2.1.1) [10] with default parameters. The resulting assembled transcriptome consisted of 620,490 transcripts with an N50 value of 846 bp (Table 2). After assembly, open reading frames (ORFs) were predicted using TransDecoder (version 3.0.0) (http://transdecoder.sourceforge.net). To maximize sensitivity for capturing ORFs, all transcripts were aligned against the Uniprot/Swiss-Prot database (http://www.uniprot.org) via BLASTP search with an *E*-value cutoff of 10^{-5}. Next, ORF length < 100 amino acids were discarded to avoid maintaining transcripts with poor evidence for protein-coding regions. Finally, redundant transcripts with more than 99% sequence identity were removed using CD-HIT (version 4.6.5) [11], producing a total of 60,472 non-redundant ORFs. These sequences span 63,928,225 bp with an N50 length of 2,700 bp.

To quantify expression levels, the reads for each library were mapped independently to the reference *U. unicinctus* transcriptome sequences using Bowtie (version 2.2.6) [12], and expression levels of these transcripts were estimated with RSEM (version 1.2.26) [13]. The unit of expression level is referred to as fragment per kilobase of transcript per million fragments mapped (FPKM) in our analyses.

Annotation

To annotate coding sequences (CDS), the resulting 60,472 CDSs were compared against the NCBI non-redundant protein (NR) database (downloaded on April

11, 2017) using BLASTP with an *E*-value cutoff of 10^{-10} and the best BLAST hit. About 66 % (40,111/60,472) of the CDS were assigned to superphylum-level taxa including Lophotrochozoa (40%), Deuterostomia (8%), and Panarthropoda (2%) (Fig. 3A), which is to be generally expected. For further analysis, we excluded a number of CDSs (18%; 7,231/40,111) by using sequences derived from non-metazoan taxa. When there were multiple coding sequences that mapped to the same gene in the NR database, the sequences with the longest CDS were first assigned to that gene. Based on this criterion, we established a comprehensive transcriptome database of 14 early developmental stages of *U. unicinctus* that comprises 20,305 representative functional protein-coding transcripts. We further assessed the completeness of the *U. unicinctus* development transcriptome using the program BUSCO (bench-marking universal single-copy orthologs) (version 2.0) [14]. 75.9% (230 / 303 genes), and 75.7% (740 / 978 genes) of the eukaryote and metazoan single-copy orthologs were identified, respectively (Fig. 3B).

Transcriptome comparisons

To show that gene expression reflects development-specific differentiation and maturation processes, we built expression distance matrices for each developmental stage and constructed a gene expression tree (Fig. 3C). Two major transitions in expression patterns were observed: (1) blastula to emerged cilia and (2) late trochophore to segmentation. These transitions divided the 14 *U. unicinctus* developmental stages into three phases: the oocyte, polar body, fertilized, 2-, 4-, 8-, 16-, 32-cell embryo, and

blastula stages make up Phase I; the emerged cilia, early-, middle-, and late-trochophore stages compose Phase II; and the segmentation stage makes up to Phase III. These three distinct phases of global transcriptome profiles covering 14 developmental stages were supported by principal component analysis (PCA), which was performed using the "prcomp" function in the "stats" package in R (version 3.2.4) (Fig. 3C). These results suggest that developmental stages are well characterized by our transcription profiles, and the differential gene expression profiles presented in this study will be useful for further study of ontogenic processes at the gene expression level.

In an additional analysis, a gene whose expression level was significantly changed ($\geq$ 10-fold and FDR adjusted *P* value $\leq$ 0.1%) in at least one comparison was defined as a developmentally regulated gene. We identified a total of 12,705 genes that showed dynamic regulation patterns during the differentiation and maturation of *U. unicinctus* cells (Fig. 4). Note that we used the TMM (trimmed mean of M values) normalization [15] provided by edgeR bioconductor package for R for this test.

Although this study presents the first large-scale developmental transcriptome dataset for a developmentally interesting animal group, *U. unicinctus* (Echiurida), its global landscape of developmental transcriptome is not yet complete, due to the lack of biological replicates and reference genome sequences.

In summary, we present the first large-scale, developmental stage-specific transcriptome dataset for *U. unicinctus*, and provide a general overview of the dynamics of global gene expression changes at different developmental stages. These data will fill an important gap in phylum-wide comparisons of gene expression patterns, and will

lead to a better understanding of gene repertoires involved in different developmental stages and of complex developmental gene regulatory networks.

Availability of supporting data

All raw sequencing data used for assembly have been deposited in the NCBI database under the accession numbers SRX2999418 to SRX2999431, associated with BioProject PRJNA394029 (https://www.ncbi.nlm.nih.gov/bioproject/?term=PRJNA394029). An additional dataset further supporting the results of this article can be found in the *GigaScienc*e repository, GigaDB.

Abbreviation

bp: base pairs; BUSCO: Bench-marking universal single-copy orthologs; CDS: Coding sequence; FDR: False discovery rate; FPKM: Fragments per kilobase of transcript per million mapped reads; Gb: Gigabases; ORFs: Open reading frames; PCA: Principal components analysis; RNA-Seq: High-throughput messenger RNA sequencing; TMM: Trimmed mean of M values.

Competing interests

The authors declare that they have no competing interests.

Authors' contribution

CP and SJC designed the study; JKP contributed to the project coordination; YHH, KBR, and SJC performed the experiments; SGL, JO, and CP analyzed the data and evaluated the conclusions; CP, SJC, JKP, SGL and EMAK wrote the paper; All authors read and approved the final manuscript.

Acknowledgements

This research was supported by a grant from the Collaborative Genome Program (20140428) funded by the Ministry of Oceans and Fisheries, Korea to CP, SJC, JKP. This research was supported by Basic Science Research Program through the National Research Foundation of Korea (NRF) funded by the Ministry of Education (NRF-2016R1D1A1B03933412).

Author details

[1]School of Biological Sciences, College of Natural Sciences, Chungbuk National University, Cheongju, Chungbuk 28644, Republic of Korea. [2]School of Biological Sciences and Technology, Chonnam National University, Gwangju 61186, Republic of Korea. [3]Division of EcoScience, Ewha Womans University, Seoul 03760, Republic of Korea

References

1. Struck TH, Schult N, Kusen T, Hickman E, Bleidorn C, McHugh D, et al. Annelid phylogeny and the status of Sipuncula and Echiura. Bmc Evolutionary Biology. 2007;7 doi: 10.1186/1471-2148-7-57.
2. Zrzavý J, Říha P, Piálek L and Janouškovec J. Phylogeny of Annelida (Lophotrochozoa): total-evidence analysis of morphology and six genes. BMC Evolutionary Biology. 2009;9 1:189. doi:10.1186/1471-2148-9-189.
3. Struck TH, Paul C, Hill N, Hartmann S, Hosel C, Kube M, et al. Phylogenomic analyses unravel annelid evolution. Nature. 2011;471 7336:95-8. doi:10.1038/nature09864.
4. Andrade SCS, Novo M, Kawauchi GY, Worsaae K, Pleijel F, Giribet G, et al. Articulating "Archiannelids": Phylogenomics and Annelid Relationships, with Emphasis on Meiofaunal Taxa. Molecular Biology and Evolution. 2015;32 11:2860-75. doi:10.1093/molbev/msv157.
5. Anne Weigert and Bleidorn C. Current status of annelid phylogeny. Organisms Diversity & Evolution. 2016;16 2:345-62. doi:10.1007/s13127-016-0265-7.
6. Heyland A, Vue Z, Voolstra CR, Medina M and Moroz LL. Developmental transcriptome of Aplysia californica'. Journal of Experimental Zoology Part B: Molecular and Developmental Evolution. 2011;316B 2:113-34. doi:10.1002/jez.b.21383.
7. Chou H-C, Pruitt MM, Bastin BR and Schneider SQ. A transcriptional blueprint for a spiral-cleaving embryo. BMC Genomics. 2016;17 1:552. doi:10.1186/s12864-016-2860-6.
8. Newby WW. The embryology of the echiuroid worm, Urechis caupo. Philadelphia,: The American Philosophical Society; 1940.
9. Bolger AM, Lohse M and Usadel B. Trimmomatic: a flexible trimmer for Illumina sequence data. Bioinformatics. 2014;30 15:2114-20. doi:10.1093/bioinformatics/btu170.
10. Grabherr MG, Haas BJ, Yassour M, Levin JZ, Thompson DA, Amit I, et al. Full-length transcriptome assembly from RNA-Seq data without a reference genome. Nat Biotech. 2011;29 7:644-52. doi:10.1038/nbt.1883.
11. Fu L, Niu B, Zhu Z, Wu S and Li W. CD-HIT: accelerated for clustering the next-generation sequencing data. Bioinformatics. 2012;28 23:3150-2. doi:10.1093/bioinformatics/bts565.
12. Langmead B, Trapnell C, Pop M and Salzberg SL. Ultrafast and memory-efficient alignment of short DNA sequences to the human genome. Genome Biology. 2009;10 3:R25. doi:10.1186/gb-2009-10-3-r25.
13. Li B and Dewey CN. RSEM: accurate transcript quantification from RNA-Seq data with or without a reference genome. BMC Bioinformatics. 2011;12 1:323. doi:10.1186/1471-2105-12-323.

14. Simão FA, Waterhouse RM, Ioannidis P, Kriventseva EV and Zdobnov EM. BUSCO: assessing genome assembly and annotation completeness with single-copy orthologs. Bioinformatics. 2015;31 19:3210-2. doi:10.1093/bioinformatics/btv351.

15. Robinson MD and Oshlack A. A scaling normalization method for differential expression analysis of RNA-seq data. Genome Biology. 2010;11 3:R25. doi:10.1186/gb-2010-11-3-r25.

Figure Legends

Figure 1. Adult *Urechis unicinctus* used in this study (proboscis retracted). Scale bar; 1cm.

Figure 2. Schematic diagram of *U. unicinctus* transcriptome analysis in this study.

Figure 3. Analysis of *de novo* transcriptome and global gene expression patterns.

(A) Superphylum distribution for homology search of *U. unicinctus* coding sequences against the NR database using the best BLAST hit. (**B)** Results of BUSCO analysis. (**C)** Result of principal component analysis (PCA) and a dendrogram of transcriptomes of 14 *U. unicinctus* developmental stages based on pairwise distance matrices (1 - ρ, Spearman's correlation coefficient). The first, second, and third principal components account for 86.8, 6.8, and 5.9 % of variance, respectively.

Figure 4. Representative images of *U. unicinctus* developmental stages and their gene expression profiles.

(A) Overview of *U. unicinctus* developmental stages. (a) oocyte, (b) fertilized embryo, (c) polar body, (d) 2 cell, (e) 4 cell, (f) 8 cell, (g) 16 cell, (h) 32 cell, (i) blastula, (j) emerged cilia, (k) early trochophore, (l) middle trochophore, (m) late trochophore, (n) segmentation. p, polar body; bp, blastopore; c, cilia; ls, larval stomach; int, intestine; glv, gastro-intestinal valve; m, mouth; vnc, ventral nerve cord; a, anus. Scale bar; 50μm. (**B)** A heat map showing dynamic gene expression patterns with the relative expression levels (column) in each stage (row). Expression values (TMM) were $\log_2$-transformed and mean-centered by transcript. The hierarchical clustering was performed with

Euclidean distances of gene expression values.

Click here to download Table Han et al-Table 1.docx

Table 1. Reads Statistics

Samples	Total produced bases (bp)	Number of reads	Read length (bp)	GC %	Q30 %	Number of clean reads (%)
Oocyte	8,749,299,078	57,942,378	151	43.87	90.53	54,583,372 (94.20)
Fertilized embryo	7,204,375,496	47,711,096	151	43.86	92.32	45,817,358 (96.04)
Polar body	7,553,516,790	50,023,290	151	41.40	91.12	47,401,970 (94.76)
2 cell	8,663,957,200	57,377,200	151	40.21	92.63	55,263,572 (96.32)
4 cell	6,693,881,642	44,330,342	151	40.88	90.81	43,001,172 (97.00)
8 cell	7,417,271,000	49,121,000	151	42.14	92.31	46,360,492 (94.38)
16 cell	7,993,095,608	52,934,408	151	41.52	91.75	50,571,562 (95.54)
32 cell	22,163,185,664	146,776,064	151	42.11	91.44	139,587,140 (95.10)
Blastula	8,885,042,038	58,841,338	151	45.23	92.04	56,298,300 (95.68)
Emerged cilia	8,077,246,398	53,491,698	151	44.18	89.83	50,401,516 (94.22)
Early trochophore	7,354,720,616	72,819,016	101	45.90	96.02	72,513,798 (99.58)
Middle trochophore	7,581,052,122	75,059,922	101	46.58	96.31	74,755,084 (99.59)
Late trochophore	7,807,192,940	77,298,940	101	46.69	96.66	77,100,204 (99.74)
Segmentation	10,556,984,102	69,913,802	151	48.19	92.37	67,990,654 (97.25)

 Click here to download Table Han et al-Revised Table 2.docx

Table 2. Statistics for *Urechis unicinctus* transcriptome assembly

Samples	Total assembled bases (bp)	Number of assembled transcripts	N50 transcript length (bp) (min - max : median)	Number of non-redundant ORFs	Number of ORFs with NR blast hit (longest ORF per unigene)
Oocyte	45,868,755	26,569	2,801 (201 - 26,298 : 1,105)	9,684	7,791
Fertilized embryo	43,996,849	28,361	2,689 (201 - 26,298 : 917)	9,469	7,561
Polar body	43,132,738	26,716	2,626 (201 - 26,298 : 1,020)	9,246	7,380
2 cell	44,839,836	31,326	2,412 (201 - 26,298 : 917)	9,139	7,254
4 cell	47,675,420	23,122	3,204 (201 - 26,298 : 841)	9,414	7,567
8 cell	45,215,462	27,532	2,564 (201 - 31,183: 1,442)	9,030	7,220
16 cell	49,536,401	33,776	2,470 (201 - 26,298 : 871)	9,470	7,463
32 cell	58,598,783	38,718	2,461 (201 - 26,298 : 927)	11,193	8,597
Blastula	50,083,677	30,553	3,004 (201 - 31,183: 901)	10,994	8,535
Emerged cilia	58,462,746	27,855	3,320 (201 - 31,183: 1,513)	12,153	9,625
Early trochophore	64,464,321	38,443	3,291 (201 - 36,191: 858)	12,980	10,034
Middle trochophore	72,767,170	42,797	3,234 (201 - 36,191: 930)	14,482	11,001
Late trochophore	77,723,477	48,553	3,081 (201 - 36,191: 837)	15,208	11,300
Segmentation	49,350,938	26,509	2,740 (201 - 32,619: 1,318)	11,883	9,030
Total	368,166,154	620,490	846 (201 - 36,191 : 322)	32,880	20,305

 Click here to download Figure Figure1.jpg

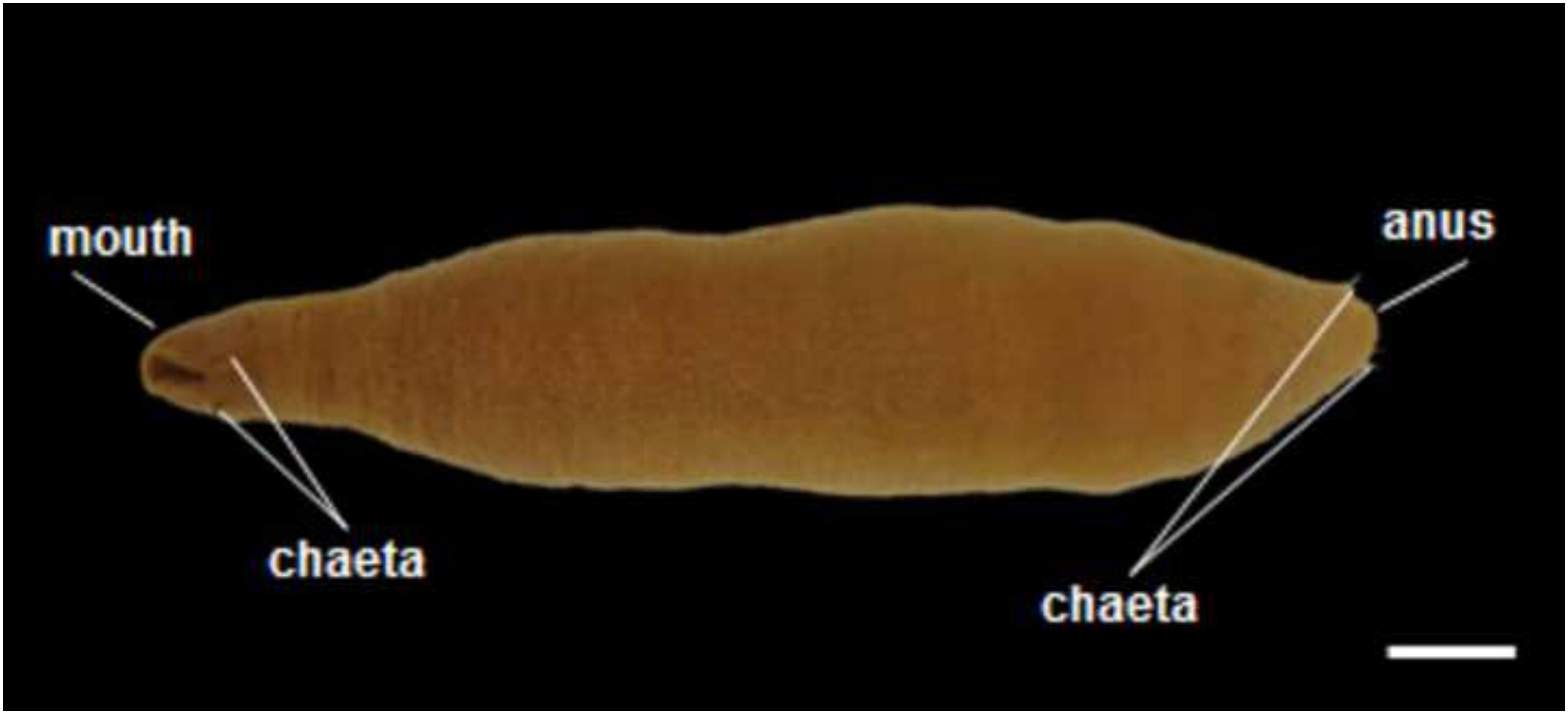

Figure2 Click here to download Figure Revised Figure2.tiff

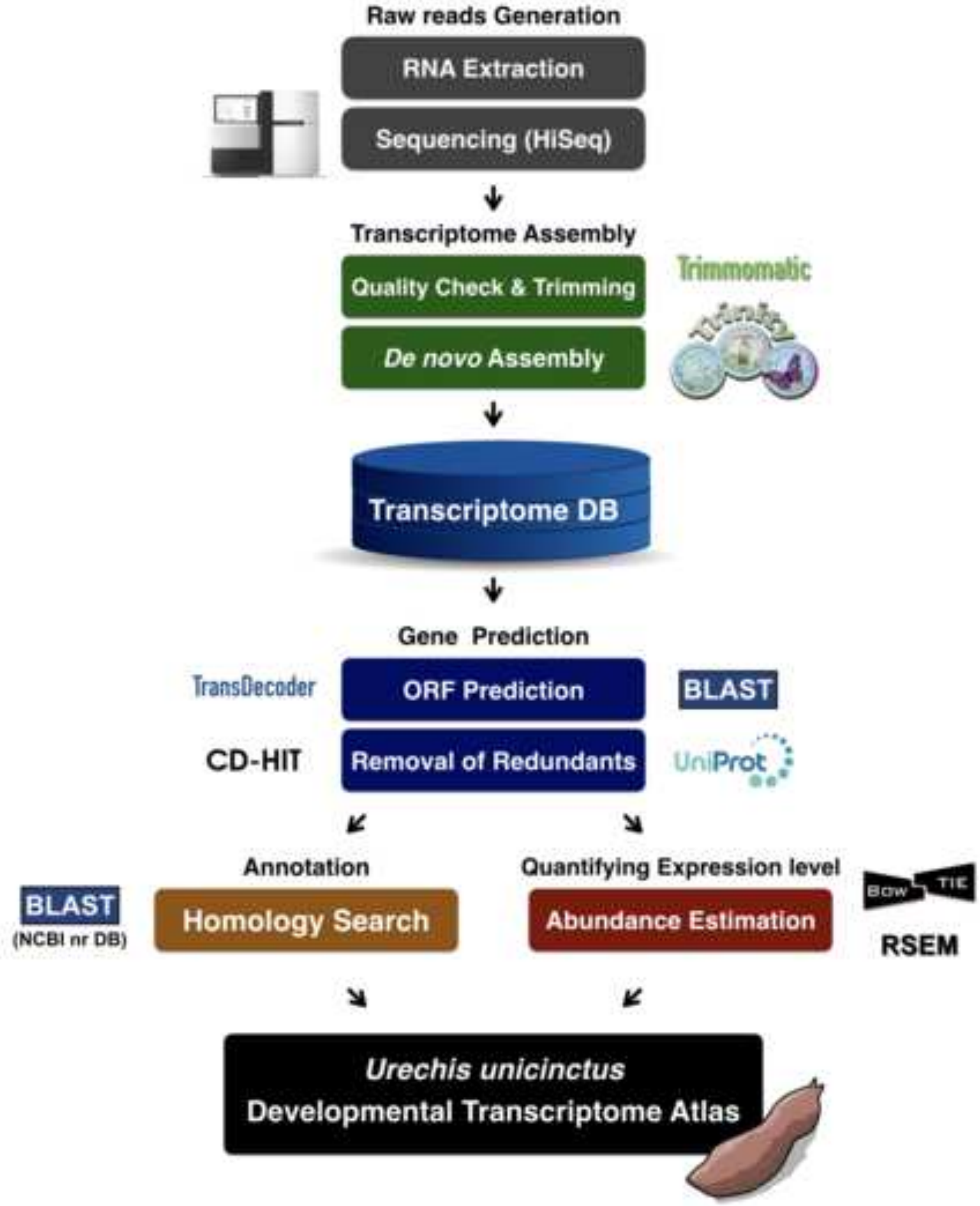

Fig. 2

Figure3

Click here to download Figure Revised Figure3.tiff

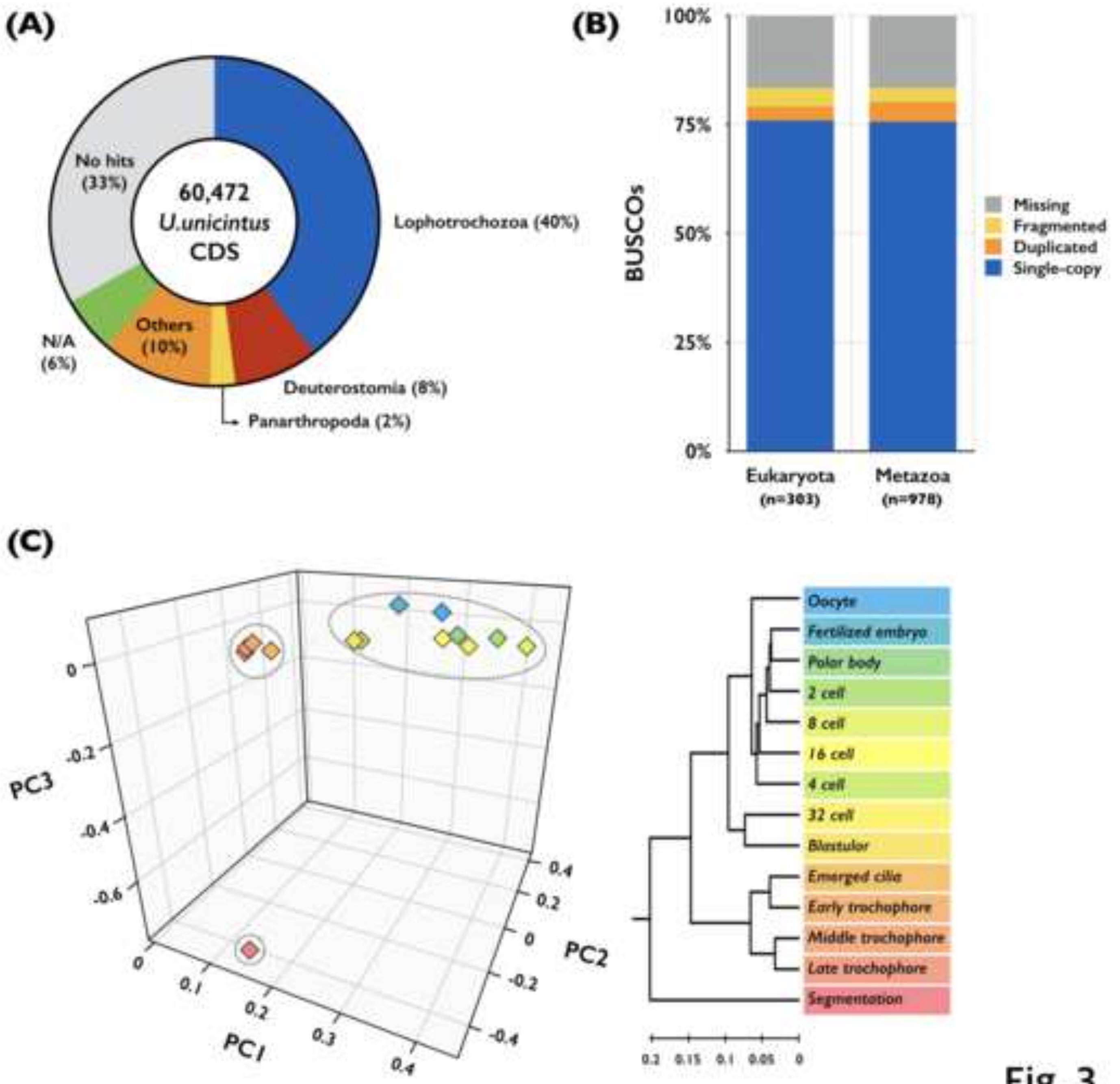

Fig. 3

 Click here to download Figure Revised Figure4.tif

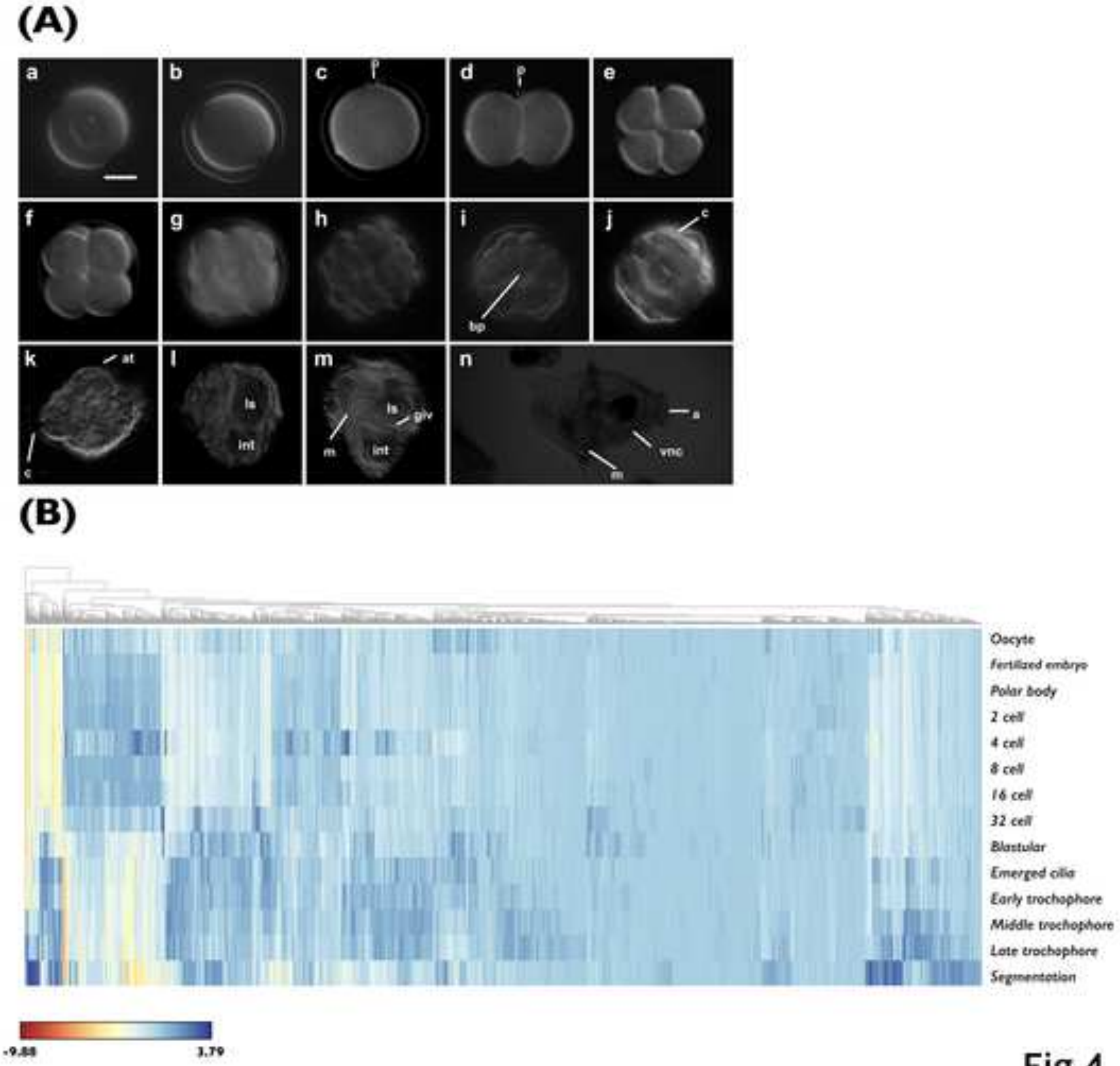

Fig.4

 Click here to download Personal Cover Han et al-Authors Response.docx

Editor in Chief

GigaScience

RE: Ms. Ref. No.: GIGA-D-17-00202

Dear Editor:

Thank you very much for your letter on September 21, 2017.

Our manuscript (Ms. Ref. No.: GIGA-D-17-00202) entitled “The developmental transcriptome atlas of the spoon worm *Urechis unicinctus* (Echiurida: Annelida) ” has now been revised based on feedback from the reviewers and editor. With our resubmission we are including a detailed response to each of the reviewers' comments.

I hope all corrections and revisions in the revised manuscript will be satisfactory.

Sincerely yours,

Sung-Jin Cho, Ph.D.

Associate Professor

Department of Biology (S1-5, 204b)

College of Natural Sciences

Chungbuk National University

52 Naesudong-ro, Heungdeok-gu

Cheongju, Chungbuk 361-763

Republic of Korea

E-mail: sjchobio@chungbuk.ac.kr

***Reviewer #1**: In this study Han and colleagues report the early developmental transcriptome of the spoon worm Urechis unicinctus. This is a valuable resource that certainly will be used by the comparative genomics community and those working on annelid larval development.*

I have a few comments to improve the manuscript:

Comment 1:
1) The authors seem to estimate the genome size of Urechis unicinctus based on k-mer analysis. However, for this they would need to use genomic reads, and not transcriptome reads. This estimate should be deleted, unless it was done on genomic sequences, in which case more detail should be given.

Authors' response:
Although we utilized genomic reads to estimate the genome size of *U. unicinctus*, we deleted this section because reviewer #2 (comment 2; "*It is also not directly relevant to a transcriptomic publication, as genome size is not necessarily reflective of transcriptomic complexity")* also pointed out same issue.

Comment 2:
2)The authors should compare their developmental transcriptome to other studies within (at least) the annelida or the Lophotrochozoa. Placing this study into context by listing other available resources would be valuable for the readers.

Authors' response:
We added one sentence on page 4 (lines13-16) with related references:
"Indeed, data from recently published developmental transcriptomes of other Lophotrochazoans (e.g., Aplysia californica and Platynereis dumerilii) have highlighted insights into molecular mechanisms underlying early development and metamorphosis [6, 7].

Comment 3:
3)The study focuses on larval stages, up to the segmented larva. However, in the abstract the authors write that their resource will be of 'substantial value for understanding how gene repertoires are involved in early stages of development, ontogenic morphogenesis and the formation of an unsegmented body plan." Since no data are reported for later, unsegmented stages, the value of this resource to directly address that problem (i.e. how the unsegmented body forms from a segmented body) is limited. This sentence should be rewritten or data from unsegmented stages should also be included.

Authors' response:
As suggested by the reviewer #1, the sentence has been modified on page 2 (lines 4-7) as following:

"For this reason, transcriptome analyses from various developmental stages of Echiurid can be of substantial value for understanding the precise expression level and the complex regulatory networks during early and larval development."

***Reviewer #2**: This is an interesting article and a useful dataset, which will be the basis for future work both by the authors and related researchers. However, the text as it stands needs*

revision to correct some important points. When this is done, it will be a very handy addition to the published record for a variety of researchers.

Major points:

Comment 1:

-replicates have not been performed for timepoints. This should be clearly stated throughout as it is a major limitation of this study. Lines 44-46 of page 2 and lines 46 - 60 on page 9 have major issues, as without replicates these could be outliers, resulting from PCR, assembly or transcriptional artefacts or other problems. It should also be made clear to the reader that without replication, hypotheses of differential expression are tentative at best. Even just double checking these 12,910 "differentially expressed" contigs in particular to ensure that they are actual genes would be useful.

Authors' response:

We agree with the reviewer that biological replicates may contribute to minimizing the number of false positive genes and detecting these differentially expressed genes statistically. However, this limitation can't be fully overcome without the genome sequence. To point out this limitation, we added one sentence on page 10 (lines 15-18) as following:

"Although this study presents the first large-scale developmental transcriptome dataset for a developmentally interesting animal group, *U. unicinctus* (Echiurida), its global landscape of developmental transcriptome is not yet complete, due to the lack of biological replicates and reference genome sequences."

As already described in the manuscript on page 8 (lines 5-11), we applied several bioinformatics analysis methods to detect putative CDS as following:

"After assembly, open reading frames (ORFs) were predicted using TransDecoder (version 3.0.0) (http://transdecoder.sourceforge.net). To maximize sensitivity for capturing ORFs, all transcripts were aligned against the Uniprot/Swiss-Prot database (http://www.uniprot.org) via BLASTP search with an E-value cutoff of 10^{-5}. Next, ORF length < 100 amino acids were discarded to avoid maintaining transcripts with poor evidence for protein-coding regions. Finally, redundant transcripts with more than 99% sequence identity were removed using CD-HIT (version 4.6.5)"

Comment 2:

-Genomic reads are not described or provided. These should be mentioned or cited, as at present Fig 3A is difficult to assess. It is also not directly relevant to a transcriptomic publication, as genome size is not necessarily reflective of transcriptomic complexity.

Authors' response:

Although we utilized genomic reads to estimate the genome size of *U. unicinctus*, we deleted these statements because this comment is worthy of consideration.

Comment 3:

-how many adult individuals were used to produce embryos? This will be important to assess allelic diversity.

Authors' response:
We modified the sentence on page 5 (lines 10-12) as following:
"We extracted eggs and sperm from one adult female and one male. To obtain *U. unicinctus* embryos, artificial fertilization was performed by mixing the appropriate ratio of sperms and eggs."

Comment 4:

- Particularly given the feeding of trochophore larvae, it is surprising that no assessment of potential contamination is performed or provided. Please address this, perhaps by performing a basic blastn against the non metazoan (or even just the algal and bacterial) section of the nr database for each timepoint and report the degree of any potential contamination (e.g. matching with high similarity) if it is found. If it is found, BUSCO results etc may need to be checked. It would be useful to also cross-reference the 12910 "differentially expressed" set against this.

Authors' response:
We excluded sequences derived from non-metazoan taxa and re-performed all further analyses including BUSCO, PCA, "differentially expressed genes".

Here is the updated version of text.

From Page 8 (line 21) to page 9 (lines 1-14): "To annotate coding sequences (CDS), the resulting 60,472 CDSs were compared against the NCBI non-redundant protein (NR) database (downloaded on April 11, 2017) using BLASTP with an *E*-value cutoff of 10^{-10} and the best blast hit. About 66 % (40,111/60,472) of the CDS were assigned to superphylum-level taxa including Lophotrochozoa (40%), Deuterostomia (8%), and Panarthropoda (2%) (Fig. 3A), which is to be generally expected. For further analysis, we excluded a number of CDSs (18%; 7,231/40,111) by using sequences derived from non-metazoan taxa. When there were multiple coding sequences that mapped to the same gene in the NR database, the sequences with the longest CDS were first assigned to that gene. Based on this criterion, we established a comprehensive transcriptome database of 14 early developmental stages of *U. unicinctus* that comprises 20,305 representative functional protein-coding transcripts. We further assessed the completeness of the *U. unicinctus* development transcriptome using the program BUSCO (bench-marking universal single-copy orthologs) (version 2.0) [14]. 75.9% (230 / 303 genes), and 75.7% (740 / 978 genes) of the eukaryote and metazoan single-copy orthologs were identified, respectively (Fig. 3B)"

and page 10 (lines 11-13): "We identified a total of 12,705 genes that showed dynamic regulation patterns during the differentiation and maturation of U. unicinctus cells (Fig. 4)."

Comment 5:

-Page 7, lines 1-15: estimates of coverage are misleading at best when cross- comparing genomic and transcriptomic data. Transcriptomes are almost certainly non-uniform in coverage. This section needs to be re-considered.

Authors' response:
Please see response to Comment 2.

Comment 6:

-Fig 2 seems to indicate that "quantifying expression level" (right column) was performed on the transcriptome build before removal of redundancy (left hand column). Is this correct? Or was the quantification performed on the CD-HIT - cured set of transcripts? (and quantifying expression level would then follow after CD-HIT, rather than being independent?)

Authors' response:
We performed the "abundance estimation" after removal of redundants. We redrew the Figure 2.

Minor points: (page numbers as per those 'burned in' at bottom of pages, not pdf page numbers, which have additional review pages)

Comment 7:

-Give an assembly download link in the text (and thanks for making these available for review – this was important)

Authors' response:
We added the BioProject link on page 11 (line 8) in the manuscript as following: "(https://www.ncbi.nlm.nih.gov/bioproject/?term=PRJNA394029)"

All further data (will be updated because of "comment 4") have already deposited in the GigaDB, which probably can be provided from Gigascience journal. Here is the list of files we deposited.

1. Urechis_Dev_Draft_Trinity.fasta
   (Draft de novo transcriptome assembly using Trinity)
2. A. Urechis_Dev_Removal_Redundant_mRNA.fasta
   (mRNA sequences after gene prediction and removing redundant)
   B. Urechis_Dev_Removal_Redundant_cds.fasta
   (Coding sequences (nucleotide) after gene prediction and removing redundant)
   C. Urechis_Dev_Removal_Redundant_prot.fasta
   (Coding sequences (amino acid) sequences after gene prediction and removing redundant)
3. A. Urechis_Dev_CDS_M_mRNA.fasta
   (mRNA sequences of representative protein coding sequence)

B. Urechis_Dev_CDS_M_cds.fasta
(Coding sequences (nucleotide) of representative protein coding sequence)

C. Urechis_Dev_CDS_M_prot.fasta
(Coding sequences (amino acid) of representative protein coding sequence)
D. Urechis_Dev_CDS_M.gff3
(Annotation of representative protein coding sequence)
4. Urechis_Dev_CDS_M_UPGMA_tree.nwk
(Dendrogram based on pairwise distance matrices (1-rho, Spearman's correlation coefficient)
5. Urechis_Dev_CDS_M_EXP.tsv
(Expression level (FPKM) of 14 stages)
6. A. Urechis_Dev_CDS_M_BUSCO_eukaryota.zip
(Results of BUSCO analysis using eukaryote single-copy orthologs)
B. Urechis_Dev_CDS_M_BUSCO_metazoa.zip
(Results of BUSCO analysis using metazoan single-copy orthologs)

Comment 8:

pg 2 line 13 - should this be "of echiurid species" (echiurid as an adjective, not a noun?)

Authors' response:
Corrected.

Comment 9:

pg 2 line 26: average - is this the mean, median? N50 useful here?

Authors' response:
The meaning of "average" in our manuscript indicates "mean", but the use of N50 is better than "average length". We updated this section on page 2 (lines 8-10) as following:

"A total of 914 million raw RNA-Seq reads were produced from 14 developmental stages of *Urechis unicinctus*, and were *de novo* assembled into contigs spanning 63,928,225 bp with an N50 length of 2,700 bp."

Comment 10:

pg 5 line 13 - "sandy bottom" is an odd phrasing. "Sandy intertidal areas?"

Authors' response:
It was changed into "intertidal mud flat" on page 5 (line 9).

Comment 11:

-Can any developmental landmarks be given for the early/mid/late trochophore stage, or refer tophotographs?

Authors' response:
The following sentences and reference on page 5 (lines 20-23) and page 6 (lines 1-2) added into the revised manuscript.
"Diagnostic features for each of the three trochophore stages are as follows. The early trochophore is a non-feeding stage. In the middle trochophore, the gastro-intestinal valve opens and the anus appears. Late trochophore has longer cilia of the apical tufts that are replaced by shorter cilia which cover a greater area and the prototroch cilia are longer. The developmental stages of the *U. unicinctus* embryos followed Newby 's classification [8]."

Comment 12:

-What artificial seawater was used?

Authors' response:
We added the sentence on page 5 (lines 13-14) as following:
"The embryos were reared in artificial seawater (Reef crystals (Aquarium Systems, France)) in a plastic case at room temperature (18-20 °C)."

Comment 13:

pg 6 line 10 mRNA (not the mRNAs)

Authors' response:
Corrected.

Comment 14:

pg 6 line 20 how many rounds of PCR in library construction? What adapters used?

Authors' response:
We added and modified a couple of sentences on page 6 (lines 12-22) and page 7 (lines 1-7) as following:
"Total RNA concentration was calculated by Quant-IT RiboGreen (Invitrogen, #R11490). To assess the integrity of the total RNA, samples are run on the TapeStation RNA screentape (Agilent, #5067-5576). Only high-quality RNA preparations, with RIN greater than 7.0, were used for RNA library construction. A library was independently prepared with 1ugg of total RNA for each sample by Illumina TruSeq Stranded Total RNA Sample Prep Kit (Illumina, Inc., San Diego, CA, USA). The rRNA in total RNA is depleted by Ribo-Zero kit. After the rRNA is depleted, the remaining RNA is purified, fragmented and primed for cDNA synthesis. The cleaved RNA fragments are copied into first strand cDNA using reverse transcriptase and random hexamers. This is followed by second strand cDNA synthesis using DNA Polymerase I, RNase H and dUTP. These cDNA fragments then go through an end repair process, the addition of a single 'A' base, and then ligation of the adapters. The products are then purified and enriched with PCR to create the final cDNA library. The libraries were quantified using qPCR according to the qPCR Quantification Protocol Guide (KAPA Library Quantification kits for Illumina Sequencing platforms) and qualified using the TapeStation D1000 ScreenTape (Agilent Technologies, Waldbronn, Germany). Indexed

libraries were then submitted to a Illumina Hiseq 4000 (Illumina, Inc., San Diego, CA, USA), and the paired-end (2×100 bp) sequencing was performed."

Comment 15:

pg 8 line 22, and Fig 3B. Is this best blast hits, please report this in the figure legend and text if so.

Authors' response:

Yes, it is the best blast hit. We updated this section and figure legend on (1) page 8 (lines 21-22), page 9 (line 1), and (2) page 15 (lines 6-7) as following:

(1) "To annotate coding sequences (CDS), the resulting 60,472 CDSs were compared against the NCBI non-redundant protein (NR) database (downloaded on April 11, 2017) using BLASTP with an *E*-value cutoff of 10^{-10} and the best blast hit."

(2) "Superphylum distribution for homology search of *U. unicinctus* coding sequences against the NR database using the best blast hit."

Comment 16:

Page 8 lines 48 - 53: This seems a deeply flawed comparison, and as you acknowledge, compares very non-comparable things. Maybe find recent transcriptomes that have used BUSCO as an assessment tool and use these as exemplars? All of these were published before BUSCO.

Authors' response:

Without a reference genome, highly accurate whole transcriptome assembly from short reads will be a tough challenge. So far, there have been no reports of the completeness assessment of the de novo transcriptome assemblies using the BUSCO scores. Therefore, we deleted these sentences in our revised manuscript.

Comment 17:

-page 9 line 47: 10 fold (total)? or is this a log2(a/b) measure? The FDR also seems to be communicated oddly. Do you mean a p-value cutoff for FDR of 0.001?

Authors' response:

The fold value is 10-fold, and the score 0.001 is for FDR cut-off but not for p-value.
To clarify and more fully explain its meaning, we modified the sentence on page 10 (lines 9-11) as following:
"In an additional analysis, a gene whose expression level was significantly changed (≥ 10-fold and FDR adjusted *P* value ≤ 0.1%) in at least one comparison, was defined as a developmentally regulated gene."

Comment 18:

Pg 13 line 5: I think it better to say "Adult Urechis unicinctus" (no "worm of)

Authors' response:
Corrected.

Comment 19:

Line 44: scale spelled "sclae"

Authors' response:
Corrected.

Comment 20:

In several locations and in data - I understand the use of FKPM, but TPM may be a more useful measure - it allows the cross - comparison of these results between samples, while FKPM does not.Consider providing this information if you have it.

Authors' response:
We agree, and investigated whether there is difference in expression profiles based on between TPM and FPKM values. In all 14 samples, the expression level values between TPM and FPKM are highly coupled (see the below table), indicating that our results could not be influenced by gene expression measurements.

***p < 2.2e-16* with all comparisons**

	**TPM vs FPKM**
**Stage**	**Spearman's (ρ)**
oocytes	0.9998206
fertilized egg	0.9998886
polar body	0.9999137
2 cell	0.9998949
4 cell	0.9998529
8 cell	0.9999356
16 cell	0.9998927
32 cell	0.999884
blastular	0.9999695
emerged cilia	0.9999853
early trochophore	0.999974
middle trochophore	0.9999774
late trochophore	0.9999885
segementation	0.9999447

Comment 21:

- the references are in an odd font, which may require changing depending on journal preferences.

Authors' response:
Corrected.

Reviewer #3:

Comment 1:

The paper presents transcriptomic libraries for 14 developmental stages of an echiurid species. This is within the scope of the journal. Hence, I suggest accepting the paper after a major revision. My major concern is the introduction. The introduction does not adequately reflect the literature on the position of Echiura. With respect to morphological data the studies of Hessling concerning the nervous system are missing. This among others also reflected in the erroneous statement that the presence of a larval nervous system includes placement in Annelida. However, this is in this way not correct. Larval nervous systems occur throughout the animal kingdom, it is the specific features of this system, which support this placement and require citing the works by Hessling. Moreover, recent studies using phylogenomic data concerning the phylogeny of Annelida are also not mentioned (see below). Finally, for understanding the early evolution of Lophotrochozoa Echiura are irrelevant as they are highly derived annelid taxa. Echiura are more interesting in understanding the plasticity of body plan evolution in general, but this independent of their position within Lophotrochozoa. Moreover, citations supporting the claim that Urechis unicinctus attracted special attention for evo-devo are lacking and should be provided.

Authors' response:
We appreciate the reviewer's feedback and agree with the suggested changes. In the introduction, we have now corrected the incorrect statement about the larval nervous system and have added references to Hessling's work. We have also added references to the recent phylogenomic research on the placement of Echiurids within Annelida. We also have changed the sentence about evo-devo to emphasize instead the potential use of Urechis to understand the evolutionary loss of adult segmentation. Finally, we made all corrections according to your minor comments (see all changes below).

Here is the updated text on page 4 (lines 3-21):

"Within the major annelid groups, Echiurida (also called the 'marine spoon worms') is represented by a morphologically and ontogenetically unique assemblage that includes approximately 165 species, most of which lacks segmentation in adults, although they possess annelid-like morphological and developmental features including the organization of the larval nervous system [1]. *They were once considered a separate metazoan phylum, but reevaluation of morphological and molecular data indicated that Echiurida is nested within the Annelida* representing one of three animal phyla with body segmentation [1-5]. In this respect, transcriptome analyses from various developmental stages of Echiurida are of substantial value for understanding *precise expression levels and the complex regulatory*

networks involved in early and larval development. Indeed, data from recently published developmental transcriptomes of other Lophotrochazoans (e.g., Aplysia californica and Platynereis dumerilii) have highlighted insights into molecular mechanisms underlying early development and metamorphosis [6, 7].

Urechis unicinctus is an Echiuran species that inhabits burrows in intertidal soft sediments (Fig. 1). The *Urechis* genus may hold important clues to the genetic basis of the evolutionary gain and loss of segmentation, due to its nested position within Annelida (i.e., sister to capitellid polychaetes), a Lophotrochozoan phylum that is represented by a diverse group of segmented worms [2, 5]."

Reviewer #3's minor comments:

Comment 2:

Page 2, Line 6: delete "the phylum"

Authors' response:
Authors want to keep "the phylum" in its original place.

Comment 3:

Page 2, Line 8: change to "unlike most other annelid"

Authors' response:
changed

Comment 4:

Page 2, Line 16: insert space between "development, ontogenetic"

Authors' response:
corrected

Comment 5:

Page 2, Line 57: insert dot between "stages. These"

Authors' response:
corrected

Comment 6:

Page 4, Line 36: delete dot between "(Fig. 1). This"

Authors' response:
modified

Comment 7:

Page 4, Line 48: delete "the" at the end of the line

Authors' response:
deleted

Comment 8:

Page 4, Line 51: "profiles" instead of "profiling"

Authors' response:
changed

Comment 9:

Page 7, Line 2: describe how the k-mer analysis was conducted in detail

Authors' response:
We deleted this section because review #1 (comment 1; "*The authors seem to estimate the genome size of Urechis unicinctus based on k-mer analysis. However, for this they would need to use genomic reads, and not transcriptome reads. This estimate should be deleted, unless it was done on genomic sequences, in which case more detail should be given") and* reviewer #2 (comment 2; "*It is also not directly relevant to a transcriptomic publication, as genome size is not necessarily reflective of transcriptomic complexity")* also pointed out same issue.

Comment 10:

Page 7, Lines 18-25: I do not understand what has been done here. Concatenation means that individual sequences are attached to each other one after the other, but I think this is not meant here. Rather assume that the reads from all 14 libraries were pooled together for assembly of the data. Please clarify this point.

Authors' response:
We modified "concatenated" into "pooled".

Comment 11:

Page 8, Line 7: How did you correct the expression profiles for the uneven sequencing depth among the different libraries? The range is from 43 million reads to 140 million and hence the latter is more than three times higher.

Authors' response:
The FPKM value we used in our analysis is quantifying RNA-seq data by normalizing (1) read length and (2) the number of reads sequenced. According to the latter case, the expression profiles among libraries with uneven sequencing depth have been already normalized in order to compare expression levels of genes among these libraries.

Comment 12:

Page 8, Line 46: include a comma in "were identified,"

Authors' response:
corrected

Comment 13:

Page 13, Line 24: This is not a phylogenetic tree, which by definition can only be reconstructed between species and not developmental stages between individuals. Change wording.

Authors' response:
This sentence on page 15 (lines 8-10) was changed as following:

"Result of principal component analysis (PCA) and a dendrogram of transcriptomes of 14 *U. unicinctus* developmental stages based on pairwise distance matrices (1 - ρ, Spearman's correlation coefficient)."

Comment 14:

Page 13, Line 44: "mouth" instead of "mouse"

Authors' response:
corrected

Comment 15:

Page 13, Line 44: insert a space between "50µm. (B)"

Authors' response:
a space is inserted

Comment 16:

Citations missing:
Hessling, R. & Westheide, W. (2002) Are Echiura derived from a segmented ancestor? Immunohistochemical analysis of the nervous system in developmental stages of Bonellia viridis.
Journal of Morphology, 252, 100-113.
Hessling, R. (2002) Metameric organisation of the nervous system in developmental stages of Urechis
caupo (Echiura) and its phylogenetic implications. Zoomorphology, 121, 221-234.
Hessling, R. (2003) Novel aspects of the nervous system of Bonellia viridis (Echiura) revealed by
the combination of immunohistochemistry, confocal laser-scanning microscopy and three-dimensional
reconstruction. Hydrobiologia, 496, 225-239.
Struck, T.H., Paul, C., Hill, N., Hartmann, S., Hösel, C., Kube, M., Lieb, B., Meyer, A., Tiedemann, R., Purschke, G. & Bleidorn, C. (2011) Phylogenomic analyses unravel annelid evolution.
Nature, 471, 95-98.
Weigert, A., Helm, C., Meyer, M., Nickel, B., Arendt, D., Hausdorf, B., Santos, S.R., Halanych,
K.M., Purschke, G., Bleidorn, C. & Struck, T.H. (2014) Illuminating the base of the annelid tree
using transcriptomics. Molecular Biology and Evolution, 31, 1391-1401.
Struck, T.H., Golombek, A., Weigert, A., Franke, Franziska A., Westheide, W., Purschke, G., Bleidorn, C. & Halanych, K.M. (2015) The Evolution of Annelids Reveals Two Adaptive Routes to the
Interstitial Realm. Current Biology, 25, 1993-1999.
Andrade, S.C.S., Novo, M., Kawauchi, G.Y., Worsaae, K., Pleijel, F., Giribet, G. & Rouse, G.W.
(2015) Articulating "archiannelids": Phylogenomics and annelid relationships, with emphasis on
meiofaunal taxa. Molecular Biology and Evolution.

Authors' response:
Added.
