# Supplementary material for: The developmental transcriptome atlas of the spoon worm Urechis unicinctus (Echiurida: Annelida)
Source: Gigascience. 2018 Feb 15;7(3):giy007. doi: 10.1093/gigascience/giy007 (PMC5863216; doi:10.1093/gigascience/giy007)
Supplement: GIGA-D-17-00202_Revision_2.pdf [file giy007_giga-d-17-00202_revision_2.pdf]

GigaScience

The developmental transcriptome atlas of the spoon worm Urechis unicinctus (Echiurida: Annelida)

--Manuscript Draft--

**Manuscript Number:**	GIGA-D-17-00202R2
**Full Title:**	The developmental transcriptome atlas of the spoon worm Urechis unicinctus (Echiurida: Annelida)
**Article Type:**	Data Note
**Funding Information:**	Ministry of Oceans and Fisheries (20140428) — Dr Joong-Ki Park<br>Basic Science Research Program through the National Research Foundation of Korea (NRF-2016R1D1A1B03933412) — Dr Sung-Jin Cho
**Abstract:**	Background: Echiurida is one of the most intriguing major subgroups of annelida, because unlike most other annelids, echiurids lack metameric body segmentation as adults. For this reason, transcriptome analyses from various developmental stages of Echiurid can be of substantial value for understanding precise expression levels and the complex regulatory networks during early and larval development.<br>Finding: A total of 914 million raw RNA-Seq reads were produced from 14 developmental stages of Urechis unicinctus, and were de novo assembled into contigs spanning 63,928,225 bp with an N50 length of 2,700 bp. The resulting comprehensive transcriptome database of the early developmental stages of U. unicinctus consists of 20,305 representative functional protein-coding transcripts. Approximately 66 % of unigenes were assigned to superphylum-level taxa, including Lophotrochozoa (40%). The completeness of the transcriptome assembly was assessed using BUSCO, and 75.7 % of the metazoan single-copy orthologs were presented in our transcriptome database. We observed three distinct patterns of global transcriptome profiles from 14 developmental stages, and identified a total of 12,705 genes that showed dynamic regulation patterns during the differentiation and maturation of U. unicinctus cells.<br>Conclusions: We present the first large-scale developmental transcriptome dataset of U. unicinctus and provide a general overview of the dynamics of global gene expression changes during its early developmental stages. The analysis of time-course gene expression data is a first step toward understanding the complex developmental gene regulatory networks in U. unicinctus, and will furnish a valuable resource for analyzing the functions of gene repertoires in various developmental phases.
**Corresponding Author:**	Sung-Jin Cho, Ph.D.<br>Chungbuk National University<br>Cheongju, Chungbuk KOREA, REPUBLIC OF
**Corresponding Author Secondary Information:**	
**Corresponding Author's Institution:**	Chungbuk National University
**Corresponding Author's Secondary Institution:**	
**First Author:**	Chungoo Park, Ph.D.
**First Author Secondary Information:**	
**Order of Authors:**	Chungoo Park, Ph.D.
	Yong-Hee Han
	Sung-Gwon Lee
	Kyoung-Bin Ryu
	Jooseong Oh
	Elizabeth Kern, Ph.D.

Powered by Editorial Manager® and ProduXion Manager® from Aries Systems Corporation

	Joong-Ki Park, Ph.D.
	Sung-Jin Cho, Ph.D.
**Order of Authors Secondary Information:**	
**Response to Reviewers:**	Reviewer reports:<br>Reviewer #2:<br>Revisions have been made as appropriate.<br><br>There are a few minor spelling errors (e.g. "Lophotrochazoans" pg 4 line 14) and for Fig 3A it would be good to define what is meant by N/A (c.f. "Others" and "No hits") that should be corrected in proof.<br>Authors' response:<br>As suggested by the reviewer#2, we corrected " Lophotrochozoans" instead of " Lophotrochazoans ".<br>"N/A" indicate the species group without assigned superphylum-level. To clarify this point, it has been changed to "Unknown" and updated the Figure 3A.<br><br>Reviewer #3: The manuscript has improved, but still some points need to be addressed:<br>1.) The authors did not include all the citations I asked to add. If this is due to a citation limit<br>not stated in the "Instruction to Authors" the authors should exclude Struck et al. (2011) and<br>Andrade et al. (2015) as the review by Weigert and Bleidorn cover the recent phylogenomic studies on annelid phylogeny and instead include the two Hessling (2002) papers as they present the results on the nervous system.<br>Authors' response:<br>We have added references of Hessling's works (see reference no. 1 and 2).<br><br><br>Reviewer #3:<br>2.) Linnean categories like phylum have no biological meaning and are just an artificial categorical level. Therefore, their usage should be restricted to the least possible and whenever possible neutral should be used. Hence, the following changes should be made:<br>Page 2, Line 2: delete "the phylum"<br>Page 4, Line 8: "taxon" instead of "phylum"<br>Page 4, Line 9: "the three major animal taxa" instead of "the three animal phyla"<br>Page 4, Line 20: "taxon" instead of "phylum"<br>Page 10, Line 22: "annelid" instead of "phylum"<br>Authors' response:<br>As suggested by the reviewer#3, we corrected "the phylum (Page 2, Line 2)" and "annelid" instead of "phylum (Page 10, Line 20)"<br>Authors hope to keep "the phylum (Page 4, Line 7; Page 4, Line 9; Page 4, Line 20)" in its original place as taxonomic category of Linnean system often provides some information related to the history of its systematic position.<br><br><br>Reviewer #3:<br>3.) Minor changes:<br>Page 4, Line 9: delete "the" before Annelida (Annelida is grammatically a proper name and does not need a definitive article)<br>Authors' response:<br>Deleted.<br><br>Reviewer #3:<br>Page 7, Lines 6-10: The two sentences are confusing. It sounds like you did one round of sequencing and then another round on the same material. Please rephrase.<br>Authors' response:<br>The first sentence was removed.<br><br>Reviewer #3:<br>Page 9, Line 19: "3C" instead of "3D"

	Page 15, Figure legend 3: The description for Figure 3D is lacking.<br>Authors' response:<br>Updated Figure 3 is composed of three panels (3A, 3B, and 3C). Figure 3C is two results (PCA and dendrogram) because they both indicate the transcriptome comparison with different visualization methods.
**Additional Information:**	
**Question**	**Response**
Are you submitting this manuscript to a special series or article collection?	No
**Experimental design and statistics**<br><br>Full details of the experimental design and statistical methods used should be given in the Methods section, as detailed in our Minimum Standards Reporting Checklist. Information essential to interpreting the data presented should be made available in the figure legends.<br><br>Have you included all the information requested in your manuscript?	Yes
**Resources**<br><br>A description of all resources used, including antibodies, cell lines, animals and software tools, with enough information to allow them to be uniquely identified, should be included in the Methods section. Authors are strongly encouraged to cite Research Resource Identifiers (RRIDs) for antibodies, model organisms and tools, where possible.<br><br>Have you included the information requested as detailed in our Minimum Standards Reporting Checklist?	Yes
**Availability of data and materials**<br><br>All datasets and code on which the conclusions of the paper rely must be either included in your submission or deposited in publicly available repositories (where available and ethically appropriate), referencing such data using a unique identifier in the references and in the "Availability of Data and Materials" section of your manuscript.<br><br>Have you have met the above requirement as detailed in our Minimum Standards Reporting Checklist?	Yes

Manuscript

Click here to download Manuscript Han et al-3rd Revised_ED1-2-18.docx

Data Note

The developmental transcriptome atlas of the spoon worm *Urechis unicinctus* (Echiurida: Annelida)

Chungoo Park[2#], Yong-Hee Han[1#], Sung-Gwon Lee[2#], Kyoung-Bin Ryu[1], Jooseong Oh[2], Elizabeth M. A. Kern[3], Joong-Ki Park[3*], Sung-Jin Cho[1*]

[1]School of Biological Sciences, College of Natural Sciences, Chungbuk National University, Cheongju, Chungbuk 28644, Republic of Korea

[2]School of Biological Sciences and Technology, Chonnam National University, Gwangju 61186, Republic of Korea

[3]Division of EcoScience, Ewha Womans University, Seoul 03760, Republic of Korea

[#] These authors contributed equally to this work.

*Corresponding Authors.

E-mail addresses:

Sung-Jin Cho, sjchobio@chungbuk.ac.kr. Tel: +82-43-261-2294. Fax: +82-43-260-2298.Joong-Ki Park, jkpark@ewha.ac.kr. Tel: +82-2-3277-5948. Fax: +82-2-3277-2385.

Abstract

Background: Echiurida is one of the most intriguing major subgroups of annelida, because unlike most other annelids, echiurids lack metameric body segmentation as adults. For this reason, transcriptome analyses from various developmental stages of echiurid species can be of substantial value for understanding precise expression levels and the complex regulatory networks during early and larval development.

Findings: A total of 914 million raw RNA-Seq reads were produced from 14 developmental stages of *Urechis unicinctus*, and were *de novo* assembled into contigs spanning 63,928,225 bp with an N50 length of 2,700 bp. The resulting comprehensive transcriptome database of the early developmental stages of *U. unicinctus* consists of 20,305 representative functional protein-coding transcripts. Approximately 66 % of unigenes were assigned to superphylum-level taxa, including Lophotrochozoa (40%). The completeness of the transcriptome assembly was assessed using BUSCO and 75.7 % of the metazoan single-copy orthologs were presented in our transcriptome database. We observed three distinct patterns of global transcriptome profiles from 14 developmental stages and identified a total of 12,705 genes that showed dynamic regulation patterns during the differentiation and maturation of *U. unicinctus* cells.

Conclusions: We present the first large-scale developmental transcriptome dataset of *U. unicinctus* and provide a general overview of the dynamics of global gene expression changes during its early developmental stages. The analysis of time-course gene expression data is a first step toward understanding the complex developmental gene regulatory networks in *U. unicinctus* and will furnish a valuable resource for analyzing

the functions of gene repertoires in various developmental phases.

Keywords: *Urechis unicinctus*, Echiurida, Developmental transcriptome, RNA-Seq, *de novo* assembly

Data Description

Background

Within the major annelid groups, Echiurida (also called the ‘marine spoon worms’) is represented by a morphologically and ontogenetically unique assemblage that includes approximately 165 species, most of which lack segmentation as adults, although they possess annelid-like morphological and developmental features including the organization of the larval nervous system [1, 2]. They were once considered a separate metazoan phylum, but reevaluation of morphological and molecular data indicated that Echiurida is nested within Annelida, which represents one of the three major animal phyla with body segmentation [3-7]. In this respect, transcriptome analyses from various developmental stages of echiurid species are of substantial value for understanding precise expression levels and the complex regulatory networks involved in early and larval development. Indeed, data from recently published developmental transcriptomes of other Lophotrochozoans (e.g., *Aplysia californica* and *Platynereis dumerilii*) have highlighted insights into molecular mechanisms underlying early development and metamorphosis [8, 9].

Urechis unicinctus is an echiuran species that inhabits burrows in soft sediments in intertidal areas (Fig. 1). The *Urechis* genus may hold important clues to the genetic basis of the evolutionary gain and loss of segmentation, due to its nested position within Annelida (i.e., sister to capitellid polychaetes), a Lophotrochozoan phylum that is represented by a diverse group of segmented worms [4, 7]. However, current knowledge is limited on the molecular mechanisms that underlie the ontogeny

of *U. unicinctus*. The goal of this study is to enhance our understanding of gene expression during embryonic development. Here we report the transcriptome profiles (generated with the Illumina HiSeq platform) of developing embryos of *U. unicinctus*. Transcriptome sequencing data assist in the discovery of the roles of genes involved in various embryological and larval development processes. As the first large-scale transcriptomic dataset for *U. unicinctus,* this resource will help in the validation of development-specific gene features predicted by the genome.

Sample collection, embryo culture, and RNA isolation

Adults of *U. unicinctus* were collected from intertidal mud flats on the southern coast of South Korea. We extracted eggs and sperm from one adult female and one male. To obtain *U. unicinctus* embryos, artificial fertilization was performed by mixing the appropriate ratio of sperms and eggs.

Embryos were reared in artificial seawater (Reef crystals (Aquarium Systems, France)) in a plastic case at room temperature (18-20 °C). The late trochophore, a typical larval stage in which the intestinal tract is formed, was fed with a microalgae called *Isochrysis galbana*. Reared embryo samples were collected at each of the following stages: 0 h (unfertilized egg), 0.5 h post fertilization (fertilized egg), polarbody cell, 2 cell, 4 cell, 8 cell, 16 cell, 32 cell, blastula, emerged cilia, early trochophore (day 1), middle trochophore (day 2), late trochophore (day 5), and segmentation stage (day 30~45). Diagnostic features for each of the three trochophore stages are as follows. The early trochophore is a non-feeding stage. In the middle trochophore, the gastro-intestinal valve opens and the anus appears. In the late

trochophore, the longer cilia of the apical tufts are replaced by shorter cilia which cover a greater area, and the prototroch cilia are longer. These developmental stages follow Newby's classification [10].

Total RNA was isolated from the embryos of the above samples using TRIZOL reagent (Invitrogen, Carlsbad, CA, USA) following the manufacturer's instructions. The purity and integrity of the total RNA isolated from each embryo sample were examined using a Nanodrop 2000C spectrophotometer (Thermo Scientific, Waltham, MA, USA) and Bioanalyzer 2100 (Agilent Technologies, Palo Alto CA, USA). Adult images were taken on a Canon EOS 550D, and embryo bright-field images were taken on a Leica DM6 B microscope using DIC optics.

TruSeq Stranded Ribo-Zero library preparation and sequencing

Total RNA concentration was calculated by Quant-IT RiboGreen (Invitrogen, #R11490). To assess the integrity of the total RNA, samples were run on TapeStation RNA screentape (Agilent, #5067-5576). Only high-quality RNA preparations, with RIN greater than 7.0, were used for RNA library construction. A library was independently prepared with 1μg of total RNA for each sample using an Illumina TruSeq Stranded Total RNA Sample Prep Kit (Illumina, Inc., San Diego, CA, USA). The rRNA in total RNA was depleted by a Ribo-Zero kit. After the rRNA was depleted, the remaining RNA was purified, fragmented and primed for cDNA synthesis. The cleaved RNA fragments were copied into first strand cDNA using reverse transcriptase and random hexamers. This was followed by second strand cDNA synthesis using DNA Polymerase

I, RNase H and dUTP. These cDNA fragments then underwent an end repair process, the addition of a single ‘A’ base, and ligation of the adapters. The products were then purified and enriched with PCR to create the final cDNA library. The libraries were quantified using qPCR according to the qPCR Quantification Protocol Guide (KAPA Library Quantification kits for Illumina Sequencing platforms) and qualified using the TapeStation D1000 ScreenTape (Agilent Technologies, Waldbronn, Germany).The resulting samples were sequenced on the Illumina HiSeq 2000 system with a paired-end read with 101 cycles or the Illumina HiSeq 4000 system with a paired-end read with 151 cycles (Table 1). The experimental procedures and complete assembly pipeline are summarized in Fig. 2.

Transcriptome preprocessing and *de novo* assembly

After completion of the sequencing run, to obtain high-quality clean reads from the raw data (i.e., removing those containing adapter sequences, poly-N sequences, or low quality bases), we performed quality-based trimming and filtering using Trimmomatic version 0.33 (Trimmomatic, RRID:SCR_011848) [11] with the parameters ILLUMINACLIP:TruSeq3-PE-2.fa:2:30:10 LEADING:3 TRAILING:3 SLIDINGWINDOW:4:15 MINLEN:36 for the 101 bp library (or MINLEN:50 for the 151 bp library). An average of 63 million clean reads per sample was obtained (Table 1).

Before *de novo* assembly, all clean reads were pooled without normalization of read abundance, even though the use of all merged reads may require progressively

increasing assembly time and memory usage, in order to obtain a comprehensive reference transcriptome database. The merged reads were used for *de novo* transcriptome assembly using Trinity version 2.1.1 (Trinity, RRID:SCR_013048) [12] with default parameters. The resulting assembled transcriptome consisted of 620,490 transcripts with an N50 value of 846 bp (Table 2). After assembly, open reading frames (ORFs) were predicted using TransDecoder (version 3.0.0) (http://transdecoder.sourceforge.net). To maximize sensitivity for capturing ORFs, all transcripts were aligned against the Uniprot/Swiss-Prot database (http://www.uniprot.org) via BLASTP search with an *E*-value cutoff of 10^{-5}. Next, ORF length < 100 amino acids were discarded to avoid maintaining transcripts with poor evidence for protein-coding regions. Finally, redundant transcripts with more than 99% sequence identity were removed using CD-HIT (version 4.6.5) [13], producing a total of 60,472 non-redundant ORFs. These sequences span 63,928,225 bp with an N50 length of 2,700 bp.

To quantify expression levels, the reads for each library were mapped independently to the reference *U. unicinctus* transcriptome sequences using Bowtie version 2.2.6 (Bowtie, RRID:SCR_005476) [14], and expression levels of these transcripts were estimated with RSEM version 1.2.26 (RSEM, RRID:SCR_013027) [15]. The unit of expression level is referred to as fragment per kilobase of transcript per million fragments mapped (FPKM) in our analyses.

Annotation

To annotate coding sequences (CDS), the resulting 60,472 CDSs were compared against the NCBI non-redundant protein (NR) database (downloaded on April 11, 2017) using BLASTP with an *E*-value cutoff of 10^{-10} and the best BLAST hit. About 66 % (40,111/60,472) of the CDS were assigned to superphylum-level taxa including Lophotrochozoa (40%), Deuterostomia (8%), and Panarthropoda (2%) (Fig. 3A), which is to be generally expected. For further analysis, we excluded a number of CDSs (18%; 7,231/40,111) by using sequences derived from non-metazoan taxa. When there were multiple coding sequences that mapped to the same gene in the NR database, the sequences with the longest CDS were first assigned to that gene. Based on this criterion, we established a comprehensive transcriptome database of 14 early developmental stages of *U. unicinctus* that comprises 20,305 representative functional protein-coding transcripts. We further assessed the completeness of the *U. unicinctus* development transcriptome using the program BUSCO (bench-marking universal single-copy orthologs) version 2.0 (BUSCO, RRID:SCR_015008) [16]. 75.9% (230 / 303 genes), and 75.7% (740 / 978 genes) of the eukaryote and metazoan single-copy orthologs were identified, respectively (Fig. 3B).

Transcriptome comparisons

To show that gene expression reflects development-specific differentiation and maturation processes, we built expression distance matrices for each developmental stage and constructed a gene expression tree (Fig. 3C). Two major transitions in expression patterns were observed: (1) blastula to emerged cilia and (2) late trochophore

to segmentation. These transitions divided the 14 *U. unicinctus* developmental stages into three phases: the oocyte, polar body, fertilized, 2-, 4-, 8-, 16-, 32-cell embryo, and blastula stages make up Phase I; the emerged cilia, early-, middle-, and late-trochophore stages compose Phase II; and the segmentation stage makes up to Phase III. These three distinct phases of global transcriptome profiles covering 14 developmental stages were supported by principal component analysis (PCA), which was performed using the "prcomp" function in the "stats" package in R (version 3.2.4) (Fig. 3C). These results suggest that developmental stages are well characterized by our transcription profiles, and the differential gene expression profiles presented in this study will be useful for further study of ontogenic processes at the gene expression level.

In an additional analysis, a gene whose expression level was significantly changed (≥ 10-fold and FDR adjusted *P* value ≤ 0.1%) in at least one comparison was defined as a developmentally regulated gene. We identified a total of 12,705 genes that showed dynamic regulation patterns during the differentiation and maturation of *U. unicinctus* cells (Fig. 4). Note that we used the TMM (trimmed mean of M values) normalization [17] provided by edgeR bioconductor package for R for this test.

Although this study presents the first large-scale developmental transcriptome dataset for a developmentally interesting animal group, *U. unicinctus* (Echiurida), the global landscape of its developmental transcriptome is not yet complete, due to the lack of biological replicates and reference genome sequences.

In summary, we present the first large-scale, developmental stage-specific transcriptome dataset for *U. unicinctus*, and provide a general overview of the dynamics

of global gene expression changes at different developmental stages. These data will fill an important gap in annelid-wide comparisons of gene expression patterns, and will lead to a better understanding of gene repertoires involved in different developmental stages and of complex developmental gene regulatory networks.

Availability of supporting data

All raw sequencing data used for assembly have been deposited in the NCBI database under the accession numbers SRX2999418 to SRX2999431, associated with BioProject PRJNA394029. Additional data further supporting the results of this article, including the transcriptome assembly, annotations and BUSCO results, can be found in the *GigaScienc*e repository, GigaDB [18].

Abbreviations

bp: base pairs; BUSCO: Bench-marking universal single-copy orthologs; CDS: Coding sequence; FDR: False discovery rate; FPKM: Fragments per kilobase of transcript per million mapped reads; Gb: Gigabases; ORFs: Open reading frames; PCA: Principal components analysis; RNA-Seq: High-throughput messenger RNA sequencing; TMM: Trimmed mean of M values.

Competing interests

The authors declare that they have no competing interests.

Authors' contributions

CP and SJC designed the study; JKP contributed to the project coordination; YHH, KBR, and SJC performed the experiments; SGL, JO, and CP analyzed the data and evaluated the conclusions; CP, SJC, JKP, SGL and EMAK wrote the paper; All authors read and approved the final manuscript.

Acknowledgements

This research was supported by a grant from the Collaborative Genome Program (20140428) funded by the Ministry of Oceans and Fisheries, Korea to CP, SJC, JKP. This research was supported by Basic Science Research Program through the National Research Foundation of Korea (NRF) funded by the Ministry of Education (NRF-2016R1D1A1B03933412).

Author details

[1]School of Biological Sciences, College of Natural Sciences, Chungbuk National University, Cheongju, Chungbuk 28644, Republic of Korea. [2]School of Biological Sciences and Technology, Chonnam National University, Gwangju 61186, Republic of Korea. [3]Division of EcoScience, Ewha Womans University, Seoul 03760, Republic of Korea

References

1. Hessling R. Metameric organisation of the nervous system in developmental stages of Urechis caupo (Echiura) and its phylogenetic implications. Zoomorphology. 2002;121 4:221-34. doi:10.1007/s00435-002-0059-7.
2. Hessling R. Novel aspects of the nervous system of Bonellia viridis (Echiura) revealed by the combination of immunohistochemistry, confocal laser-scanning microscopy and three-dimensional reconstruction. In: Sigvaldadóttir E, Mackie ASY, Helgason GV, Reish DJ, Svavarsson J, Steingrímsson SA, et al., editors. Advances in Polychaete Research: Proceedings of the 7th International Polychaete Conference held in Reykjavik, Iceland, 2–6 July 2001. Dordrecht: Springer Netherlands; 2003. p. 225-39.
3. Struck TH, Schult N, Kusen T, Hickman E, Bleidorn C, McHugh D, et al. Annelid phylogeny and the status of Sipuncula and Echiura. Bmc Evolutionary Biology. 2007;7 doi: 10.1186/1471-2148-7-57.
4. Zrzavý J, Říha P, Piálek L and Janouškovec J. Phylogeny of Annelida (Lophotrochozoa): total-evidence analysis of morphology and six genes. BMC Evolutionary Biology. 2009;9 1:189. doi:10.1186/1471-2148-9-189.
5. Struck TH, Paul C, Hill N, Hartmann S, Hosel C, Kube M, et al. Phylogenomic analyses unravel annelid evolution. Nature. 2011;471 7336:95-8. doi:10.1038/nature09864.
6. Andrade SCS, Novo M, Kawauchi GY, Worsaae K, Pleijel F, Giribet G, et al. Articulating "Archiannelids": Phylogenomics and Annelid Relationships, with Emphasis on Meiofaunal Taxa. Molecular Biology and Evolution. 2015;32 11:2860-75. doi:10.1093/molbev/msv157.
7. Anne Weigert and Bleidorn C. Current status of annelid phylogeny. Organisms Diversity & Evolution. 2016;16 2:345-62. doi:10.1007/s13127-016-0265-7.
8. Heyland A, Vue Z, Voolstra CR, Medina M and Moroz LL. Developmental transcriptome of Aplysia californica'. Journal of Experimental Zoology Part B: Molecular and Developmental Evolution. 2011;316B 2:113-34. doi:10.1002/jez.b.21383.
9. Chou H-C, Pruitt MM, Bastin BR and Schneider SQ. A transcriptional blueprint for a spiral-cleaving embryo. BMC Genomics. 2016;17 1:552. doi:10.1186/s12864-016-2860-6.
10. Newby WW. The embryology of the echiuroid worm, Urechis caupo. Philadelphia,: The American Philosophical Society; 1940.
11. Bolger AM, Lohse M and Usadel B. Trimmomatic: a flexible trimmer for Illumina sequence data. Bioinformatics. 2014;30 15:2114-20. doi:10.1093/bioinformatics/btu170.
12. Grabherr MG, Haas BJ, Yassour M, Levin JZ, Thompson DA, Amit I, et al. Full-length transcriptome assembly from RNA-Seq data without a reference genome. Nat Biotech. 2011;29 7:644-52. doi:10.1038/nbt.1883.
13. Fu L, Niu B, Zhu Z, Wu S and Li W. CD-HIT: accelerated for clustering the next-generation sequencing data. Bioinformatics. 2012;28 23:3150-2. doi:10.1093/bioinformatics/bts565.
14. Langmead B, Trapnell C, Pop M and Salzberg SL. Ultrafast and memory-efficient

alignment of short DNA sequences to the human genome. Genome Biology. 2009;10 3:R25. doi:10.1186/gb-2009-10-3-r25.
15. Li B and Dewey CN. RSEM: accurate transcript quantification from RNA-Seq data with or without a reference genome. BMC Bioinformatics. 2011;12 1:323. doi:10.1186/1471-2105-12-323.
16. Simão FA, Waterhouse RM, Ioannidis P, Kriventseva EV and Zdobnov EM. BUSCO: assessing genome assembly and annotation completeness with single-copy orthologs. Bioinformatics. 2015;31 19:3210-2. doi:10.1093/bioinformatics/btv351.
17. Robinson MD and Oshlack A. A scaling normalization method for differential expression analysis of RNA-seq data. Genome Biology. 2010;11 3:R25. doi:10.1186/gb-2010-11-3-r25.
18. Park C, Han YH, Lee SG, Ryu KB, Oh J, Kern EMA, et al. Supporting data for " The developmental transcriptome atlas of the spoon worm Urechis unicinctus (Echiurida: Annelida)". GigaScience Database 2018. http://dx.doi.org/10.5524/100393

Figure Legends

Figure 1. Adult *Urechis unicinctus* used in this study (proboscis retracted). Scale bar; 1cm.

Figure 2. Schematic diagram of *U. unicinctus* transcriptome analysis in this study.

Figure 3. Analysis of *de novo* transcriptome and global gene expression patterns.

(A) Superphylum distribution for homology search of *U. unicinctus* coding sequences against the NR database using the best BLAST hit. (**B)** Results of BUSCO analysis. (**C)** Result of principal component analysis (PCA) and a dendrogram of transcriptomes of 14 *U. unicinctus* developmental stages based on pairwise distance matrices (1 - ρ, Spearman's correlation coefficient). The first, second, and third principal components account for 86.8, 6.8, and 5.9 % of variance, respectively.

Figure 4. Representative images of *U. unicinctus* developmental stages and their gene expression profiles.

(A) Overview of *U. unicinctus* developmental stages. (a) oocyte, (b) fertilized embryo, (c) polar body, (d) 2 cell, (e) 4 cell, (f) 8 cell, (g) 16 cell, (h) 32 cell, (i) blastula, (j) emerged cilia, (k) early trochophore, (l) middle trochophore, (m) late trochophore, (n) segmentation. p, polar body; bp, blastopore; c, cilia; ls, larval stomach; int, intestine; glv, gastro-intestinal valve; m, mouth; vnc, ventral nerve cord; a, anus. Scale bar; 50μm. (**B)** A heat map showing dynamic gene expression patterns with the relative expression levels (column) in each stage (row). Expression values (TMM) were log_2-transformed and mean-centered by transcript. The hierarchical clustering was performed

with Euclidean distances of gene expression values.

 Click here to download Table Han et al-Table 1.docx

Table 1. Reads Statistics

Samples	Total produced bases (bp)	Number of reads	Read length (bp)	GC %	Q30 %	Number of clean reads (%)
Oocyte	8,749,299,078	57,942,378	151	43.87	90.53	54,583,372 (94.20)
Fertilized embryo	7,204,375,496	47,711,096	151	43.86	92.32	45,817,358 (96.04)
Polar body	7,553,516,790	50,023,290	151	41.40	91.12	47,401,970 (94.76)
2 cell	8,663,957,200	57,377,200	151	40.21	92.63	55,263,572 (96.32)
4 cell	6,693,881,642	44,330,342	151	40.88	90.81	43,001,172 (97.00)
8 cell	7,417,271,000	49,121,000	151	42.14	92.31	46,360,492 (94.38)
16 cell	7,993,095,608	52,934,408	151	41.52	91.75	50,571,562 (95.54)
32 cell	22,163,185,664	146,776,064	151	42.11	91.44	139,587,140 (95.10)
Blastula	8,885,042,038	58,841,338	151	45.23	92.04	56,298,300 (95.68)
Emerged cilia	8,077,246,398	53,491,698	151	44.18	89.83	50,401,516 (94.22)
Early trochophore	7,354,720,616	72,819,016	101	45.90	96.02	72,513,798 (99.58)
Middle trochophore	7,581,052,122	75,059,922	101	46.58	96.31	74,755,084 (99.59)
Late trochophore	7,807,192,940	77,298,940	101	46.69	96.66	77,100,204 (99.74)
Segmentation	10,556,984,102	69,913,802	151	48.19	92.37	67,990,654 (97.25)

 Click here to download Table Han et al-Revised Table 2.docx

Table 2. Statistics for *Urechis unicinctus* transcriptome assembly

Samples	Total assembled bases (bp)	Number of assembled transcripts	N50 transcript length (bp) (min - max : median)	Number of non-redundant ORFs	Number of ORFs with NR blast hit (longest ORF per unigene)
Oocyte	45,868,755	26,569	2,801 (201 - 26,298 : 1,105)	9,684	7,791
Fertilized embryo	43,996,849	28,361	2,689 (201 - 26,298 : 917)	9,469	7,561
Polar body	43,132,738	26,716	2,626 (201 - 26,298 : 1,020)	9,246	7,380
2 cell	44,839,836	31,326	2,412 (201 - 26,298 : 917)	9,139	7,254
4 cell	47,675,420	23,122	3,204 (201 - 26,298 : 841)	9,414	7,567
8 cell	45,215,462	27,532	2,564 (201 - 31,183: 1,442)	9,030	7,220
16 cell	49,536,401	33,776	2,470 (201 - 26,298 : 871)	9,470	7,463
32 cell	58,598,783	38,718	2,461 (201 - 26,298 : 927)	11,193	8,597
Blastula	50,083,677	30,553	3,004 (201 - 31,183: 901)	10,994	8,535
Emerged cilia	58,462,746	27,855	3,320 (201 - 31,183: 1,513)	12,153	9,625
Early trochophore	64,464,321	38,443	3,291 (201 - 36,191: 858)	12,980	10,034
Middle trochophore	72,767,170	42,797	3,234 (201 - 36,191: 930)	14,482	11,001
Late trochophore	77,723,477	48,553	3,081 (201 - 36,191: 837)	15,208	11,300
Segmentation	49,350,938	26,509	2,740 (201 - 32,619: 1,318)	11,883	9,030
Total	368,166,154	620,490	846 (201 - 36,191 : 322)	32,880	20,305

 Click here to download Figure Figure1.jpg

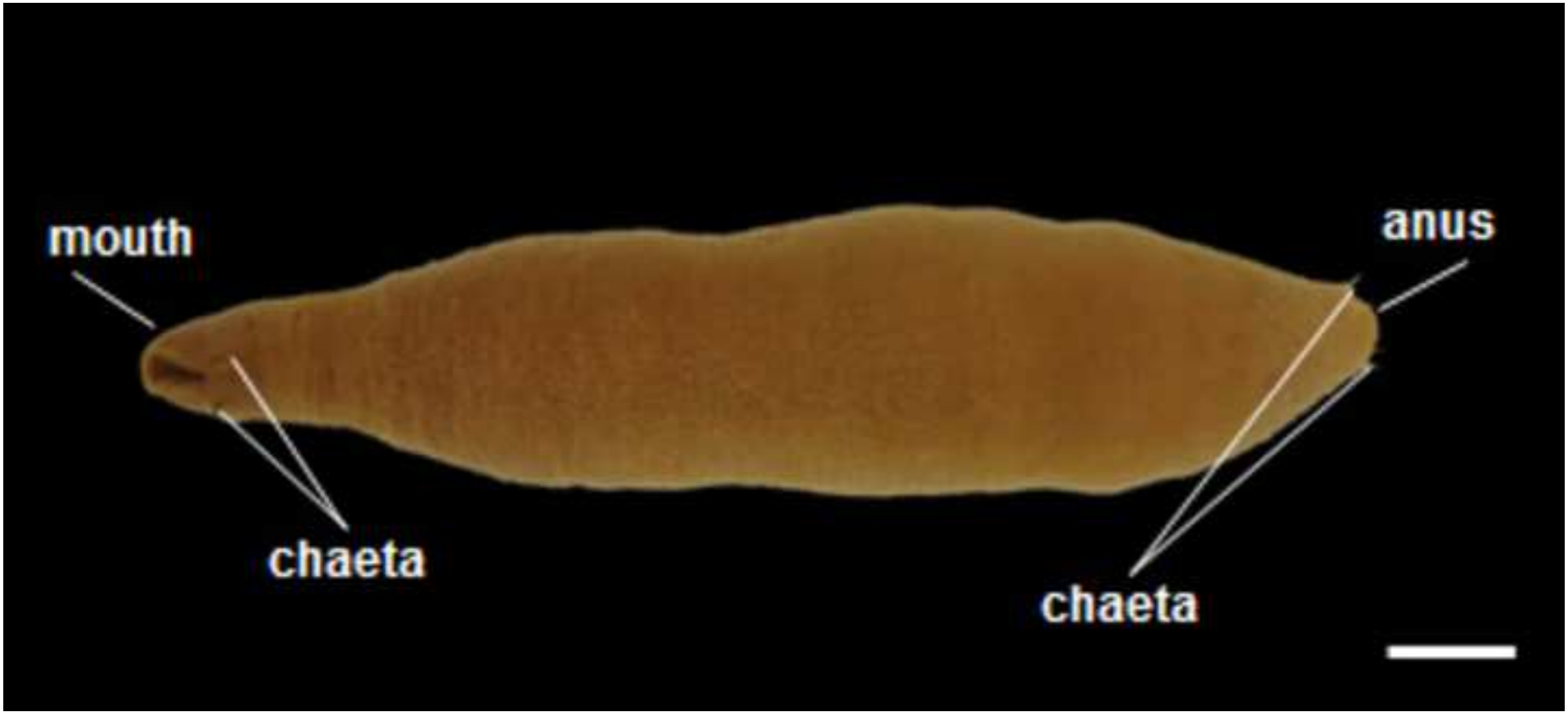

Figure2

Click here to download Figure Revised Figure2.tiff

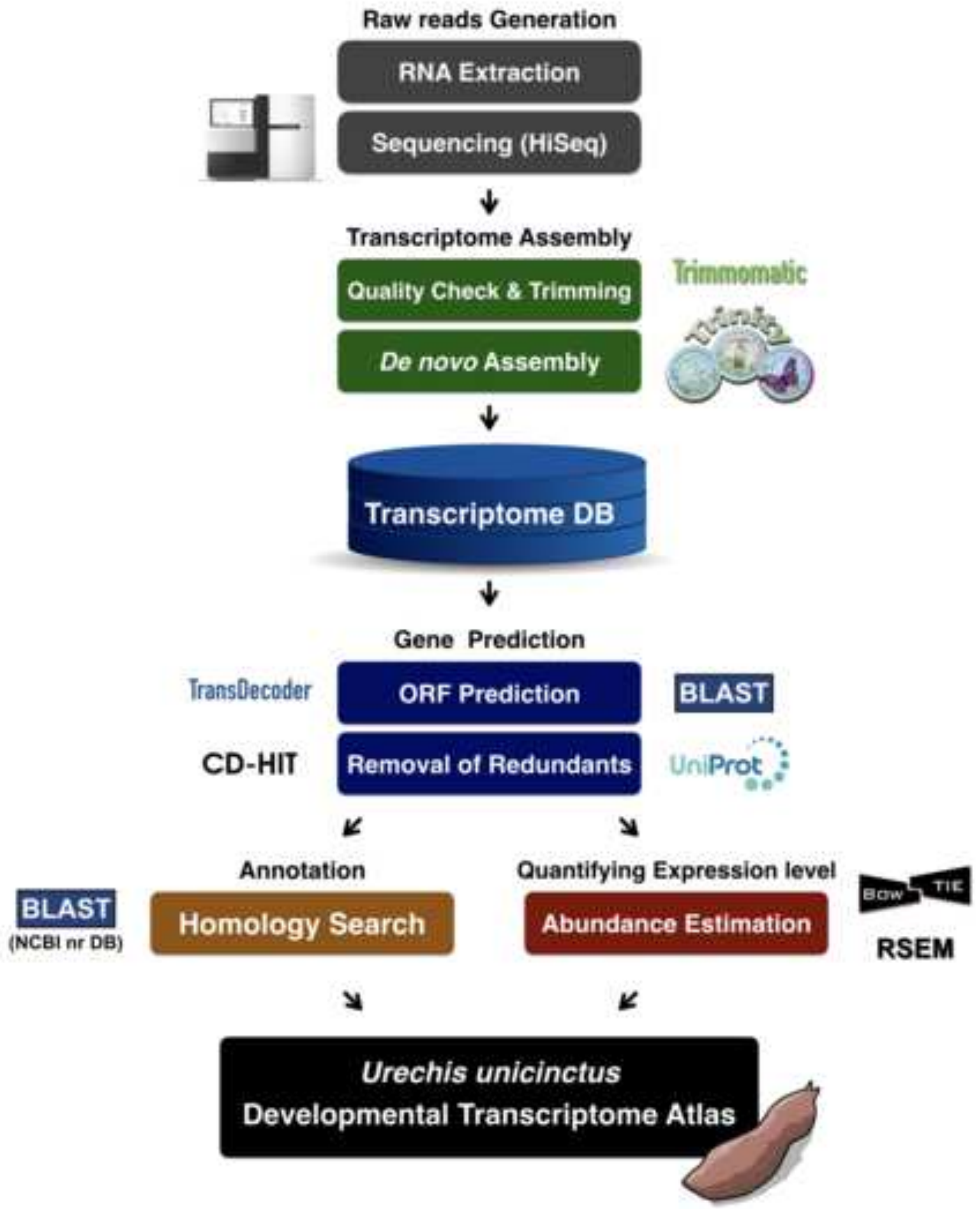

Fig. 2

Figure3 Click here to download Figure Revised Figure3.tiff

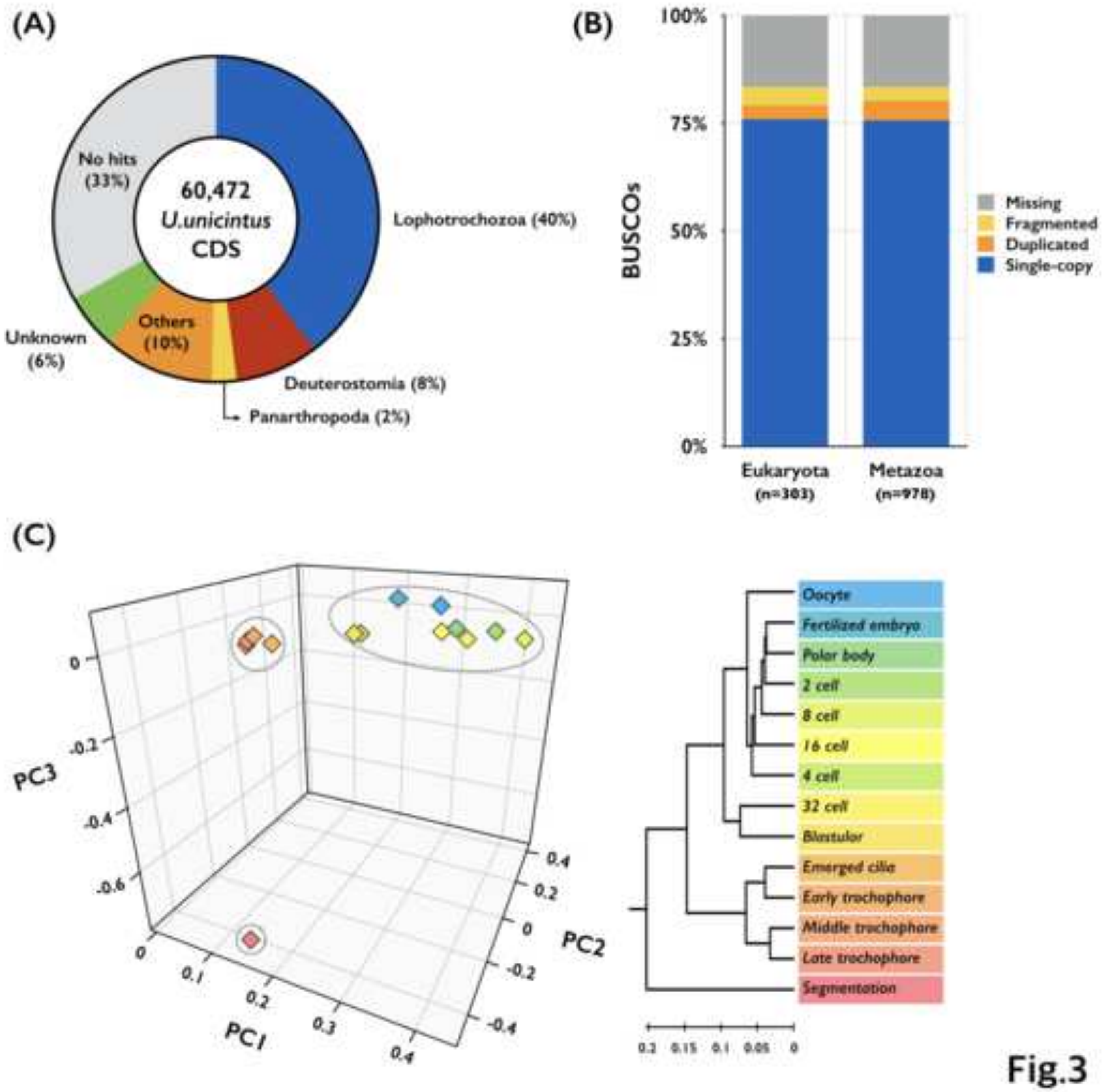

Fig.3

Figure4 Click here to download Figure Revised Figure4.tif

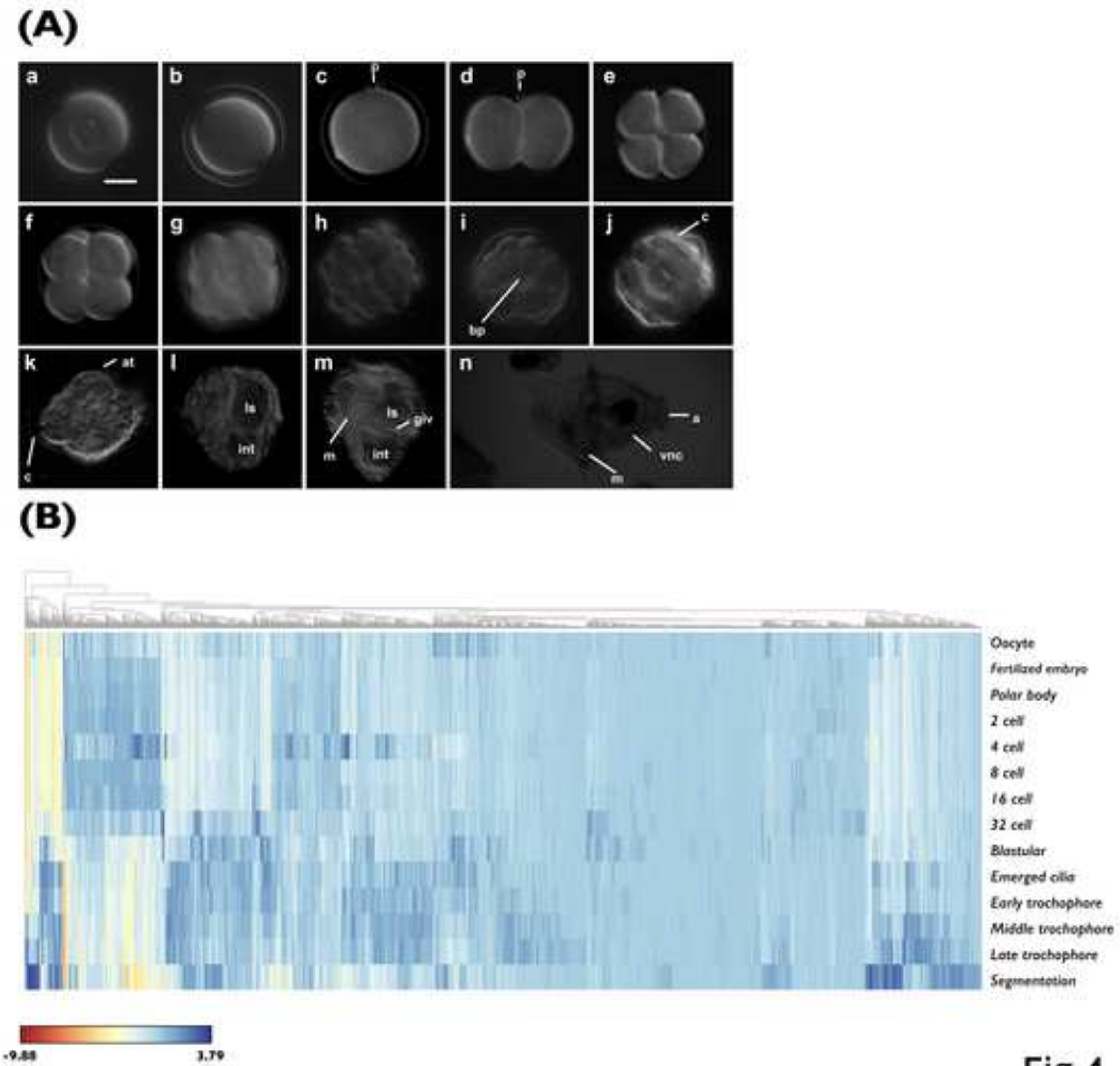

Fig.4
