# Supplementary material for: The developmental transcriptome atlas of the spoon worm Urechis unicinctus (Echiurida: Annelida)
Source: Gigascience. 2018 Feb 15;7(3):giy007. doi: 10.1093/gigascience/giy007 (PMC5863216; doi:10.1093/gigascience/giy007)
Supplement: Response_to_Reviewer_Comments_Original_Submission.pdf [file giy007_response_to_reviewer_comments_original_submission.pdf]

Author's Response To Reviewer Comments

Editor in Chief
GigaScience

RE: Ms. Ref. No.: GIGA-D-17-00202

Dear Editor:
Thank you very much for your letter on September 21, 2017.
Our manuscript (Ms. Ref. No.: GIGA-D-17-00202) entitled “The developmental transcriptome atlas of the spoon worm Urechis unicinctus (Echiurida: Annelida) ” has now been revised based on feedback from the reviewers and editor. With our resubmission we are including a detailed response to each of the reviewers' comments.
I hope all corrections and revisions in the revised manuscript will be satisfactory.

Sincerely yours,

Sung-Jin Cho, Ph.D.
Associate Professor
Department of Biology (S1-5, 204b)
College of Natural Sciences
Chungbuk National University
52 Naesudong-ro, Heungdeok-gu
Cheongju, Chungbuk 361-763
Republic of Korea
E-mail: sjchobio@chungbuk.ac.kr

Reviewer #1: In this study Han and colleagues report the early developmental transcriptome of the spoon worm Urechis unicinctus. This is a valuable resource that certainly will be used by the comparative genomics community and those working on annelid larval development.

I have a few comments to improve the manuscript:

Comment 1:
1) The authors seem to estimate the genome size of Urechis unicinctus based on k-mer analysis. However, for this they would need to use genomic reads, and not transcriptome reads. This estimate should be deleted, unless it was done on genomic sequences, in which case more detail should be given.

Authors’ response:
Although we utilized genomic reads to estimate the genome size of U. unicinctus, we deleted this section because reviewer #2 (comment 2; “It is also not directly relevant to a transcriptomic publication, as genome size is not necessarily reflective of transcriptomic complexity”) also pointed out same issue.

Comment 2:

2)The authors should compare their developmental transcriptome to other studies within (at least) the annelida or the Lophotrochozoa. Placing this study into context by listing other available resources would be valuable for the readers.

Authors' response:
We added one sentence on page 4 (lines13-16) with related references:
"Indeed, data from recently published developmental transcriptomes of other Lophotrochazoans (e.g., Aplysia californica and Platynereis dumerilii) have highlighted insights into molecular mechanisms underlying early development and metamorphosis [6, 7].

Comment 3:
3)The study focuses on larval stages, up to the segmented larva. However, in the abstract the authors write that their resource will be of 'substantial value for understanding how gene repertoires are involved in early stages of development, ontogenic morphogenesis and the formation of an unsegmented body plan." Since no data are reported for later, unsegmented stages, the value of this resource to directly address that problem (i.e. how the unsegmented body forms from a segmented body) is limited. This sentence should be rewritten or data from unsegmented stages should also be included.
Authors' response:
As suggested by the reviewer #1, the sentence has been modified on page 2 (lines 4-7) as following:
"For this reason, transcriptome analyses from various developmental stages of Echiurid can be of substantial value for understanding the precise expression level and the complex regulatory networks during early and larval development."

Reviewer #2: This is an interesting article and a useful dataset, which will be the basis for future work both by the authors and related researchers. However, the text as it stands needs revision to correct some important points. When this is done, it will be a very handy addition to the published record for a variety of researchers.

Major points:
Comment 1:
-replicates have not been performed for timepoints. This should be clearly stated throughout as it is a major limitation of this study. Lines 44-46 of page 2 and lines 46 - 60 on page 9 have major issues, as without replicates these could be outliers, resulting from PCR, assembly or transcriptional artefacts or other problems. It should also be made clear to the reader that without replication, hypotheses of differential expression are tentative at best. Even just double checking these 12,910 "differentially expressed" contigs in particular to ensure that they are actual genes would be useful.

Authors' response:
We agree with the reviewer that biological replicates may contribute to minimizing the number of false positive genes and detecting these differentially expressed genes statistically. However, this limitation can't be fully overcome without the genome sequence. To point out this limitation, we added one sentence on page 10 (lines 15-18) as following:
"Although this study presents the first large-scale developmental transcriptome dataset for a

developmentally interesting animal group, U. unicinctus (Echiurida), its global landscape of developmental transcriptome is not yet complete, due to the lack of biological replicates and reference genome sequences.”

As already described in the manuscript on page 8 (lines 5-11), we applied several bioinformatics analysis methods to detect putative CDS as following:
“After assembly, open reading frames (ORFs) were predicted using TransDecoder (version 3.0.0) (http://transdecoder.sourceforge.net). To maximize sensitivity for capturing ORFs, all transcripts were aligned against the Uniprot/Swiss-Prot database (http://www.uniprot.org) via BLASTP search with an E-value cutoff of 10-5. Next, ORF length < 100 amino acids were discarded to avoid maintaining transcripts with poor evidence for protein-coding regions. Finally, redundant transcripts with more than 99% sequence identity were removed using CD-HIT (version 4.6.5)”

Comment 2:
-Genomic reads are not described or provided. These should be mentioned or cited, as at present Fig 3A is difficult to assess. It is also not directly relevant to a transcriptomic publication, as genome size is not necessarily reflective of transcriptomic complexity.

Authors’ response:
Although we utilized genomic reads to estimate the genome size of U. unicinctus, we deleted these statements because this comment is worthy of consideration.

Comment 3:
-how many adult individuals were used to produce embryos? This will be important to assess allelic diversity.

Authors’ response:
We modified the sentence on page 5 (lines 10-12) as following:
“We extracted eggs and sperm from one adult female and one male. To obtain U. unicinctus embryos, artificial fertilization was performed by mixing the appropriate ratio of sperms and eggs.”

Comment 4:
- Particularly given the feeding of trochophore larvae, it is surprising that no assessment of potential contamination is performed or provided. Please address this, perhaps by performing a basic blastn against the non metazoan (or even just the algal and bacterial) section of the nr database for each timepoint and report the degree of any potential contamination (e.g. matching with high similarity) if it is found. If it is found, BUSCO results etc may need to be checked. It would be useful to also cross-reference the 12910 "differentially expressed" set against this.

Authors’ response:
We excluded sequences derived from non-metazoan taxa and re-performed all further analyses including BUSCO, PCA, “differentially expressed genes”.
Here is the updated version of text.
From Page 8 (line 21) to page 9 (lines 1-14): “To annotate coding sequences (CDS), the resulting

60,472 CDSs were compared against the NCBI non-redundant protein (NR) database (downloaded on April 11, 2017) using BLASTP with an E-value cutoff of 10-10 and the best blast hit. About 66 % (40,111/60,472) of the CDS were assigned to superphylum-level taxa including Lophotrochozoa (40%), Deuterostomia (8%), and Panarthropoda (2%) (Fig. 3A), which is to be generally expected. For further analysis, we excluded a number of CDSs (18%; 7,231/40,111) by using sequences derived from non-metazoan taxa. When there were multiple coding sequences that mapped to the same gene in the NR database, the sequences with the longest CDS were first assigned to that gene. Based on this criterion, we established a comprehensive transcriptome database of 14 early developmental stages of U. unicinctus that comprises 20,305 representative functional protein-coding transcripts. We further assessed the completeness of the U. unicinctus development transcriptome using the program BUSCO (bench-marking universal single-copy orthologs) (version 2.0) [14]. 75.9% (230 / 303 genes), and 75.7% (740 / 978 genes) of the eukaryote and metazoan single-copy orthologs were identified, respectively (Fig. 3B)"

and page 10 (lines 11-13): "We identified a total of 12,705 genes that showed dynamic regulation patterns during the differentiation and maturation of U. unicinctus cells (Fig. 4)."

Comment 5:
-Page 7, lines 1-15: estimates of coverage are misleading at best when cross- comparing genomic and transcriptomic data. Transcriptomes are almost certainly non-uniform in coverage. This section needs to be re-considered.

Authors' response:
Please see response to Comment 2.

Comment 6:
-Fig 2 seems to indicate that "quantifying expression level" (right column) was performed on the transcriptome build before removal of redundancy (left hand column). Is this correct? Or was the quantification performed on the CD-HIT - cured set of transcripts? (and quantifying expression level would then follow after CD-HIT, rather than being independent?)

Authors' response:
We performed the "abundance estimation" after removal of redundants. We redrew the Figure 2.

Minor points: (page numbers as per those 'burned in' at bottom of pages, not pdf page numbers, which have additional review pages)

Comment 7:
-Give an assembly download link in the text (and thanks for making these available for review – this was important)

Authors' response:
We added the BioProject link on page 11 (line 8) in the manuscript as following:
"(https://www.ncbi.nlm.nih.gov/bioproject/?term=PRJNA394029)"

All further data (will be updated because of “comment 4”) have already deposited in the GigaDB, which probably can be provided from Gigascience journal. Here is the list of files we deposited.

1. Urechis_Dev_Draft_Trinity.fasta
(Draft de novo transcriptome assembly using Trinity)
2. A. Urechis_Dev_Removal_Redundant_mRNA.fasta
(mRNA sequences after gene prediction and removing redundant)
B. Urechis_Dev_Removal_Redundant_cds.fasta
(Coding sequences (nucleotide) after gene prediction and removing redundant)
C. Urechis_Dev_Removal_Redundant_prot.fasta
(Coding sequences (amino acid) sequences after gene prediction and removing redundant)
3. A. Urechis_Dev_CDS_M_mRNA.fasta
(mRNA sequences of representative protein coding sequence)
B. Urechis_Dev_CDS_M_cds.fasta
(Coding sequences (nucleotide) of representative protein coding sequence)

C. Urechis_Dev_CDS_M_prot.fasta
(Coding sequences (amino acid) of representative protein coding sequence)
D. Urechis_Dev_CDS_M.gff3
(Annotation of representative protein coding sequence)
4. Urechis_Dev_CDS_M_UPGMA_tree.nwk
(Dendrogram based on pairwise distance matrices (1-rho, Spearman’s correlation coefficient)
5. Urechis_Dev_CDS_M_EXP.tsv
(Expression level (FPKM) of 14 stages)
6. A. Urechis_Dev_CDS_M_BUSCO_eukaryota.zip
(Results of BUSCO analysis using eukaryote single-copy orthologs)
B. Urechis_Dev_CDS_M_BUSCO_metazoa.zip
(Results of BUSCO analysis using metazoan single-copy orthologs)

Comment 8:
pg 2 line 13 - should this be "of echiurid species" (echiurid as an adjective, not a noun?)

Authors’ response:
Corrected.

Comment 9:
pg 2 line 26: average - is this the mean, median? N50 useful here?

Authors’ response:
The meaning of “average” in our manuscript indicates “mean”, but the use of N50 is better than “average length”. We updated this section on page 2 (lines 8-10) as following:
“A total of 914 million raw RNA-Seq reads were produced from 14 developmental stages of Urechis unicinctus, and were de novo assembled into contigs spanning 63,928,225 bp with an

N50 length of 2,700 bp.”

Comment 10:
pg 5 line 13 - "sandy bottom" is an odd phrasing. "Sandy intertidal areas?"

Authors’ response:
It was changed into “intertidal mud flat” on page 5 (line 9).

Comment 11:
-Can any developmental landmarks be given for the early/mid/late trochophore stage, or refer tophotographs?

Authors’ response:
The following sentences and reference on page 5 (lines 20-23) and page 6 (lines 1-2) added into the revised manuscript.
“Diagnostic features for each of the three trochophore stages are as follows. The early trochophore is a non-feeding stage. In the middle trochophore, the gastro-intestinal valve opens and the anus appears. Late trochophore has longer cilia of the apical tufts that are replaced by shorter cilia which cover a greater area and the prototroch cilia are longer. The developmental stages of the U. unicinctus embryos followed Newby 's classification [8].”

Comment 12:
-What artificial seawater was used?

Authors’ response:
We added the sentence on page 5 (lines 13-14) as following:
“The embryos were reared in artificial seawater (Reef crystals (Aquarium Systems, France)) in a plastic case at room temperature (18-20 °C).”

Comment 13:
pg 6 line 10 mRNA (not the mRNAs)

Authors’ response:
Corrected.

Comment 14:
pg 6 line 20 how many rounds of PCR in library construction? What adapters used?

Authors’ response:
We added and modified a couple of sentences on page 6 (lines 12-22) and page 7 (lines 1-7) as following:
“Total RNA concentration was calculated by Quant-IT RiboGreen (Invitrogen, #R11490). To assess the integrity of the total RNA, samples are run on the TapeStation RNA screentape (Agilent, #5067-5576). Only high-quality RNA preparations, with RIN greater than 7.0, were used for RNA library construction. A library was independently prepared with 1ugg of total RNA for each sample by Illumina TruSeq Stranded Total RNA Sample Prep Kit (Illumina, Inc.,

San Diego, CA, USA). The rRNA in total RNA is depleted by Ribo-Zero kit. After the rRNA is depleted, the remaining RNA is purified, fragmented and primed for cDNA synthesis. The cleaved RNA fragments are copied into first strand cDNA using reverse transcriptase and random hexamers. This is followed by second strand cDNA synthesis using DNA Polymerase I, RNase H and dUTP. These cDNA fragments then go through an end repair process, the addition of a single 'A' base, and then ligation of the adapters. The products are then purified and enriched with PCR to create the final cDNA library. The libraries were quantified using qPCR according to the qPCR Quantification Protocol Guide (KAPA Library Quantification kits for Illumina Sequencing platforms) and qualified using the TapeStation D1000 ScreenTape (Agilent Technologies, Waldbronn, Germany). Indexed libraries were then submitted to a Illumina Hiseq 4000 (Illumina, Inc., San Diego, CA, USA), and the paired-end (2×100 bp) sequencing was performed."

Comment 15:
pg 8 line 22, and Fig 3B. Is this best blast hits, please report this in the figure legend and text if so.

Authors' response:
Yes, it is the best blast hit. We updated this section and figure legend on (1) page 8 (lines 21-22), page 9 (line 1), and (2) page 15 (lines 6-7) as following:
(1) "To annotate coding sequences (CDS), the resulting 60,472 CDSs were compared against the NCBI non-redundant protein (NR) database (downloaded on April 11, 2017) using BLASTP with an E-value cutoff of 10-10 and the best blast hit."
(2) "Superphylum distribution for homology search of U. unicinctus coding sequences against the NR database using the best blast hit."

Comment 16:
Page 8 lines 48 - 53: This seems a deeply flawed comparison, and as you acknowledge, compares very non-comparable things. Maybe find recent transcriptomes that have used BUSCO as an assessment tool and use these as exemplars? All of these were published before BUSCO.

Authors' response:
Without a reference genome, highly accurate whole transcriptome assembly from short reads will be a tough challenge. So far, there have been no reports of the completeness assessment of the de novo transcriptome assemblies using the BUSCO scores. Therefore, we deleted these sentences in our revised manuscript.

Comment 17:
-page 9 line 47: 10 fold (total)? or is this a log2(a/b) measure? The FDR also seems to be communicated oddly. Do you mean a p-value cutoff for FDR of 0.001?

Authors' response:
The fold value is 10-fold, and the score 0.001 is for FDR cut-off but not for p-value.
To clarify and more fully explain its meaning, we modified the sentence on page 10 (lines 9-11) as following:
"In an additional analysis, a gene whose expression level was 0.1%) in at≤ 10-fold and FDR

adjusted P value ≥significantly changed (least one comparison, was defined as a developmentally regulated gene.”

Comment 18:
Pg 13 line 5: I think it better to say "Adult Urechis unicinctus" (no "worm of)

Authors’ response:
Corrected.

Comment 19:
Line 44: scale spelled "sclae"

Authors’ response:
Corrected.

Comment 20:
In several locations and in data - I understand the use of FKPM, but TPM may be a more useful measure - it allows the cross - comparison of these results between samples, while FKPM does not.Consider providing this information if you have it.

Authors’ response:
We agree, and investigated whether there is difference in expression profiles based on between TPM and FPKM values. In all 14 samples, the expression level values between TPM and FPKM are highly coupled (see the below table), indicating that our results could not be influenced by gene expression measurements.

P<2.2e-16 with all comparisons
TPM vs FPKM
stage Spearman'ss (p)
oocytes 0.9998206
fertilized egg 0.9998886
polar body 0.9999137
2 cell 0.9998949
4 cell 0.9998529
8 cell 0.9999356
16 cell 0.9998927
32 cell 0.999884
blastular 0.9999695
emerged cilia 0.9999853
early trochophore 0.999974
middle trochophore 0.9999774
late trochophore 0.9999885
segmentation 0.9999447

Comment 21:

- the references are in an odd font, which may require changing depending on journal preferences.

Authors' response:
Corrected.

Reviewer #3:
Comment 1:
The paper presents transcriptomic libraries for 14 developmental stages of an echiurid species. This is within the scope of the journal. Hence, I suggest accepting the paper after a major revision. My major concern is the introduction. The introduction does not adequately reflect the literature on the position of Echiura. With respect to morphological data the studies of Hessling concerning the nervous system are missing. This among others also reflected in the erroneous statement that the presence of a larval nervous system includes placement in Annelida. However, this is in this way not correct. Larval nervous systems occur throughout the animal kingdom, it is the specific features of this system, which support this placement and require citing the works by Hessling. Moreover, recent studies using phylogenomic data concerning the phylogeny of Annelida are also not mentioned (see below). Finally, for understanding the early evolution of Lophotrochozoa Echiura are irrelevant as they are highly derived annelid taxa. Echiura are more interesting in understanding the plasticity of body plan evolution in general, but this independent of their position within Lophotrochozoa. Moreover, citations supporting the claim that Urechis unicinctus attracted special attention for evo-devo are lacking and should be provided.

Authors' response:
We appreciate the reviewer's feedback and agree with the suggested changes. In the introduction, we have now corrected the incorrect statement about the larval nervous system and have added references to Hessling's work. We have also added references to the recent phylogenomic research on the placement of Echiurids within Annelida. We also have changed the sentence about evo-devo to emphasize instead the potential use of Urechis to understand the evolutionary loss of adult segmentation. Finally, we made all corrections according to your minor comments (see all changes below).
Here is the updated text on page 4 (lines 3-21):
"Within the major annelid groups, Echiurida (also called the 'marine spoon worms') is represented by a morphologically and ontogenetically unique assemblage that includes approximately 165 species, most of which lacks segmentation in adults, although they possess annelid-like morphological and developmental features including the organization of the larval nervous system [1]. They were once considered a separate metazoan phylum, but reevaluation of morphological and molecular data indicated that Echiurida is nested within the Annelida representing one of three animal phyla with body segmentation [1-5]. In this respect, transcriptome analyses from various developmental stages of Echiurida are of substantial value for understanding precise expression levels and the complex regulatory networks involved in early and larval development. Indeed, data from recently published developmental transcriptomes of other Lophotrochazoans (e.g., Aplysia californica and Platynereis dumerilii) have highlighted insights into molecular mechanisms underlying early development and metamorphosis [6, 7].
Urechis unicinctus is an Echiuran species that inhabits burrows in intertidal soft sediments (Fig.

1). The Urechis genus may hold important clues to the genetic basis of the evolutionary gain and loss of segmentation, due to its nested position within Annelida (i.e., sister to capitellid polychaetes), a Lophotrochozoan phylum that is represented by a diverse group of segmented worms [2, 5].”

Reviewer #3’s minor comments:
Comment 2:
Page 2, Line 6: delete "the phylum"

Authors’ response:
Authors want to keep “the phylum” in its original place.

Comment 3:
Page 2, Line 8: change to "unlike most other annelid"

Authors’ response:
changed

Comment 4:
Page 2, Line 16: insert space between "development, ontogenetic"

Authors’ response:
corrected

Comment 5:
Page 2, Line 57: insert dot between "stages. These"

Authors’ response:
corrected

Comment 6:
Page 4, Line 36: delete dot between "(Fig. 1). This"

Authors’ response:
modified

Comment 7:
Page 4, Line 48: delete "the" at the end of the line

Authors’ response:
deleted

Comment 8:
Page 4, Line 51: "profiles" instead of "profiling"

Authors’ response:

changed

Comment 9:
Page 7, Line 2: describe how the k-mer analysis was conducted in detail

Authors' response:
We deleted this section because review #1 (comment 1; "The authors seem to estimate the genome size of Urechis unicinctus based on k-mer analysis. However, for this they would need to use genomic reads, and not transcriptome reads. This estimate should be deleted, unless it was done on genomic sequences, in which case more detail should be given") and reviewer #2 (comment 2; "It is also not directly relevant to a transcriptomic publication, as genome size is not necessarily reflective of transcriptomic complexity") also pointed out same issue.

Comment 10:
Page 7, Lines 18-25: I do not understand what has been done here. Concatenation means that individual sequences are attached to each other one after the other, but I think this is not meant here. Rather assume that the reads from all 14 libraries were pooled together for assembly of the data. Please clarify this point.

Authors' response:
We modified "concatenated" into "pooled".

Comment 11:
Page 8, Line 7: How did you correct the expression profiles for the uneven sequencing depth among the different libraries? The range is from 43 million reads to 140 million and hence the latter is more than three times higher.

Authors' response:
The FPKM value we used in our analysis is quantifying RNA-seq data by normalizing (1) read length and (2) the number of reads sequenced. According to the latter case, the expression profiles among libraries with uneven sequencing depth have been already normalized in order to compare expression levels of genes among these libraries.

Comment 12:
Page 8, Line 46: include a comma in "were identified,"

Authors' response:
corrected

Comment 13:
Page 13, Line 24: This is not a phylogenetic tree, which by definition can only be reconstructed between species and not developmental stages between individuals. Change wording.

Authors' response:
This sentence on page 15 (lines 8-10) was changed as following:
"Result of principal component analysis (PCA) and a dendrogram of transcriptomes of 14 U.

unicinctus developmental stages based on , Spearman’s correlation coefficient).”ρpairwise distance matrices (1 -

Comment 14:
Page 13, Line 44: "mouth" instead of "mouse"

Authors’ response:
corrected

Comment 15:
Page 13, Line 44: insert a space between "50μm. (B)"

Authors’ response:
a space is inserted

Comment 16:
Citations missing:
Hessling, R. & Westheide, W. (2002) Are Echiura derived from a segmented ancestor? Immunohistochemical analysis of the nervous system in developmental stages of Bonellia viridis.
Journal of Morphology, 252, 100-113.
Hessling, R. (2002) Metameric organisation of the nervous system in developmental stages of Urechis
caupo (Echiura) and its phylogenetic implications. Zoomorphology, 121, 221-234.
Hessling, R. (2003) Novel aspects of the nervous system of Bonellia viridis (Echiura) revealed by
the combination of immunohistochemistry, confocal laser-scanning microscopy and three-dimensional
reconstruction. Hydrobiologia, 496, 225-239.
Struck, T.H., Paul, C., Hill, N., Hartmann, S., Hösel, C., Kube, M., Lieb, B., Meyer, A., Tiedemann, R., Purschke, G. & Bleidorn, C. (2011) Phylogenomic analyses unravel annelid evolution.
Nature, 471, 95-98.
Weigert, A., Helm, C., Meyer, M., Nickel, B., Arendt, D., Hausdorf, B., Santos, S.R., Halanych, K.M., Purschke, G., Bleidorn, C. & Struck, T.H. (2014) Illuminating the base of the annelid tree using transcriptomics. Molecular Biology and Evolution, 31, 1391-1401.
Struck, T.H., Golombek, A., Weigert, A., Franke, Franziska A., Westheide, W., Purschke, G., Bleidorn, C. & Halanych, K.M. (2015) The Evolution of Annelids Reveals Two Adaptive Routes to the
Interstitial Realm. Current Biology, 25, 1993-1999.
Andrade, S.C.S., Novo, M., Kawauchi, G.Y., Worsaae, K., Pleijel, F., Giribet, G. & Rouse, G.W. (2015) Articulating "archiannelids": Phylogenomics and annelid relationships, with emphasis on meiofaunal taxa. Molecular Biology and Evolution.

Authors’ response:
Added.
