# Supplementary material for: The developmental transcriptome atlas of the spoon worm Urechis unicinctus (Echiurida: Annelida)
Source: Gigascience. 2018 Feb 15;7(3):giy007. doi: 10.1093/gigascience/giy007 (PMC5863216; doi:10.1093/gigascience/giy007)
Supplement: Response_to_Reviewer_Comments_Revision_1.pdf [file giy007_response_to_reviewer_comments_revision_1.pdf]

Author's Response To Reviewer Comments

Reviewer reports:
Reviewer #2:
Revisions have been made as appropriate.

There are a few minor spelling errors (e.g. "Lophotrochazoans" pg 4 line 14) and for Fig 3A it would be good to define what is meant by N/A (c.f. "Others" and "No hits") that should be corrected in proof.
Authors' response:
As suggested by the reviewer#2, we corrected " Lophotrochozoans" instead of " Lophotrochazoans ".
"N/A" indicate the species group without assigned superphylum-level. To clarify this point, it has been changed to "Unknown" and updated the Figure 3A.

Reviewer #3: The manuscript has improved, but still some points need to be addressed:
1.) The authors did not include all the citations I asked to add. If this is due to a citation limit not stated in the "Instruction to Authors" the authors should exclude Struck et al. (2011) and Andrade et al. (2015) as the review by Weigert and Bleidorn cover the recent phylogenomic studies on annelid phylogeny and instead include the two Hessling (2002) papers as they present the results on the nervous system.
Authors' response:
We have added references of Hessling's works (see reference no. 1 and 2).

Reviewer #3:
2.) Linnean categories like phylum have no biological meaning and are just an artificial categorical level. Therefore, their usage should be restricted to the least possible and whenever possible neutral should be used. Hence, the following changes should be made:
Page 2, Line 2: delete "the phylum"
Page 4, Line 8: "taxon" instead of "phylum"
Page 4, Line 9: "the three major animal taxa" instead of "the three animal phyla"
Page 4, Line 20: "taxon" instead of "phylum"
Page 10, Line 22: "annelid" instead of "phylum"
Authors' response:
As suggested by the reviewer#3, we corrected "the phylum (Page 2, Line 2)" and "annelid" instead of "phylum (Page 10, Line 20)"
Authors hope to keep "the phylum (Page 4, Line 7; Page 4, Line 9; Page 4, Line 20)" in its original place as taxonomic category of Linnean system often provides some information related to the history of its systematic position.

Reviewer #3:
3.) Minor changes:
Page 4, Line 9: delete "the" before Annelida (Annelida is grammatically a proper name and does not need a definitive article)

Authors’ response:
Deleted.

Reviewer #3:
Page 7, Lines 6-10: The two sentences are confusing. It sounds like you did one round of sequencing and then another round on the same material. Please rephrase.
Authors’ response:
The first sentence was removed.

Reviewer #3:
Page 9, Line 19: "3C" instead of "3D"
Page 15, Figure legend 3: The description for Figure 3D is lacking.
Authors’ response:
Updated Figure 3 is composed of three panels (3A, 3B, and 3C). Figure 3C is two results (PCA and dendrogram) because they both indicate the transcriptome comparison with different visualization methods.
