# Supplementary material for: The developmental transcriptome atlas of the spoon worm Urechis unicinctus (Echiurida: Annelida)
Source: Gigascience. 2018 Feb 15;7(3):giy007. doi: 10.1093/gigascience/giy007 (PMC5863216; doi:10.1093/gigascience/giy007)
Supplement: Reviewer_1_Report_(Original_Submission) -- Gaspar Jekely — 06 Sep 2017 Reviewed [file giy007_reviewer_1_report_(original_submission).pdf]

Reviewer Report

Title: The developmental transcriptome atlas of the spoon worm Urechis unicinctus (Echiurida: Annelida)

Version: Original Submission **Date:** 06 Sep 2017

Reviewer name: Gaspar Jekely

Reviewer Comments to Author:

In this study Han and colleagues report the early developmental transcriptome of the spoon worm Urechis unicinctus. This is a valuable resource that certainly will be used by the comparative genomics community and those working on annelid larval development.

I have a few comments to improve the manuscript:

1) The authors seem to estimate the genome size of Urechis unicinctus based on k-mer analysis. However, for this they would need to use genomic reads, and not transcriptome reads. This estimate should be deleted, unless it was done on genomic sequences, in which case more detail should be given.

2) The authors should compare their developmental transcriptome to other studies within (at least) the annelida or the Lophotrochozoa. Placing this study into context by listing other available resources would be valuable for the readers.

3) The study focuses on larval stages, up to the segmented larva. However, in the abstract the authors write that their resource will be of 'substantial value for understanding how gene repertoires are involved in early stages of development, ontogenic morphogenesis and the formation of an unsegmented body plan." Since no data are reported for later, unsegmented stages, the value of this resource to directly address that problem (i.e. how the unsegmented body forms from a segmented body) is limited. This sentence should be rewritten or data from unsegmented stages should also be included.

Level of Interest

Please indicate how interesting you found the manuscript: An article whose findings are important to those with closely related research interests

Quality of Written English

Please indicate the quality of language in the manuscript: Acceptable

Declaration of Competing Interests

Please complete a declaration of competing interests, considering the following questions:

- Have you in the past five years received reimbursements, fees, funding, or salary from an organisation that may in any way gain or lose financially from the publication of this manuscript, either now or in the future?
- Do you hold any stocks or shares in an organisation that may in any way gain or lose financially from the publication of this manuscript, either now or in the future?
- Do you hold or are you currently applying for any patents relating to the content of the manuscript?
- Have you received reimbursements, fees, funding, or salary from an organization that holds or has applied for patents relating to the content of the manuscript?
- Do you have any other financial competing interests?
- Do you have any non-financial competing interests in relation to this paper?

If you can answer no to all of the above, write 'I declare that I have no competing interests' below. If your reply is yes to any, please give details below.

I declare that I have no competing interests

I agree to the open peer review policy of the journal. I understand that my name will be included on my report to the authors and, if the manuscript is accepted for publication, my named report including any attachments I upload will be posted on the website along with the authors' responses. I agree for my report to be made available under an Open Access Creative Commons CC-BY license (http://creativecommons.org/licenses/by/4.0/). I understand that any comments which I do not wish to be included in my named report can be included as confidential comments to the editors, which will not be published.

I agree to the open peer review policy of the journal

To further support our reviewers, we have joined with Publons, where you can gain additional credit to further highlight your hard work (see: https://publons.com/journal/530/gigascience). On publication of this paper, your review will be automatically added to Publons, you can then choose whether or not to claim your Publons credit. I understand this statement.

Yes
