# Supplementary material for: The developmental transcriptome atlas of the spoon worm Urechis unicinctus (Echiurida: Annelida)
Source: Gigascience. 2018 Feb 15;7(3):giy007. doi: 10.1093/gigascience/giy007 (PMC5863216; doi:10.1093/gigascience/giy007)
Supplement: Reviewer_2_Report_(Original_Submission) -- Nathan Kenny — 11 Sep 2017 Reviewed [file giy007_reviewer_2_report_(original_submission).pdf]

Reviewer Report

Title: The developmental transcriptome atlas of the spoon worm Urechis unicinctus (Echiurida: Annelida)

Version: Original Submission **Date:** 11 Sep 2017

Reviewer name: Nathan Kenny

Reviewer Comments to Author:

This is an interesting article and a useful dataset, which will be the basis for future work both by the authors and related researchers. However, the text as it stands needs revision to correct some important points. When this is done, it will be a very handy addition to the published record for a variety of researchers.

Major points:

-replicates have not been performed for timepoints. This should be clearly stated throughout as it is a major limitation of this study. Lines 44-46 of page 2 and lines 46 - 60 on page 9 have major issues, as without replicates these could be outliers, resulting from PCR, assembly or transcriptional artefacts or other problems. It should also be made clear to the reader that without replication, hypotheses of differential expression are tentative at best. Even just double checking these 12,910 "differentially expressed" contigs in particular to ensure that they are actual genes would be useful.

-Genomic reads are not described or provided. These should be mentioned or cited, as at present Fig 3A is difficult to assess. It is also not directly relevant to a transcriptomic publication, as genome size is not necessarily reflective of transcriptomic complexity.

-how many adult individuals were used to produce embryos? This will be important to assess allelic diversity.

- Particularly given the feeding of trochophore larvae, it is surprising that no assessment of potential contamination is performed or provided. Please address this, perhaps by performing a basic blastn against the non metazoan (or even just the algal and bacterial) section of the nr database for each timepoint and report the degree of any potential contamination (e.g. matching with high similarity) if it is found. If it is found, BUSCO results etc may need to be checked. It would be useful to also cross-reference the 12910 "differentially expressed" set against this.

-Page 7, lines 1-15: estimates of coverage are misleading at best when cross- comparing genomic and transcriptomic data. Transcriptomes are almost certainly non-uniform in coverage. This section needs to be re-considered.

-Fig 2 seems to indicate that "quantifying expression level" (right column) was performed on the transcriptome build before removal of redundancy (left hand column). Is this correct? Or was the quantification performed on the CD-HIT - cured set of transcripts? (and quantifying expression level would then follow after CD-HIT, rather than being independent?)

Minor points: (page numbers as per those 'burned in' at bottom of pages, not pdf page numbers, which have additional review pages)

-Give an assembly download link in the text (and thanks for making these available for review - this was important)

pg 2 line 13 - should this be "of echiurid species" (echiurid as an adjective, not a noun?)

pg 2 line 26: average - is this the mean, median? N50 useful here?

pg 5 line 13 - "sandy bottom" is an odd phrasing. "Sandy intertidal areas?"

-Can any developmental landmarks be given for the early/mid/late trochophore stage, or refer to photographs?

-What artificial seawater was used?

pg 6 line 10 mRNA (not the mRNAs)

pg 6 line 20 how many rounds of PCR in library construction? What adapters used?

pg 8 line 22, and Fig 3B. Is this best blast hits, please report this in the figure legend and text if so.

Page 8 lines 48 - 53: This seems a deeply flawed comparison, and as you acknowledge, compares very non-comparable things. Maybe find recent transcriptomes that have used BUSCO as an assessment tool and use these as exemplars? All of these were published before BUSCO.

-page 9 line 47: 10 fold (total)? or is this a log2(a/b) measure? The FDR also seems to be communicated oddly. Do you mean a p-value cutoff for FDR of 0.001?

Pg 13 line 5: I think it better to say "Adult Urechis unicinctus" (no "worm of)
Line 44: scale spelled "sclae"

In several locations and in data - I understand the use of FKPM, but TPM may be a more useful measure - it allows the cross - comparison of these results between samples, while FKPM does not. Consider providing this information if you have it.

- the references are in an odd font, which may require changing depending on journal preferences.

Level of Interest

Please indicate how interesting you found the manuscript: An article whose findings are important to those with closely related research interests

Quality of Written English

Please indicate the quality of language in the manuscript: Acceptable

Declaration of Competing Interests

Please complete a declaration of competing interests, considering the following questions:

- Have you in the past five years received reimbursements, fees, funding, or salary from an organisation that may in any way gain or lose financially from the publication of this manuscript, either now or in the future?
- Do you hold any stocks or shares in an organisation that may in any way gain or lose financially from the publication of this manuscript, either now or in the future?
- Do you hold or are you currently applying for any patents relating to the content of the manuscript?
- Have you received reimbursements, fees, funding, or salary from an organization that holds or has applied for patents relating to the content of the manuscript?
- Do you have any other financial competing interests?
- Do you have any non-financial competing interests in relation to this paper?

If you can answer no to all of the above, write 'I declare that I have no competing interests' below. If your reply is yes to any, please give details below.

I declare that I have no competing interests

I agree to the open peer review policy of the journal. I understand that my name will be included on my report to the authors and, if the manuscript is accepted for publication, my named report including any attachments I upload will be posted on the website along with the authors' responses. I agree for my report to be made available under an Open Access Creative Commons CC-BY license (http://creativecommons.org/licenses/by/4.0/). I understand that any comments which I do not wish to

be included in my named report can be included as confidential comments to the editors, which will not be published.

I agree to the open peer review policy of the journal

To further support our reviewers, we have joined with Publons, where you can gain additional credit to further highlight your hard work (see: https://publons.com/journal/530/gigascience). On publication of this paper, your review will be automatically added to Publons, you can then choose whether or not to claim your Publons credit. I understand this statement.

Yes
