# Supplementary material for: The developmental transcriptome atlas of the spoon worm Urechis unicinctus (Echiurida: Annelida)
Source: Gigascience. 2018 Feb 15;7(3):giy007. doi: 10.1093/gigascience/giy007 (PMC5863216; doi:10.1093/gigascience/giy007)
Supplement: Reviewer_3_Report_(Original_Submission) -- Torsten Struck — 20 Sep 2017 Reviewed [file giy007_reviewer_3_report_(original_submission).pdf]

Reviewer Report

Title: The developmental transcriptome atlas of the spoon worm Urechis unicinctus (Echiurida: Annelida)

Version: Original Submission **Date:** 20 Sep 2017

Reviewer name: Torsten Struck

Reviewer Comments to Author:

The paper presents transcriptomic libraries for 14 developmental stages of an echiurid species. This is within the scope of the journal. Hence, I suggest accepting the paper after a major revision. My major concern is the introduction. The introduction does not adequately reflect the literature on the position of Echiura. With respect to morphological data the studies of Hessling concerning the nervous system are missing. This among others also reflected in the erroneous statement that the presence of a larval nervous system includes placement in Annelida. However, this is in this way not correct. Larval nervous systems occur throughout the animal kingdom, it is the specific features of this system, which support this placement and require citing the works by Hessling. Moreover, recent studies using phylogenomic data concerning the phylogeny of Annelida are also not mentioned (see below). Finally, for understanding the early evolution of Lophotrochozoa Echiura are irrelevant as they are highly derived annelid taxa. Echiura are more interesting in understanding the plasticity of body plan evolution in general, but this independent of their position within Lophotrochozoa. Moreover, citations supporting the claim that Urechis unicinctus attracted special attention for evo-devo are lacking and should be provided.

Minor changes:
Page 2, Line 6: delete "the phylum"
Page 2, Line 8: change to "unlike most other annelid"
Page 2, Line 16: insert space between "development, ontogenetic"
Page 2, Line 57: insert dot between "stages. These"
Page 4, Line 36: delete dot between "(Fig. 1). This"
Page 4, Line 48: delete "the" at the end of the line
Page 4, Line 51: "profiles" instead of "profiling"
Page 7, Line 2: describe how the k-mer analysis was conducted in detail
Page 7, Lines 18-25: I do not understand what has been done here. Concatenation means that individual sequences are attached to each other one after the other, but I think this is not meant here. Rather assume that the reads from all 14 libraries were pooled together for assembly of the data. Please clarify this point.
Page 8, Line 7: How did you correct the expression profiles for the uneven sequencing depth among the different libraries? The range is from 43 million reads to 140 million and hence the latter is more than three times higher.
Page 8, Line 46: include a comma in "were identified,"

Page 13, Line 24: This is not a phylogenetic tree, which by definition can only be reconstructed between species and not developmental stages between individuals. Change wording.
Page 13, Line 44: "mouth" instead of "mouse"
Page 13, Line 44: insert a space between "50µm. (B)"

Citations missing:
Hessling, R. & Westheide, W. (2002) Are Echiura derived from a segmented ancestor? Immunohistochemical analysis of the nervous system in developmental stages of Bonellia viridis. Journal of Morphology, 252, 100-113.
Hessling, R. (2002) Metameric organisation of the nervous system in developmental stages of Urechis caupo (Echiura) and its phylogenetic implications. Zoomorphology, 121, 221-234.
Hessling, R. (2003) Novel aspects of the nervous system of Bonellia viridis (Echiura) revealed by the combination of immunohistochemistry, confocal laser-scanning microscopy and three-dimensional reconstruction. Hydrobiologia, 496, 225-239.
Struck, T.H., Paul, C., Hill, N., Hartmann, S., Hösel, C., Kube, M., Lieb, B., Meyer, A., Tiedemann, R., Purschke, G. & Bleidorn, C. (2011) Phylogenomic analyses unravel annelid evolution. Nature, 471, 95-98.
Weigert, A., Helm, C., Meyer, M., Nickel, B., Arendt, D., Hausdorf, B., Santos, S.R., Halanych, K.M., Purschke, G., Bleidorn, C. & Struck, T.H. (2014) Illuminating the base of the annelid tree using transcriptomics. Molecular Biology and Evolution, 31, 1391-1401.
Struck, T.H., Golombek, A., Weigert, A., Franke, Franziska A., Westheide, W., Purschke, G., Bleidorn, C. & Halanych, K.M. (2015) The Evolution of Annelids Reveals Two Adaptive Routes to the Interstitial Realm. Current Biology, 25, 1993-1999.
Andrade, S.C.S., Novo, M., Kawauchi, G.Y., Worsaae, K., Pleijel, F., Giribet, G. & Rouse, G.W. (2015) Articulating "archiannelids": Phylogenomics and annelid relationships, with emphasis on meiofaunal taxa. Molecular Biology and Evolution.

Level of Interest

Please indicate how interesting you found the manuscript: An article whose findings are important to those with closely related research interests

Quality of Written English

Please indicate the quality of language in the manuscript: Needs some language corrections before being published

Declaration of Competing Interests

Please complete a declaration of competing interests, considering the following questions:

- Have you in the past five years received reimbursements, fees, funding, or salary from an organisation that may in any way gain or lose financially from the publication of this manuscript, either now or in the future?
- Do you hold any stocks or shares in an organisation that may in any way gain or lose financially from the publication of this manuscript, either now or in the future?
- Do you hold or are you currently applying for any patents relating to the content of the manuscript?
- Have you received reimbursements, fees, funding, or salary from an organization that holds or has applied for patents relating to the content of the manuscript?
- Do you have any other financial competing interests?
- Do you have any non-financial competing interests in relation to this paper?

If you can answer no to all of the above, write 'I declare that I have no competing interests' below. If your reply is yes to any, please give details below.

I declare that I have no competing interests.

I agree to the open peer review policy of the journal. I understand that my name will be included on my report to the authors and, if the manuscript is accepted for publication, my named report including any attachments I upload will be posted on the website along with the authors' responses. I agree for my report to be made available under an Open Access Creative Commons CC-BY license (http://creativecommons.org/licenses/by/4.0/). I understand that any comments which I do not wish to be included in my named report can be included as confidential comments to the editors, which will not be published.

I agree to the open peer review policy of the journal

To further support our reviewers, we have joined with Publons, where you can gain additional credit to further highlight your hard work (see: https://publons.com/journal/530/gigascience). On publication of this paper, your review will be automatically added to Publons, you can then choose whether or not to claim your Publons credit. I understand this statement.

Yes
