# Supplementary material for: The developmental transcriptome atlas of the spoon worm Urechis unicinctus (Echiurida: Annelida)
Source: Gigascience. 2018 Feb 15;7(3):giy007. doi: 10.1093/gigascience/giy007 (PMC5863216; doi:10.1093/gigascience/giy007)
Supplement: Reviewer_3_Report_(Revision_1) -- Torsten Struck — 04 Dec 2017 Reviewed [file giy007_reviewer_3_report_(revision_1).pdf]

Reviewer Report

Title: The developmental transcriptome atlas of the spoon worm Urechis unicinctus (Echiurida: Annelida)

Version: Revision 1 **Date:** 04 Dec 2017

Reviewer name: Torsten Struck

Reviewer Comments to Author:

The manuscript has improved, but still some points need to be addressed:
1.) The authors did not include all the citations I asked to add. If this is due to a citation limit not stated in the "Instruction to Authors" the authors should exclude Struck et al. (2011) and Andrade et al. (2015) as the review by Weigert and Bleidorn cover the recent phylogenomic studies on annelid phylogeny and instead include the two Hessling (2002) papers as they present the results on the nervous system.
2.) Linnean categories like phylum have no biological meaning and are just an artificial categorical level. Therefore, their usage should be restricted to the least possible and whenever possible neutral should be used. Hence, the following changes should be made:
Page 2, Line 2: delete "the phylum"
Page 4, Line 8: "taxon" instead of "phylum"
Page 4, Line 9: "the three major animal taxa" instead of "the three animal phyla"
Page 4, Line 20: "taxon" instead of "phylum"
Page 10, Line 22: "annelid" instead of "phylum"

3.) Minor changes:
Page 4, Line 9: delete "the" before Annelida (Annelida is grammatically a proper name and does not need a definitive article)
Page 7, Lines 6-10: The two sentences are confusing. It sounds like you did one round of sequencing and then another round on the same material. Please rephrase.
Page 9, Line 19: "3C" instead of "3D"
Page 15, Figure legend 3: The description for Figure 3D is lacking.

Level of Interest

Please indicate how interesting you found the manuscript: An article whose findings are important to those with closely related research interests

Quality of Written English

Please indicate the quality of language in the manuscript: Acceptable

Declaration of Competing Interests

Please complete a declaration of competing interests, considering the following questions:

- Have you in the past five years received reimbursements, fees, funding, or salary from an organisation that may in any way gain or lose financially from the publication of this manuscript, either now or in the future?
- Do you hold any stocks or shares in an organisation that may in any way gain or lose financially from the publication of this manuscript, either now or in the future?
- Do you hold or are you currently applying for any patents relating to the content of the manuscript?
- Have you received reimbursements, fees, funding, or salary from an organization that holds or has applied for patents relating to the content of the manuscript?
- Do you have any other financial competing interests?
- Do you have any non-financial competing interests in relation to this paper?

If you can answer no to all of the above, write 'I declare that I have no competing interests' below. If your reply is yes to any, please give details below.

I declare that I have no competing interests.

I agree to the open peer review policy of the journal. I understand that my name will be included on my report to the authors and, if the manuscript is accepted for publication, my named report including any attachments I upload will be posted on the website along with the authors' responses. I agree for my report to be made available under an Open Access Creative Commons CC-BY license (http://creativecommons.org/licenses/by/4.0/). I understand that any comments which I do not wish to be included in my named report can be included as confidential comments to the editors, which will not be published.

I agree to the open peer review policy of the journal

To further support our reviewers, we have joined with Publons, where you can gain additional credit to further highlight your hard work (see: https://publons.com/journal/530/gigascience). On publication of this paper, your review will be automatically added to Publons, you can then choose whether or not to claim your Publons credit. I understand this statement.

Yes
